# Chantriolides F–P, Highly Oxidized Withanolides with Hepatoprotective Activity from *Tacca chantrieri*

**DOI:** 10.3390/molecules27238197

**Published:** 2022-11-24

**Authors:** Yue Yang, Fei Zhou, Min Wang, Mukhammadrizo Turanazarov, Xiao-Rong Wang, Changqiang Ke, Sheng Yao, Ligen Lin, Chunping Tang, Yang Ye

**Affiliations:** 1 State Key Laboratory of Drug Research, Shanghai Institute of Materia Medica, Chinese Academy of Sciences, Shanghai 201203, China; 2 Natural Products Chemistry Department, Shanghai Institute of Materia Medica, Chinese Academy of Sciences, Shanghai 201203, China; 3 State Key Laboratory of Quality Research in Chinese Medicine, Institute of Chinese Medical Sciences, University of Macau, Avenida da Universidade, Taipa, Macau 999078, China; 4 University of Chinese Academy of Sciences, No. 19A Yuquan Road, Beijing 100049, China; 5 Xishuangbanna Research Institute of Nationality Medicine, Xishuangbanna Hospital of Traditional Dai Medicine, No. 8 Zhuangdong Western Road of Xishuangbanna Tourism and Resort Zone, Xishuangbanna 666100, China; 6 School of Life Science and Technology, ShanghaiTech University, 393 Middle Huaxia Road, Pudong, Shanghai 201210, China

**Keywords:** *Tacca chantrieri*, structural elucidation, withanolides, chantriolides F–P, hepatoprotective effect

## Abstract

Eleven highly oxidized withanolides, chantriolides F–P (**1**–**11**), together with six known analogues (**12**–**17**), were isolated from the rhizomes of *Tacca chantrieri*. Their structures were established on the basis of comprehensive spectroscopic data analysis and comparison with published NMR data, and their absolute configurations were further confirmed by experimental ECD data and single crystal X-ray diffraction analysis. The structures of compounds **5**–**8** contained a chlorine atom substituted at C-3. Compounds **1** and **12** are a pair of epimers isomerized at C-24 and C-25, while compounds **9** and **16** are isomerized at C-1, C-7, C-24, and C-25. Next, the hepatoprotective effect of all the isolates was evaluated on *tert*-butyl hydroperoxide (*t*-BHP)-injured AML12 hepatocytes. Compounds **5**–**11** and **16** significantly enhanced cell viability. Compound **8** decreased reactive oxygen species accumulation and increased glutathione level in *t*-BHP injured AML12 hepatocytes through promoting nuclear translocation of nuclear factor erythroid 2-related factor 2 (Nrf2).

## 1. Introduction

Withanolides are a group of highly oxygenated C28-steroidal lactones built on an ergostane skeleton, which are primarily found in the Solanaceae family, particularly in the *Physalis*, *Datura*, *Withania*, and *Nicandra* genera. In addition, withanolides were reported from the Taccaceae, Myrtaceae, Labiataem, Dioscoreaceae, and Asteraceae families, and from the soft corals (marine source) as well. Due to their unique structures [1,2,3,4,5] and diverse biological activities [6,7,8,9,10,11,12,13], withanolides have captured extensive attention, and over the past decade, more than 500 new withanolides of natural origin have been discovered [14,15,16].

Taccaceae is a family of perennial plants distributed mainly in tropical regions. Chemical investigations revealed the existence of steroids, diarylheptanoids and their glucosides, flavonoids, etc. [17,18,19]. Of all the isolated compounds, 13 withanolides were characterized from *Tacca subflabellata*, *Tacca plantaginea*, and *Tacca chantrieri*, namely, taccalonolides O and P [20], plantagiolides A–F [21,22], and K–M [23], and chantriolides D and E [24], together with eight withanolide glucosides, including chantriolides A–C [25,26], and E[27], plantagiolides I, J [28], and N [23], and (22R*,24R*,25S*)-3*β*-[(*O*-*β*-D-glucopyranosyl-(1→4)-*O*-*β*-D-glucopyranosyl-(1→2)-*O*-[*β*-D-glucopyranosyl-(1→6)]-*β*-D-glucopyranosyl)oxy]-22-hydroxyergost-5-en-26-oic acid *δ*–lactone [29]. Most withanolides contain *δ*-lactone rings by oxidization at C-26 and C-22, while taccalonolides O and P, and chantriolide E bear *γ*-lactone rings at C-26 and C-23. Plantagiolide I and chantriolide E are two rare withanolide glucosides having a chlorine atom substituted at C-3.

*Taccaceae chantrieri* is a traditional medicinal plant which has long been used to treat gastric and duodenal ulcers, hepatitis, and hypertension, and it is distributed mainly in Vietnam, Malaysia, Thailand, and southern China. Our previous studies discovered some novel diarylheptanoid dimers from this plant [30], which prompts an in-depth investigation searching for more novel structures with potent bioactivities. A total of 17 withanolides, including 11 new ones, were eventually identified from the rhizomes of *T. chantrieri*. Their structures were determined by extensive analyses of 1D and 2D NMR, HRESIMS data, electronic circular dichroism (ECD) spectra, as well as single-crystal X-ray diffraction studies. All the isolates were evaluated for hepatoprotective effect on *tert*-butyl hydroperoxide (*t*-BHP)-injured AML12 hepatocytes. Herein, the isolation and structure elucidation of new compounds **1**–**11** and their hepatoprotective activities are presented.

## 2. Results and Discussion

### 2.1. Structural Elucidation

**Figure 1:**
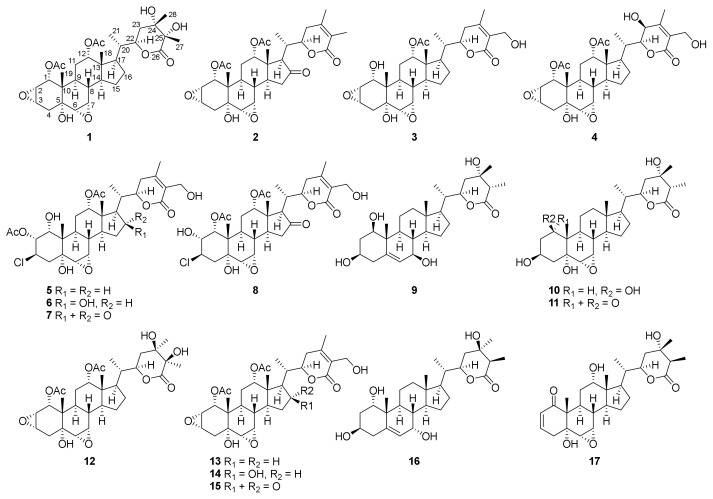
Structures of compounds **1**–**17** from *Taccaceae chantrieri*.

Chantriolide F (**1**) was obtained as colorless crystals from acetonitrile. It had a molecular formula of C_32_H_46_O_11_ according to the HRESIMS data (*m*/*z* 651.3023 ([M + HCOO]^−^, calcd. for 651.3017), corresponding to 10 indices of hydrogen deficiency. The IR spectrum showed absorption bands for hydroxy (3480 cm^−1^) and carbonyl groups (1732 cm^−1^). The ^1^H and ^13^C NMR data (Table 1) of **1** showed characteristics of the withanolide backbone, including four singlet methyls [*δ*_H_ 1.62, 1.62, 0.77, 0.70; *δ*_C_ 25.4, 23.7, 16.7, 12.5], one doublet methyl [*δ*_H_ 1.06 (d, *J* = 6.3 Hz); *δ*_C_ 12.3], seven oxygenated methines, including two *O*-acetylmethines and two epoxy groups [*δ*_H_ 5.11 (m), 5.09 (t, *J* = 3.4 Hz), 4.89 (d, *J* = 5.7 Hz), 3.87 (m), 3.54 (dd, *J* = 3.7, 1.9 Hz), 3.11 (t, *J* = 2.6 Hz), 2.95 (d, *J* = 3.5 Hz); *δ*_C_ 79.7, 76.2, 72.9, 57.0, 55.9, 54,6, 52.0], three oxygenated quaternary carbons (*δ*_C_ 77.5, 73.6, 70.7), and one ester carbonyl (*δ*_C_ 179.2). In addition, two acetyl groups (*δ*_H_ 2.13, 2.04; *δ*_C_ 170.8, 170.7, 21.5, 20.8) were observed. A detailed analysis of 2D NMR spectra of compound **1** (Figure S1 from Supplementary Materials) further indicated that **1** has the same planar structure (Figure 1) as plantagiolide E (**12**) [21].

**Figure 2:**
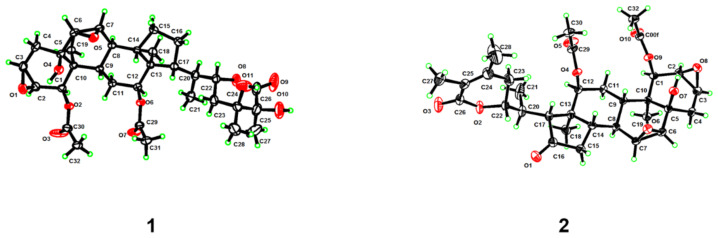
Perspective ORTEP drawings for compounds **1** and **2**.

In the NOESY spectrum, correlations of H_3_-19 with H-1/H-2/H_2_-4*β*/H-6/H-7/H-8/H_2_-11*β*, H-8 with H_3_-18, and H_3_-18 with H_2_-11*β*/H-12 indicated that they were co-facial and arbitrarily designated as *β*-oriented. The coupling constant of H-3 (*δ*_H_ 3.54, dd, *J* = 3.7, 1.9 Hz) implied that H-3 was on the same face with H-2, and also *β*-oriented. In addition, NOESY correlations of H-9 with H-14, H-14 with H-17 showed that these protons were *α*-oriented. The different chemical shifts of compounds **1** and **12** at positions 23, 27 and 28 (**1**: *δ*_H_ 2.20, m, 2.00, m, 1.62, s, 1.62, s, *δ*_C_ 33.5, 23.7, 25.4; **12**: *δ*_H_ 2.84, m, 1.88, m, 1.95, s, 1.79, s, *δ*_C_ 31.9, 19.4, 24.3) suggested a change in the configuration at positions 24 and 25. The configurations of C-5, C-20, C-21, C-22, C-24, and C-25 were established by single-crystal X-ray diffraction analysis with Cu K*α* radiation (CCDC 2203317, Figure 2). Therefore, the structure of **1** was identified as the 24*S*, 25*S* epimer of **12**, as shown in Figure 1.

Compound **2** was isolated as colorless crystals. Its molecular formula, C_32_H_42_O_10_, was deduced from the HRESIMS protonated molecular ion at *m*/*z* 587.2865 ([M + H]^+^, calcd. for C_32_H_43_O_10_, 587.2856). Its NMR data (Table 1) showed high similarities to those of the known compound plantagiolide A (**15**) [21], except that a methyl (*δ*_H_ 1.89; *δ*_C_ 13.1) rather than an oxygenated methylene group was observed for **2**. HMBC correlations (Figure S1 from Supplementary Materials) from this methyl to C-24 (*δ*_C_ 149.6) and C-26 (*δ*_C_ 166.7), and from H_3_-28 (*δ*_H_ 1.74) to C-23 (*δ*_C_ 31.6) and C-25 (*δ*_C_ 122.4) suggested the location of the methyl at C-27. The whole structure of **2** was further confirmed by a single-crystal X-ray crystallographic diffraction experiment with Cu K*α* radiation (CCDC 2203323, Figure 2). Thus, the structure of **2** was proposed as shown, and named chantriolide G.

**Figure 3:**
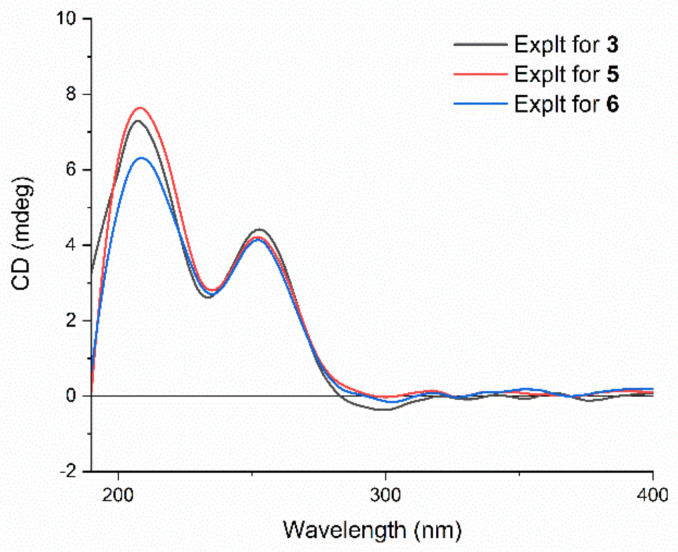
Experimental ECD spectra of compounds **3**, **5**, and **6**.

The molecular formula of **3** was designated by its HRESIMS and ^13^C NMR data as C_30_H_42_O_9_, which was 42 Da less than that of the known plantagiolide C (**13**) [21]. The proton NMR showed the chemical shift of H-1 of **3** at *δ*_H_ 3.83 (m), which was shifted 0.71 ppm upfield compared with that of plantagiolide C, suggesting the miss of acetyl group at C-1. The ^1^H–^1^H COSY correlations (Figure S1 from Supplementary Materials) of H-1/H-2/H-3/H_2_-4, and the HMBC correlations from H-1 (*δ*_H_ 3.83) to C-5 (*δ*_C_ 71.8), and C-10 (*δ*_C_ 40.4), from H_3_-19 (*δ*_H_ 0.69) to C-1 (*δ*_C_ 70.4), C-5 (*δ*_C_ 71.8), and C-9 (*δ*_C_ 29.2) confirmed the assignment. The NOE correlation of H_3_-19 and H-1 indicated *β*-orientation of H-1. The *R*-configuration at the C-22 was confirmed by a positive Cotton effect at 250 nm in the ECD spectrum (Figure 3) of the *α*,*β*-unsaturated *δ*-lactone [31,32]. The same 22*R*-configuration was designated not only for compounds **2** and **3**, but also for compounds **5**–**8** due to the fact that each showed a positive Cotton effect around 250 nm in their ECD spectra. Accordingly, the structure of **3** was proposed as shown and was named chantriolide H.

**Figure 4:**
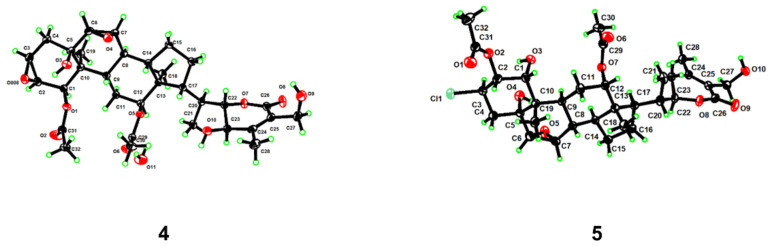
Perspective ORTEP drawings for compounds **4** and **5**.

Chantriolide I (**4**), colorless crystals, had a molecular formula of C_32_H_44_O_11_ established by the HRESIMS data (*m*/*z* 622.3237 [M + NH_4_]^+^, calcd. for 622.3227) and the ^13^C NMR data. Its NMR data (Table 1) also showed high similarities to those of the known plantagiolide C (**13**) [21] except for the presence of an oxygenated methine [*δ*_H_ 4.27 (m); *δ*_C_ 66.7] taking the place of a methylene group in **13**. The ^1^H–^1^H COSY correlations (Figure S1 from Supplementary Materials) between H-22 (*δ*_H_ 4.29) and H-23 (*δ*_H_ 4.27) suggested that the hydroxyl group was located at C-23. The absolute configuration of **4** including the *S*-configuration of C-23 was established by a single-crystal X-ray crystallographic diffraction experiment with Cu K*α* radiation (CCDC 2203333, Figure 4).

**Figure 5:**
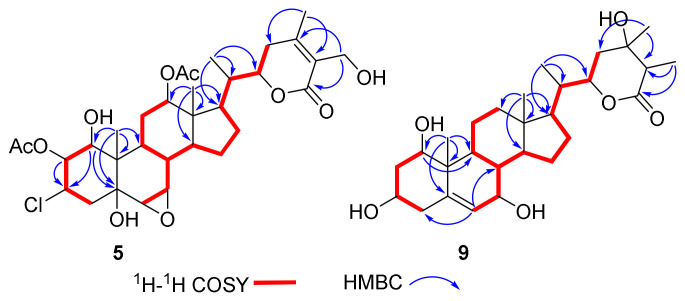
Key ^1^H-^1^H COSY and HMBC correlations for compounds **5** and **9**.

**Figure 6:**
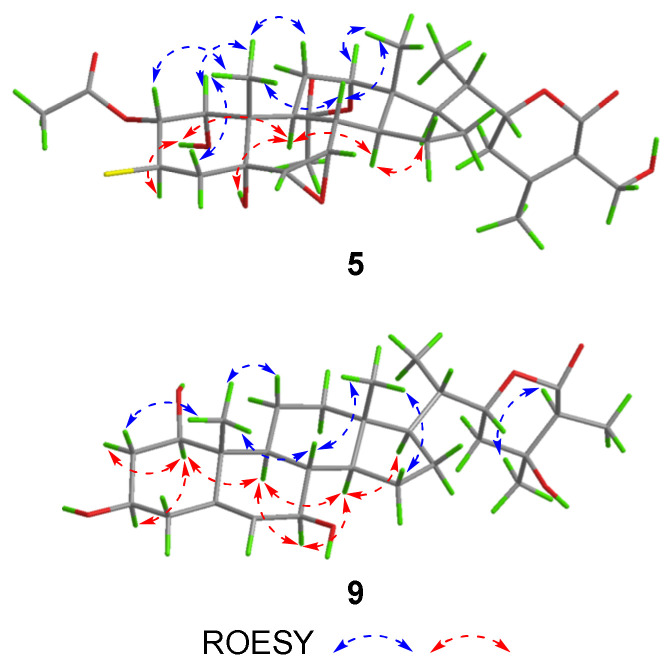
Key ROESY correlations for compounds **5** and **9**.

The HRESIMS spectrum of compound **5** showed pseudomolecular ions at *m*/*z* 669.2689 and 671.2675 with a ratio being 3:1, indicating the presence of a chlorine atom and a molecular formula of C_32_H_45_O_10_Cl. Such a mass spectral pattern (*m*/*z* M:M+2 = 3:1) was also observed for compounds **6**–**8**, suggesting that these compounds also contained a chlorine atom in the molecules. The proton NMR data of **5** (Table 2), almost identical to those of **3** (Table 1), were indicative of a withanolide structure as well, which involved characteristic signals of two acetyl signals at *δ*_H_ 2.17, 1.94, four methyl signals at *δ*_H_ 2.11 (s), 1.00 (d, *J* = 6.7 Hz), 0.91 (s), 0.73 (s), two acetoxylated methine signals at *δ*_H_ 5.73 (dd, *J* = 10.9, 3.9 Hz), 5.25 (m), two oxygenated methines at *δ*_H_ 4.40 (dt, *J* = 13.2, 3.5 Hz), 4.09 (dd, *J* = 10.0, 3.9 Hz), two epoxy methine signals at *δ*_H_ 3.27 (t, *J* = 3.0 Hz), 3.04 (d, *J* = 3.7 Hz), and one oxygenated methylene signal at *δ*_H_ 4.87 (d, *J* = 11.7 Hz), 4.77 (d, *J* = 11.7 Hz). Compared with **3**, compound **5** has one more acetyl group and one chlorine substituted methine. The HMBC correlations (Figure 5) between *δ*_H_ 5.73 (H-2) and the carbonyl carbon at *δ*_C_ 171.0 indicate that this acetyl group was located at C-2. In addition, the ^13^C NMR chemical shift of C-3 (*δ*_C_ 57.2), together with the ^1^H-^1^H COSY correlations of H-1/H-2/H-3/H_2_-4, and HMBC correlations from H-1 (*δ*_H_ 4.09) to C-3 (*δ*_C_ 57.2), suggested the location of the chlorine atom at C-3. The relative configuration of H-2 and chlorine at C-3 was determined as *β* by the ROESY correlations of H_3_-19/H-2 and OH-1/H-3 (Figure 6). The whole structure of **5** was further confirmed by a single crystal crystallographic diffraction experiment with Cu K*α* radiation (CCDC 2203335, Figure 4), and named chantriolide J.

Compound **6** gave protonated molecular ion peaks at *m*/*z* 641.2735 (calcd. 641.2729) in the HRESIMS, corresponding to a molecular formula of C_32_H_45_O_11_Cl, which differed from that of **5** by one additional oxygen atom. The ^1^H and ^13^C NMR spectra of **6** were highly similar to those of **5**. The only difference between these two compounds is that a methylene group in **5** was replaced by an oxygenated methine (*δ*_H_ 4.40, d, *J* = 11.7 Hz; *δ*_C_ 70.1) and a hydroxyl signal (*δ*_H_ 6.37, d, *J* = 4.5 Hz) in **6**. The ^1^H-^1^H COSY correlations of H_2_-15/H-16/H-17 and HMBC correlations (Figure S1 from Supplementary Materials) between the hydroxyl proton resonance at *δ*_H_ 6.37 and the methine carbon at *δ*_C_ 49.3 (C-17) suggested that the hydroxyl group was attached to C-16. The ROESY correlations (Figure S1 from Supplementary Materials) of H_3_-19 with H-1/H-2/H_2_-4*β*/H-8/H_2_-11*β*, H-8 with H_3_-18, and H_3_-18 with H_2_-11*β*/H-12/H_2_-15*β*, and H_2_-15*β* with OH-16 indicated that they were co-facial, and *β*-oriented. The coupling constants of H-6 (*δ*_H_ 3.05, d, *J* = 3.7 Hz) and H-7 (*δ*_H_ 3.31, t, *J* = 3.1 Hz) indicated that the protons at C-6 and C-7 were also *β*-oriented. In addition, ROESY correlations of OH-1/H-3/H-9, OH-5/H_2_-4*α*/H-9, H-9/H-14, and H-14/H-17 showed that these protons were *α*-oriented. The absolute configuration of **6** was further confirmed by its ECD spectrum, which showed the similar Cotton effects to compound **5** (Figure 3). Therefore, compound **6** was defined as a 16*β*-hydroxyl derivative of **5**, and named chantriolide K.

**Figure 7:**
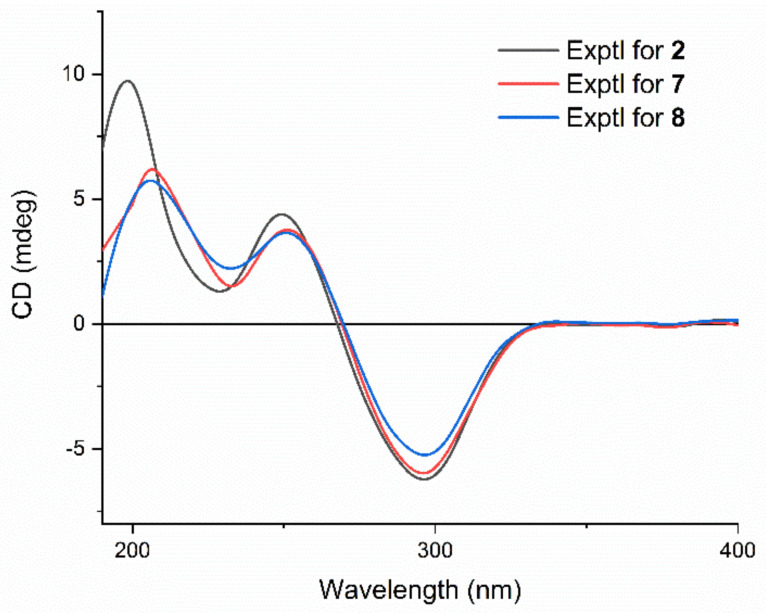
Experimental ECD spectra of compounds **2**, **7**, and **8**.

The HRESIMS and ^13^C NMR data of chantriolide L (**7**) showed a molecular formula of C_32_H_43_O_11_Cl, indicating a mass 2 Da less than that of **6**. The ^13^C NMR spectrum showed one additional carbonyl signal at 216.1 ppm, suggesting the presence of one carbonyl group in **7**. The HMBC correlations (Figure S1 from Supplementary Materials) from the protons at *δ*_H_ 2.60 (H-14) and *δ*_H_ 2.47 (H-20) to the carbon at *δ*_C_ 216.1 (C-16) placed the carbonyl at C-16. The stereochemistry of **7** was established from the ROESY correlations (Figure S1 from Supplementary Materials) and the ECD spectrum (Figure 7). Compound **7** showed very similar Cotton effects to compound **2**, while both of them showed an additional negative Cotton effect around 300 nm when compared with compounds **3**, **5**, and **6**, which was obviously due to the presence of the carbonyl at C-16.

Compound **8** gave a molecular formula C_32_H_43_O_11_Cl, as determined by the positive ion HRSEIMS at *m*/*z* 639.2564 [M + H]^+^ (calcd. for C_32_H_44_ClO_11_, 639.2567), which was the same with that of compound **7**. The ^1^H NMR data of both **7** and **8** showed four methyls, two acetoxyl methyls, six oxygenated methines, and one oxygenated methylene. Compared with **7**, the chemical shifts at H-1, H-2, and H-3 in compound **8** shifted from *δ*_H_ 4.08, 5.79, and 5.10 to *δ*_H_ 5.11, 4.16, and 4.48 (Table 2), together with the HMBC correlation from the proton at *δ*_H_ 5.11 (H-1) to the carbonyl carbon at *δ*_C_ 171.5, revealed that **8** possessed an acetoxy group at C-1 and a hydroxy group at C-2. The ROESY (Figure S2 from Supplementary Materials) and ECD (Figure 7) spectra of **8** were very close to those of **7**, indicating further that they had the same relative and absolute configuration. Consequently, the structure of compound **8** was proposed, and given a trivial name chantriolide M.

**Figure 8:**
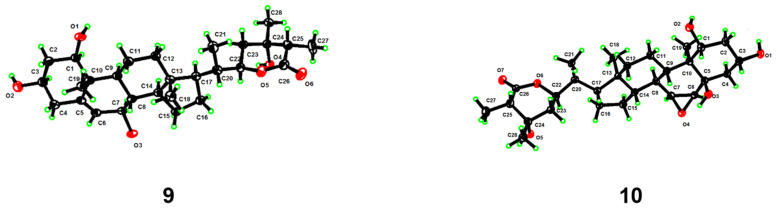
Perspective ORTEP drawings for compounds **9** and **10**.

Chantriolide N (**9**), colorless crystals, was assigned a molecular formula of C_28_H_44_O_6_ by HRESIMS and ^13^C NMR data, which is the same as that of plantagiolide M (**16**) [23]. Its 1D and 2D NMR data (Table 3, Figure 5) further established a same planar structure with **16**. The key ROESY correlations of H-1/H-3/H-9, H-9/H-7, and H-7/H-14 unveiled that the relative configurations at C-1 and C-7 in **9** changed when compared with those of compound **16**. Such elucidation was further supported by the difference of chemical shifts at H-1 (**9**: 3.83, dd, *J* = 11.9, 4.2 Hz; **16**: 3.45, dd, *J* = 11.9, 4.3 Hz) and H-7 (**9**: 4.10, d, *J* = 8.3 Hz; **16**: 3.79, dd, *J* = 5.8, 3.5 Hz). In addition, distinct proton chemical shifts were observed at 23 (**9**: 1.98, m, 1.90, m; **16**: 2.27, m, 1.79, m), 27 (**9**: 1.65, d, *J* = 7.0 Hz; **16**: 1.29, d, *J* = 7.1 Hz), and 28 (**9**: 1.56, s; **16**: 1.35, s). The absolute configuration was further confirmed by a single crystal crystallographic diffraction experiment with Cu K*α* radiation (CCDC 2203338, Figure 8). Accordingly, the structure of **9** was identified as a 1*R*,7*R*,24*S*,25*S*-epimer of **16**, and named chantriolide N.

The molecular formula of compound **10** was determined to be C_28_H_44_O_7_ by HRESIMS analysis (*m*/*z* 493.3180 [M + H]^+^, calcd. for 493.3165), which contains one more oxygen atom than **9**. The ^1^H and ^13^C NMR data (Table 3) of **10** showed characteristic signals of two epoxy methine signals [*δ*_H_ 3.26 (dd, *J* = 3.8, 2.4 Hz), 3.14 (d, *J* = 3.8 Hz); *δ*_C_ 59.4, 57.8], and one oxygenated quaternary carbon signal (*δ*_C_ 72.9). The location of this epoxy group at C-6 and C-7 was deduced by the ^1^H-^1^H COSY correlations of H-6/H-7/H-8. Furthermore, the C-5 was designated as an oxygenated quaternary carbon by the HMBC correlations (Figure S1 from Supplementary Materials) from the hydroxyl proton at 4.69 ppm (5-OH) to C-4 (*δ*_C_ 43.9), C-5 (*δ*_C_ 72.9), and C-6 (*δ*_C_ 59.4). The whole structure was confirmed through a single crystal crystallographic diffraction experiment with Cu K*α* radiation (CCDC 2203339, Figure 8). A trivial name chantriolide O was given to **10**, and its structure is shown in Figure 1.

**Figure 9:**
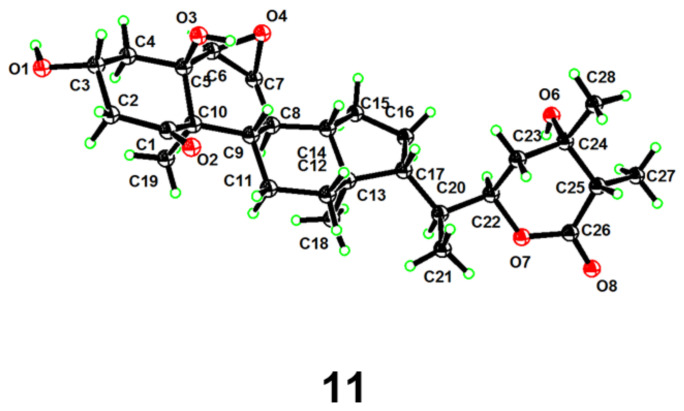
Perspective ORTEP drawings for compound **11**.

Chantriolide P (**11**) was obtained as colorless crystals and assigned a molecular formula of C_28_H_42_O_7_ from its HRESIMS and ^13^C NMR data, corresponding to eight indices of hydrogen deficiency. Its molecular weight was 2 Da less than that of chantriolide O (**10**). Its carbon NMR data (Table 3), when compared with that of **10**, showed the presence of one additional ketone group (*δ*_C_ 210.6), which was designated as C-1 by the HMBC correlation (Figure S1 from Supplementary Materials) from Me-19 at *δ*_H_ 1.30 and H_2_-2 at *δ*_H_ 3.33, 3.04 to this carbon. The relative configuration was deduced by the ROESY correlations, and the whole structure was further confirmed by a single crystal crystallographic diffraction experiment with Cu K*α* radiation (CCDC 2203341, Figure 9). Subsequently, the structure of compound **11** was established, and named chantriolide P.

Apart from compounds **1**–**11**, six other known compounds were isolated and identified as plantagiolide E (**12**) [21], plantagiolide C (**13**) [21], plantagiolide B (**14**) [21], plantagiolide A (**15**) [21], plantagiolide M (**16**) [23], and chantriolide D (**17**) [24] by comparing their spectroscopic data with those reported in literature.

### 2.2. Hepatoprotective Effect Assay

**Figure 10:**
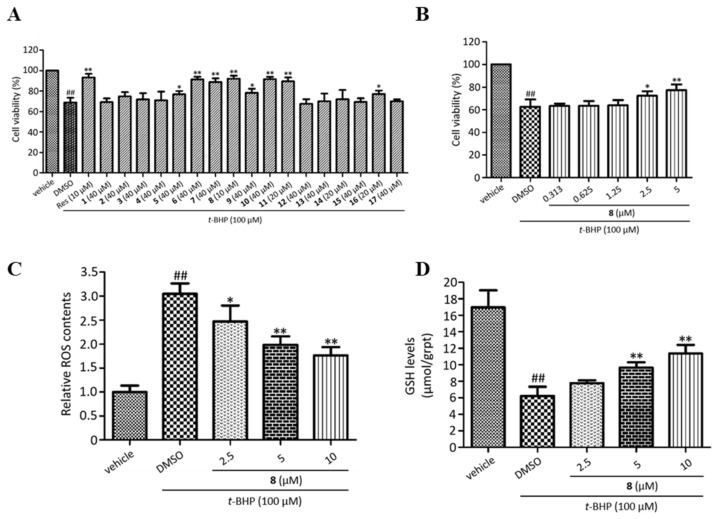
Hepatoprotective effects of withanolides on *t*-BHP-injured AML12 hepatocytes. (**A**) Cell viability of *t*-BHP-injured AML12 hepatocytes treated with compounds **1**–**17** at their maximum safe concentration. Resveratrol (Res) at 10 μM was used as a positive control. (**B**) Cell viability of *t*-BHP-injured AML12 hepatocytes treated with different concentrations of compound **8**. (**C**) ROS contents in *t*-BHP-injured AML12 hepatocytes treated with different concentrations of compound **8**. (**D**) GSH levels in *t*-BHP-injured AML12 hepatocytes treated with different concentrations of compound **8**. Data are shown as mean ± S.D., *n* = 3. ^##^ *p* < 0.01 vs. vehicle; * *p* < 0.05 and ** *p* < 0.01 vs. *t*-BHP plus DMSO.

The rhizomes of *T. chantrieri* have been used for the treatment of hepatitis [33]. Several withanolides were reported to protect hepatocytes against oxidative injury in H_2_O_2_-treated LO2 [34] or AML12 cells [35]. Herein, the hepatoprotective effect of all the isolates were evaluated on *t*-BHP-injured AML12 hepatocytes. Firstly, the noncytotoxic concentrations of compounds **1**–**17** on AML12 hepatocytes were evaluated by the MTT [3-(4,5-dimethylthiazol-2-yl)-2,5-diphenyltetrazolium bromide] assay. The MTT results showed that compounds **1**–**7**, **9**, **10**, **12**, **13**, **15**, and **17** did not show evident cytotoxicity up to 40 μM, while the maximum safe concentrations of compounds **11**, **14**, and **16** were 20 μM, and that of compound **8** was 10 μM (Figure S103 from Supplementary Materials). *t*-BHP at 100 μM significantly decreased the viability of AML12 hepatocytes (*p* < 0.01); whereas, compounds **5**–**11** and **16** at their maximum safe concentration obviously increased cell viability compared to the *t*-BHP group, and resveratrol (Res) at 10 μM was used as a positive control (Figure 10A). Furthermore, compound **8** dose-dependently increased the viability of *t*-BHP-injured AML12 hepatocytes (Figure 10B). *t*-BHP treatment observably increased reactive oxygen species (ROS) accumulation and decreased glutathione (GSH) level, while compound **8** dose-dependently reversed the changes (Figure 10C,D). The above results indicated that compound **8** protected AML12 hepatocytes against *t*-BHP injury by decreasing ROS accumulation and increasing the GSH level.

**Figure 11:**
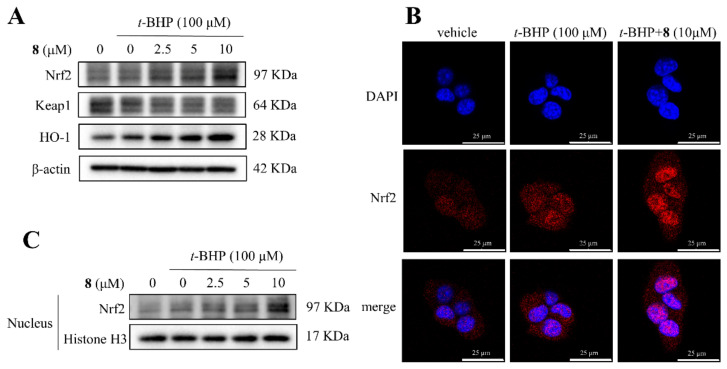
Compound **8** activates Keap-1/Nrf2/HO-1 signaling pathway in *t*-BHP-injured AML12 hepatocytes. (**A**) The total protein levels of Keap-1, Nrf2, and HO-1 were analyzed by Western blots. β-actin was used as a loading control. (**B**) Representative immunofluorescent image of Nrf2 distribution. Nuclei were visualized using DAPI (blue), and Nrf2 was stained as red. Scale bar = 25 µm. (**C**) Nuclear Nrf2 expression was analyzed by Western blots. Histone H3 was used as a loading control.

The nuclear factor erythroid 2–related factor 2 (Nrf2), is the main regulator of the oxidative stress response [36,37]. Under homeostatic conditions, Nrf2 is kept inactive being bound to its endogenous inhibitor, Kelch-like ECH-associated protein 1 (Keap-1) [38]. Under oxidative stress, Nrf2 detaches from Keap-1 and translocates to the nucleus, inducing the expression of antioxidant genes [39]. Heme oxygenase-1 (HO-1) acts as an important antioxidant enzyme to maintain redox homeostasis [40]. Herein, compound **8** at 2.5, 5, and 10 μM decreased the protein expression of Keap-1, and increased the protein expression of Nrf2 and HO-1 in *t*-BHP-injured AML12 hepatocytes (Figure 11A). Furthermore, compound **8** increased the nuclear translocation of Nrf2 assessed by immunofluorescent images and Western blotting (Figure 11B,C). Thus, compound **8** decreased ROS accumulation and increased GSH level by regulating the Keap-1/Nrf2/HO-1 pathway in *t*-BHP-injured AML12 hepatocytes.

In summary, a phytochemical investigation of *T. chantrieri* led to the isolation of 17 withanolides, including 11 new derivatives. Most of them contain an acetoxyl or hydroxyl group attached to C-1 and the epoxy moiety at C-3/C-4 or C-6/C-7. Compounds **5**–**8** have rare substitutions of chlorine atoms, which are unusual for withanolide-type natural products. The absolute configuration for the new withanolides was confirmed by single X-ray diffraction crystallography and electronic circular dichroism analysis. The isolates were evaluated for their hepatoprotective activity on *t*-BHP-injured AML12 hepatocytes. Compounds **5**–**11** and **16** significantly increased viability of *t*-BHP-injured cells. Additionally, compound **8**, the representative withanolide with the best hepatoprotective activity, was proved to decrease ROS accumulation and increase GSH level by regulating the Keap-1/Nrf2/HO-1 pathway in *t*-BHP-injured AML12 hepatocytes.

## 3. Materials and Methods

### 3.1. General Experimental Procedures

Rudolph Research Analytical Autopol VI automatic polarimeter was used to optical rotations values. IR spectra were acquired on a Nicolet Magna FRIR-750 spectrometer. ECD spectra were obtained on a JASCO J-810 spectrometer. HRESIMS data were recorded on the Waters Synapt G2-Si Q-Tof and Agilent G6520 Q-Tof mass spectrometers. 1D and 2D NMR spectra were recorded using a BrukerAvance III-500 (600) spectrometer and a Varian MR-400 spectrometer. The chemical shift (*δ*) values are given in ppm with coupling constants (*J*) in hertz, and the residual signals of Pyridine and CHCl_3_ were used as internal standards. Single-crystal X-ray diffraction measurements were conducted on a Bruker D8 Venture diffractometer or a Bruker Apex-II CCD diffractometer. LCESIMS data were recorded on a Waters 2695 instrument with a 2998 PDA detector equipped with a Waters Acquity ELSD, and a Waters 3100 SQDMS detector. Preparative HPLC was performed on a Varian PrepStar system with an Alltech 3300 ELSD with a Waters Sunfire RP C_18_, 5 μm, 30 × 150 mm column. MCI gel CHP20P (75–150 μm, Mitsubishi Chemical Industries, Tokyo, Japan), silica gel (100–200, 200–300, and 300–400 mesh, Qingdao Marine Chemical Industrials, Qingdao, China), ODS gel AAG12S50 (12 nm, S-50 μm, YMC, Japan) and Sephadex LH-20 (Pharmacia Biotech AB, Uppsala, Sweden) was used for column chromatography. TLC was performed on pre-coated silica gel GF254 plates (Yantai Chemical Industrials, Yantai, China), and the TLC spots were visualized with 5% H_2_SO_4_ in EtOH containing 10 mg/mL vanillin, followed by heating.

### 3.2. Plant Material

The rhizomes of *T. chantrieri* were collected from Jinghong City, Xishuangbanna Dai Autonomous Prefecture, Yunnan Province, China, in October 2016, and identified by one of the authors (Xiao-Rong Wang). A voucher specimen (no. 20161006) was deposited in the Herbarium of the Shanghai Institute of Materia Medica, Chinese Academy of Sciences.

### 3.3. Extraction and Isolation

The air-dried and ground rhizomes of *T. chantrieri* (15 kg) were extracted with 95% EtOH (4 × 40 L, 3 days each) at room temperature. The percolates were combined and evaporated under pressure to afford a crude extract (1.4 kg), which was then suspended in water and partitioned successively with petroleum ether, EtOAc, and *n*-BuOH. The EtOAc fraction (90 g) was separated with MCI column (EtOH/H_2_O, from 30 to 95%, and finally acetone), yielding five fractions (Fr. 1–Fr. 5). Fr. 2 was then treated with a silica gel column (200–300 mesh) eluted with a mixture of CH_2_Cl_2_/acetone (20:1, 15:1, 10:1, 8:1, 6:1, 5:1, 2:1) to give Fr. 2A–2M. Fr. 2I was separated on Sephadex LH-20 (eluted with MeOH) to afford 2I1–2I6. Fr. 2I3 was further purified by Sephadex LH-20 gel (eluted with MeOH) to give subfractions 2I3A–2I3D. Fr. 2I3B was subjected to a silica gel (300–400 mesh) column chromatography using a gradient solvent system of CH_2_Cl_2_/MeOH (80:1, 60:1, 50:1, 20:1, 5:1) to give seven subfractions (2I3B1–2I3B7). Compound **2** (15 mg, *t*_R_ = 13.07 min) was isolated from Fr. 2I3B2 by preparative HPLC (MeCN/H_2_O: 0–120 min, from 22 to 52%). Subfractions Fr. 2J1–Fr. 2J5 were obtained from Fr. 2J via using a Sephadex LH-20 column (eluted with MeOH). Fr. 2J1 was separated by CC over ODS gel (MeOH/H_2_O: from 40 to 70%, and 100%) to yield subfractions 2J1A–Fr. 2J1D. Fr. 2J1A was subjected to preparative HPLC using MeCN/H_2_O as the mobile phase (0–120 min, from 15 to 45%), to afford compound **12** (79 mg, *t*_R_ = 11.02 min). Compounds **1** (30 mg, *t*_R_ = 11.22 min) and **13** (336 mg, *t*_R_ = 11.52 min) were obtained from Fr. 2J1B by preparative HPLC (MeCN/H_2_O: 0–120 min, from 16 to 46%). Fr. 2J2A–Fr. 2J2K were obtained from Fr. 2J2 by CC over ODS gel (MeOH/H_2_O: from 40 to 60%, and 100%). Fr. 2J2F was further purified by preparative HPLC (MeCN/H_2_O: 0–120 min, from 18 to 48%) to give compound **11** (3 mg, *t*_R_ = 12.08 min). Fr. 2K was separated by column chromatography (CC) over Sephadex LH-20 (eluted with MeOH) to afford subfractions 2K1–2K7. Then, Fr. 2K1 and Fr. 2K2 were separated by a silica gel column (200–300 mesh, CH_2_Cl_2_/acetone) to give 2K1A–2K1K. Fr. 2K1E was treated with an ODS column (MeOH/H_2_O: from 35 to 75%, and 100%) and then purified by preparative HPLC using MeCN/H_2_O (0–120 min, from 18 to 48%) to afford compounds **7** (42 mg, *t*_R_ = 12.57 min), **8** (4 mg, *t*_R_ = 10.17 min), and **17** (10 mg, *t*_R_ = 11.98 min). Fr. 2K1F was subjected to the preparative HPLC using MeCN/H_2_O (0–120 min, from 11 to 41%) to yield compound **15** (86 mg, *t*_R_ = 10.03 min). Fr. 2K1G was separated by a Sephadex LH-20 column (eluted with MeOH) and then purified by an ODS column eluted with a gradient of aqueous MeOH (from 40 to 80%, and 100%), affording six fractions (2K1G1A–2K1G1F). Compounds **3** (7 mg, *t*_R_ = 10.68 min) and **10** (4 mg, *t*_R_ = 10.25 min) were isolated from Fr. 2K1G1A and Fr. 2K1G1B by preparative HPLC (MeCN/H_2_O: 0–120 min, from 16 to 36% and 18 to 48%, respectively). Fr. 2K1G1B5 was further purified with preparative TLC (CH_2_Cl_2_/MeOH = 14:1) to yield compound **6** (31 mg, *t*_R_ = 10.02 min). Fr. 2K1H was subjected to an ODS column (MeOH/H_2_O: from 30 to 65%, and 100%) to give subfractions 2K1H1–2K1H11. Fr. 2K1H3 and Fr. 2K1H5 were purified by preparative HPLC (MeCN/H_2_O: 0–120 min, from 10 to 40%, and 12 to 42%, respectively) to afford compounds **4** (18 mg, *t*_R_ = 10.33 min) and **14** (261 mg, *t*_R_ = 9.63 min). Subfractions 2L1–2L8 were yielded from Fr. 2L by using an ODS gel column eluted with aqueous MeOH (from 35 to 80%, and 100%). Fr. 2L5 was subjected to the preparative HPLC using MeCN/H_2_O (0–120 min, from 15 to 45%) to give Fr. 2L5A. Compounds **9** (25 mg, *t*_R_ = 10.52 min) and **16** (24 mg, *t*_R_ = 11.02 min) were obtained from Fr. 2L5A by a silica gel column (CH_2_Cl_2_/MeOH = 15:1). Fr. 3 was separated on a Sephadex LH-20 (eluted with CHCl_3_/MeOH 1:1), yielding Fr. 3A–3G. Fr. 3B was subsequently treated with an ODS column eluted with aqueous MeOH (from 45 to 80%, and 100%) to give fractions Fr. 3B1–3B11. Subfracions Fr. 3B5A–5I were yielded from Fr. 3B5 by using a silica gel column eluted with a mixture of CH_2_Cl_2_/MeOH (80:1, 60:1, 30:1, 15:1, 5:1). Fr. 3B5E was purified with preparative HPLC using MeCN/H_2_O (0–120 min, from 35 to 55%) to afford compound **5** (2 mg, *t*_R_ = 14.52 min).

Chantriolide F (**1**): Colorless crystals (acetonitrile); mp 296–297 °C; [*α*]^20^_D_ + 72 (*c* 1.3, MeOH); IR (KBr) *ν*_max_ 3480, 2920, 1732, 1605, 1383, 1249, 1136, 1028 cm^−1^; ^1^H and ^13^C NMR, see Table 1; HRESIMS *m*/*z* 651.3023 [M + HCOO]^−^ (calcd. for C_33_H_47_O_13_, 651.3017).

Chantriolide G (**2**): Colorless crystals (acetonitrile); mp 289–290 °C; [*α*]^20^_D_ + 16 (*c* 0.8, MeOH); UV (MeOH) *λ*_max_ (log *ε*) 204 (2.64), 223 (2.63),; IR (KBr) *ν*_max_ 3492, 2956, 2921, 2851, 1735, 1701, 1462, 1378, 1244, 1028 cm^−1^; ^1^H and ^13^C NMR, see Table 1; HRESIMS *m/z* 587.2865 [M + H]^+^ (calcd. for C_32_H_43_O_10_, 587.2856).

Chantriolide H (**3**): White powder; [*α*]^20^_D_ + 53 (*c* 1.4, MeOH); UV (MeOH) *λ*_max_ (log *ε*) 203 (2.49); IR (KBr) *ν*_max_ 3474, 2955, 2925, 1737, 1704, 1380, 1247, 1027 cm^−1^; ^1^H and ^13^C NMR, see Table 1; HRESIMS *m*/*z* 547.2914 [M + H]^+^ (calcd. for C_30_H_43_O_9_, 547.2907).

Chantriolide I (**4**): Colorless crystals (acetonitrile); mp 247–248 °C; [*α*]^20^_D_ + 46 (*c* 1.3, MeOH); IR (KBr) *ν*_max_ 3470, 2958, 2924, 1737, 1379, 1258, 1029 cm^−1^; ^1^H and ^13^C NMR, see Table 1; HRESIMS *m*/*z* 622.3237 [M + NH_4_]^+^ (calcd. for C_32_H_48_NO_11_, 622.3227).

Chantriolide J (**5**): Colorless crystals (acetonitrile); mp > 260 °C; [*α*]^20^_D_ − 6 (*c* 0.9, MeOH); UV (MeOH) *λ*_max_ (log *ε*) 203 (2.77); IR (KBr) *ν*_max_ 3445, 2965, 2925, 1738, 1382, 1246, 1026, 736 cm^−1^; ^1^H and ^13^C NMR, see Table 2; HRESIMS *m*/*z* 669.2689 [M + HCOO]^−^ (calcd. for C_33_H_46_ClO_12_, 669.2678).

Chantriolide K (**6**): White powder; [*α*]^20^_D_ + 21 (*c* 1.1, MeOH); UV (MeOH) *λ*_max_ (log *ε*) 214 (2.59); IR (KBr) *ν*_max_ 3449, 2955, 2924, 1732, 1714, 1698, 1382, 1244, 1028 cm^−1^; ^1^H and ^13^C NMR, see Table 2; HRESIMS *m*/*z* 641.2735 [M + H]^+^ (calcd. for C_32_H_46_ClO_11_, 641.2729).

Chantriolide L (**7**): White powder; [*α*]^20^_D_ − 66 (*c* 1.2, MeOH); UV (MeOH) *λ*_max_ (log *ε*) 200 (2.61); IR (KBr) *ν*_max_ 3466, 2958, 2925, 1733, 1382, 1244, 1029 cm^−1^; ^1^H and ^13^C NMR, see Table 2; HRESIMS *m*/*z* 639.2570 [M + H]^+^ (calcd. for C_32_H_44_ClO_11_, 639.2567).

Chantriolide M (**8**): White powder; [*α*]^20^_D_ − 78 (*c* 0.9, MeOH); UV (MeOH) *λ*_max_ (log *ε*) 202 (2.74); IR (KBr) *ν*_max_ 3445, 2957, 2924, 1738, 1715, 1394, 1384, 1244, 1041 cm^−1^; ^1^H and ^13^C NMR, see Table 2; HRESIMS *m*/*z* 639.2564 [M + H]^+^ (calcd. for C_32_H_44_ClO_11_, 639.2567).

Chantriolide N (**9**): Colorless crystals (acetonitrile); mp 262–263 °C; [*α*]^20^_D_ + 20 (*c* 1.2, MeOH); IR (KBr) *ν*_max_ 3406, 2960, 2921, 2847, 1467, 1384, 1068, 1019 cm^−1^; ^1^H and ^13^C NMR, see Table 3; HRESIMS *m*/*z* 499.3026 [M + Na]^+^ (calcd. for C_28_H_44_O_6_Na, 499.3036).

Chantriolide O (**10**): Colorless crystals (acetonitrile); mp 227–229 °C; [*α*]^20^_D_ –18 (*c* 1.3, MeOH); IR (KBr) *ν*_max_ 3462, 2956, 2923, 2869, 1463, 1456, 1372, 1260, 1079, 1015 cm^−1^; ^1^H and ^13^C NMR, see Table 3; HRESIMS *m*/*z* 493.3180 [M + H]^+^ (calcd. for C_28_H_45_O_7_, 493.3165).

Chantriolide P (**11**): Colorless crystals (acetonitrile); mp 279–281 °C; [*α*]^20^_D_ + 53 (*c* 1.5, MeOH); IR (KBr) *ν*_max_ 3466, 2955, 2925, 2870, 2853, 1713, 1699, 1462, 1456, 1379, 1098 cm^−1^; ^1^H and ^13^C NMR, see Table 3; HRESIMS *m*/*z* 508.3284 [M + NH_4_]^+^ (calcd. for C_28_H_46_NO_7_, 508.3274).

### 3.4. X-ray Crystallographic Analysis of Compounds ***1***, ***2***, ***4***, ***5***, ***9***, ***10***, and ***11***

The crystals were obtained from their MeCN solutions, and suitable crystals were selected for X-ray crystallographic analysis. The structures were settled and refined by using the Bruker SHELXTL Software Package. Copies of crystallographic data of every crystal can be obtained free of charge via the internet at www.ccdc.cam.ac.uk/conts/retrieving.html or on application to CCDC, 12 Union Road, Cambridge CB2 1EZ, UK [Tel.: (+44)-1223-336-408; Fax: (+44)-1223-336-033; E-mail: deposit@ccdc.cam.ac.uk].

*Crystal data for Compound***1**. The crystal was kept at 170 K during data collection. C_32_H_46_O_11_: *M* = 606.69 g/mol, orthorhombic, space group *P*2_1_2_1_2_1_ (no. 19), *a* = 9.3329(6) Å, *b* = 11.1622(7) Å, *c* = 29.548(2) Å, *α* = 90°, *β* = 90°, *γ* = 90°, *V* = 3078.2(3) Å^3^, *Z* = 4, *T* = 170 K, *μ*(Cu K*α*) = 0.812 mm^−1^, *F* = 1304.0, *D*_calc_ = 1.309 g/cm^3^, 42,286 reflections measured (5.982° ≤ 2*σ* ≤ 149.77°), 6295 unique (*R*_int_ = 0.0470, *R*_sigma_ = 0.0295), which were used in all calculations. The final *R*_1_ was 0.0437 (*I* > 2*σ*(*I*)) and *wR*_2_ was 0.1218. Flack parameter: 0.05(4). Crystallographic data for **1** were deposited at the Cambridge Crystallographic Data Centre as deposit no. CCDC 2203317.

*Crystal data for Compound***2**. The crystal was kept at 220 K during data collection. C_32_H_42_O_10_: *M* = 586.65 g/mol, orthorhombic, space group *P*2_1_2_1_2_1_ (no. 19), *a* = 11.1641(4) Å, *b* = 9.3279(3) Å, *c* = 28.8901(12) Å, *α* = 90°, *β* = 90°, *γ* = 90°, *V* = 3008.55(19) Å^3^, *Z* = 4, *T* = 220 K, *μ*(Cu K*α*) = 0.790 mm^−1^, *F* = 1256.0, *D*_calc_ = 1.295 g/cm^3^, 30,900 reflections measured (6.118° ≤ 2*σ* ≤ 133.192°), 5130 unique (*R*_int_ = 0.0850, *R*_sigma_ = 0.0555), which were used in all calculations. The final *R*_1_ was 0.0469 (*I* > 2*σ*(*I*)) and *wR*_2_ was 0.1239. Flack parameter: -−0.03(13). Crystallographic data for **2** were deposited at the Cambridge Crystallographic Data Centre as deposit no. CCDC 2203323.

*Crystal data for Compound***4**. The crystal was kept at 170 K during data collection. C_34_H_49_NO_12_: *M* = 663.74 g/mol, orthorhombic, space group *P*2_1_2_1_2_1_ (no. 19), *a* = 9.0286(2) Å, *b* = 12.9608(3) Å, *c* = 27.8297(6) Å, *α* = 90°, *β* = 90°, *γ* = 90°, *V* = 3256.57(13) Å^3^, *Z* = 4, *T* = 170 K, *μ*(Cu K*α*) = 0.849 mm^−1^, *F* = 1424.0, *D*_calc_ = 1.354 g/cm^3^, 25,652 reflections measured (6.352° ≤ 2*σ* ≤ 149.212°), 6332 unique (*R*_int_ = 0.0392, *R*_sigma_ = 0.0322), which were used in all calculations. The final *R*_1_ was 0.0363 (*I* > 2*σ*(*I*)) and *wR*_2_ was 0.1051. Flack parameter: 0.06(6). Crystallographic data for **4** were deposited at the Cambridge Crystallographic Data Centre as deposit no. CCDC 2203333.

*Crystal data for Compound***5**. The crystal was kept at 140 K during data collection. C_32_H_47_ClO_11_: *M* = 643.14 g/mol, monoclinic, space group *P*2_1_ (no. 4), *a* = 7.5473(5) Å, *b* = 13.8320(9) Å, *c* = 15.5074(10) Å, *α* = 90°, *β* = 92.590(5)°, *γ* = 90°, *V* = 1617.23(18) Å^3^, *Z* = 2, *T* = 140 K, *μ*(Cu K*α*) = 1.545 mm^−1^, *F* = 688.0, *D*_calc_ = 1.321 g/cm^3^, 9921 reflections measured (5.704° ≤ 2*σ* ≤ 127.678°), 4748 unique (*R*_int_ = 0.0635, *R*_sigma_ = 0.0889), which were used in all calculations. The final *R*_1_ was 0.0725 (*I* > 2*σ*(*I*)) and *wR*_2_ was 0.2176. Flack parameter: 0.12(2). Crystallographic data for **5** were deposited at the Cambridge Crystallographic Data Centre as deposit no. CCDC 2203335.

*Crystal data for Compound***9**. The crystal was kept at 150 K during data collection. C_60_H_96_N_2_O_13_: *M* = 1053.38 g/mol, monoclinic, space group C2 (no. 5), *a* = 31.1775(11) Å, *b* = 10.9614(4) Å, *c* = 21.7690(7) Å, *α* = 90°, *β* = 129.3350(10)°, *γ* = 90°, *V* = 5754.1(4) Å^3^, *Z* = 4, *T* = 150 K, *μ*(Cu K*α*) = 0.678 mm^−1^, *F* = 2296.0, *D*_calc_ = 1.216 g/cm^3^, 40,634 reflections measured (5.248° ≤ 2*σ* ≤ 149.634°), 11,598 unique (*R*_int_ = 0.0410, *R*_sigma_ = 0.0375), which were used in all calculations. The final *R*_1_ was 0.0498 (*I* > 2*σ*(*I*)) and *wR*_2_ was 0.1418. Flack parameter: 0.01(4). Crystallographic data for **9** were deposited at the Cambridge Crystallographic Data Centre as deposit no. CCDC 2203338.

*Crystal data for Compound***10**. The crystal was kept at 170 K during data collection. C_28_H_54_O_12_: *M* = 582.71 g/mol, monoclinic, space group P21 (no. 4), *a* = 14.1483(13) Å, *b* = 5.9754(5) Å, *c* = 19.3151(16) Å, *α* = 90°, *β* = 109.720(5)°, *γ* = 90°, *V* = 1537.2(2) Å^3^, *Z* = 2, *T* = 170 K, *μ*(Cu K*α*) = 0.807 mm^−1^, *F* = 636.0, *D*_calc_ = 1.259 g/cm^3^, 23,412 reflections measured (4.86° ≤ 2*σ* ≤ 149.72°), 6167 unique (*R*_int_ = 0.0823, *R*_sigma_ = 0.0794), which were used in all calculations. The final *R*_1_ was 0.0761 (*I* > 2*σ*(*I*)) and *wR*_2_ was 0.2051. Flack parameter: 0.05(15). Crystallographic data for **10** were deposited at the Cambridge Crystallographic Data Centre as deposit no. CCDC 2203339.

*Crystal data for Compound***11**. The crystal was kept at 220 K during data collection. C_28_H_44_O_8_: *M* = 508.63 g/mol, monoclinic, space group P21 (no. 4), *a* = 13.7016(5) Å, *b* = 6.5627(2) Å, *c* = 14.6317(5) Å, *α* = 90°, *β* = 90.629(2)°, *γ* = 90°, *V* = 1315.60(8) Å^3^, *Z* = 2, *T* = 220 K, *μ*(Cu K*α*) = 0.757 mm^−1^, *F* = 552.0, *D*_calc_ = 1.284 g/cm^3^, 19,688 reflections measured (6.04° ≤ 2*σ* ≤ 141.098°), 4713 unique (*R*_int_ = 0.0448, *R*_sigma_ = 0.0355), which were used in all calculations. The final *R*_1_ was 0.0360 (*I* > 2*σ*(*I*)) and *wR*_2_ was 0.1041. Flack parameter: −0.06(12). Crystallographic data for **11** were deposited at the Cambridge Crystallographic Data Centre as deposit no. CCDC 2203341.

### 3.5. Cell Culture

The murine hepatic AML12 cells were purchased from American Type Culture Collection (Rockville, MD, USA) and cultured in DMEM supplemented with 10% fetal bovine serum (Thermo, Rockford, IL, USA), 5 mg/mL insulin, 5 mg/L transferrin, and 5 μg/L selenous acid (Peiyuan, Shanghai, China) in a humidified incubator containing 5% CO_2_ at 37 °C [41].

### 3.6. Cell Viability

The cell viability was evaluated by a colorimetric MTT method as described in our previous report [41]. AML12 cells (1 × 10^4^ cells/well) were seeded into 96-well plates and cultured for 24 h. Then, the cells were treated with *t*-BHP (100 μM) and with or without compounds **1**–**17** at different concentrations for 12 h. Then, medium with 1 mg/mL MTT was added into each well. After incubating for 4 h, 150 μL DMSO was added to solubilize formazan precipitates. Finally, the absorptions at 570 nm were measured using a microplate reader (FlexStation 3, Molecular Devices, CA, USA). DMSO was used as a blank control, and resveratrol (Res) at 10 μM was used as a positive control.

### 3.7. Intracellular ROS Determination

Intracellular ROS levels were detected using 2,7-dichlorodihydrofluorescein diacetate (DCFH-DA, Invitrogen, Carlsbad, CA, USA) as previously described [42]. In brief, AML12 cells (1 × 10^4^ cells/well) were seeded into each well of 96-well plates and cultured for 24 h. The cells were treated with *t*-BHP (100 μM) and different concentrations of compound **8** (0, 2.5, 5, and 10 μM) for 12 h, and then incubated with DCFH-DA (10 μM) at 37 °C in the dark for 20 min. The fluorescence intensity was measured using the microplate reader with excitation and emission wavelength at 488 and 525 nm, respectively.

### 3.8. Determination of GSH Level

The AML12 cells were treated with *t*-BHP (100 μM) and different concentrations of compound **8** (0, 2.5, 5, and 10 μM) for 12 h. The levels of GSH were determined using a commercial assay kit (Nanjing Jiancheng, Nanjing, Jiangsu, China) in accordance with the manufacturer’s protocols. Protein concentration was determined by a BCA Protein Assay Kit (Pierce, Rockford, IL, USA). GSH levels were normalized by total protein content.

### 3.9. Western Blot Analysis

The AML12 cells were treated with *t*-BHP (100 μM) and different concentrations of compound **8** (0, 2.5, 5, and 10 μM) for 12 h. After washed in pre-cooling PBS, the cells were lysed in RIPA buffer (Beyotime, Shanghai, China) containing EDTA and protein phosphatase inhibitors. Equal amounts of protein (20 µg) were separated via SDS–PAGE and transferred onto PVDF membranes, where they were stained with antibodies specific to the target proteins [anti-β-actin, anti-Keap-1, anti-Nrf2, and anti-HO-1 (Cell Signaling, Danvers, MA, USA)] overnight after being blocked for 2 h in TBST containing 5% non-fat dried milk. Then, the membranes were probed with an HRP-conjugated secondary antibody for 1 h at room temperature after washed using TBST. The bands were visualized using the ChemiDoc MP Imaging System (Bio-Rad, Hercules, CA, USA).

### 3.10. Confocal Immunofluorescence

AML12 cells were treated with *t*-BHP (100 μM) and with or without compound **8** (10 μM) for 12 h. The cells were blocked with 5% BSA after fixed with 4% paraformaldehyde. Then, the specific primary antibody (1:100 dilution) was added and incubated at 4 °C overnight and then washed twice with 0.1% triton buffer, followed by incubating with the Texas Red-conjugated secondary antibody (1:1000 dilution, Invitrogen) for 2 h at room temperature. The fluorescent images were captured by a confocal microscope (Olympus, Tokyo, Japan).

### 3.11. Nucleus Isolation

Nuclear proteins were extracted from AML12 cells using a Nuclear Protein Extract Kit (Beyotime). In brief, the AML12 cells were treated with *t*-BHP (100 μM) and different concentrations of compound **8** (0, 2.5, 5, and 10 μM) for 12 h. The cells were re-suspended in 200 μL buffer A and then vortexed for 5 s. After incubation on ice for 15 min, 10 μL buffer B was added and then vortexed for 5 s. The supernatant was discarded after centrifugation at 16,000× *g* for 5 min at 4 °C. The pellet was re-suspended in 50 μL nuclear extraction buffer and then vortexed for 30 s. The nuclei proteins were collected in the supernatant by centrifugation at 16,000× *g* for 5 min at 4 °C.

### 3.12. Statistical Analysis

All experiments were performed with at least three biological replicates. Data are presented as the mean ± standard deviation (S.D.) and the significant differences among multiple groups were analyzed with one-way ANOVA, followed by Tukey’s post-hoc test. Figures were prepared using GraphPad Prism software Version 7.0 (GraphPad Software, Inc., San Diego, CA, USA). The analyses were conducted considering *p* < 0.05 as a significant difference in all comparisons.

## Figures and Tables

**Table 1:** ^1^H and ^13^C NMR data for compounds **1**–**4** (*δ* in ppm, *J* in Hz, in pyridine-*d_5_*).

Position	1 *^a^*		2 *^b^*		3 *^b^*		4 *^b^*	
	*δ*_C_	*δ*_H_ (*J* in Hz)	*δ*_C_	*δ*_H_ (*J* in Hz)	*δ*_C_	*δ*_H_ (*J* in Hz)	*δ*_C_	*δ*_H_ (*J* in Hz)
1	72.9	4.89, d (5.7)	72.8	4.91, d (5.3)	70.4	3.83, m	71.9	4.60, d (5.2)
2	52.0	3.87, m	52.0	3.89, dd (5.2, 3.7)	53.7	3.53, dd (5.6, 3.7)	51.2	3.71, dd (5.2, 3.6)
3	55.9	3.54, dd (3.7, 1.9)	56.0	3.56, dt (3.7, 1.7)	54.1	3.46, t (2.9)	55.2	3.54, dd (3.6, 1.8)
4	33.6	*α* 2.36, m *β* 2.08, m	33.6	*α* 2.38, dd (15.6, 1.5) *β* 2.08, dd (15.6, 2.1)	34.7	*α* 2.46, m *β* 1.99, dd (15.6, 2.5)	32.7	*α* 2.38, d (15.6) *β* 2.03, m
5	70.7		70.7		71.8		70.1	
6	57.0	2.95, d (3.5)	57.1	2.98, d (3.6)	57.9	2.94, d (3.7)	56.3	2.83, d (3.6)
7	54.6	3.11, t (2.6)	54.4	3.09, dd (3.6, 2.0)	56.3	3.24, dd (3.7, 2.1)	54.3	3.10, dd (3.6, 2.0)
8	36.8	1.72, m	35.8	1.96, td (11.2, 2.2)	36.7	1.79, m	36.1	1.73, td (11.0, 2.0)
9	28.9	2.42, m	29.2	2.56, m	29.2	2.47, m	28.2	2.02, m
10	40.8		41.0		40.4		40.0	
11	25.2	*α* 1.79, m *β* 1.46, td (14.0, 2.4)	24.7	*α* 1.85, dt (14.4, 3.4) *β* 1.59, td (14.2, 2.5)	25.8	*α* 2.45, m *β* 1.58, m	24.9	1.50, m 1.46, m
12	76.2	5.09, t (3.4)	74.9	5.14, q (3.3)	75.9	5.29, d (3.0)	75.4	4.97, d (2.7)
13	46.6		46.9		46.7		46.2	
14	45.4	2.12, m	40.9	2.62, m	45.8	2.14, m	44.6	2.02, m
15	23.3	1.73, m 1.19, m	37.9	2.51, m 2.15, m	23.4	1.80, m 1.27, m	22.9	1.87, m 1.30, m
16	26.9	1.22, m	216.1		27.1	1.55, m 1.23, m	27.6	1.99, m 1.40, m
17	44.2	1.80, m	56.7	2.67, d (8.7)	44.2	1.78, m	43.9	2.16, m
18	12.5	0.70, s	14.5	0.96, s	12.6	0.73, s	12.0	0.76, s
19	16.7	0.77, s	16.7	0.81, s	15.7	0.69, s	16.4	0.76, s
20	39.0	2.04, m	35.6	2.47, m	39.1	1.92, m	39.5	1.94, m
21	12.3	1.06, d (6.3)	13.2	1.01, d (7.1)	12.9	1.03, d (6.8)	13.6	0.89, d (6.7)
22	79.7	5.11, m	77.7	5.16, m	78.7	4.40, dt (13.2, 3.5)	83.5	4.29, m
23	33.5	2.20, m 2.00, m	31.6	2.25, m 2.14, m	30.1	2.38, dd (17.6, 13.0); 2.05, m	66.7	4.27, m
24	73.6		149.6		154.3		156.2	
25	77.5		122.4		127.9		125.3	
26	179.2		166.7		166.7		165.6	
27	23.7	1.62, s	13.1	1.89, s	56.6	4.87, d (11.7) 4.77, d (11.7)	57.7	4.34, m
28	25.4	1.62, s	20.5	1.74, s	20.5	2.11, s	15.4	2.08, s
1-OAc	20.8 170.7	2.13, s	20.8 170.8	2.16, s			20.4 170.3	2.00, s
12-OAc	21.5 170.8	2.04, s	21.7 170.8	2.18, s	21.4 170.5	1.96, s	21.5 170.5	2.06, s
5-OH								3.45

*^a^* Recorded at 500 MHz (^1^H) and 125 MHz (^13^C). *^b^* Recorded at 600 MHz (^1^H) and 125 MHz (^13^C).

**Table 2:** ^1^H and ^13^C NMR data for compounds **5**–**8** (*δ* in ppm, *J* in Hz).

Position	5 *^a^*		6 *^a^*		7 *^a^*		8 *^b^*	
	*δ*_C_	*δ*_H_ (*J* in Hz)	*δ*_C_	*δ*_H_ (*J* in Hz)	*δ*_C_	*δ*_H_ (*J* in Hz)	*δ*_C_	*δ*_H_ (*J* in Hz)
1	74.2	4.09, dd (10.0, 3.9)	74.2	4.11, dd (9.9, 4.0)	74.0	4.08, d (3.9)	73.8	5.11, d (4.3)
2	76.9	5.73, dd (10.9, 3.9)	76.9	5.74, dd (11.0, 4.0)	76.9	5.70, dd (10.9, 3.9)	72.8	4.16, dd (10.4, 4.3)
3	57.2	5.12, td (11.5, 5.5)	57.3	5.12, td (11.5, 5.4)	57.2	5.10, m	58.9	4.48, td (11.9, 5.0)
4	44.1	*α* 2.57, dd (13.3, 5.5) *β* 2.40, dd (14.6, 10.6)	44.1	*α* 2.58, m *β* 2.41, dd (13.3, 12.0)	44.0	*α* 2.55, m *β* 2.39, dd (13.3, 11.9)	42.2	*α* 2.37, m *β* 2.15, m
5	74.5		74.5		74.5		71.0	
6	56.9	3.04, d (3.7)	57.0	3.05, d (3.7)	56.9	3.04, d (3.7)	57.5	3.05, d (3.7)
7	56.0	3.27, t (3.0)	56.0	3.31, t (3.1)	55.7	3.24, t (3.0)	56.5	3.26, t (3.0)
8	36.1	1.82, m	35.9	2.01, m	35.1	2.06, dd (11.1, 2.4)	34.8	2.03, m
9	30.1	2.51, ddd (14.1, 11.3, 3.5)	30.4	2.54, m	30.3	2.68, m	29.6	1.98, dd (9.9, 6.4)
10	41.8		41.8		41.9		41.9	
11	25.7	*α* 2.21, dt (14.2, 3.3) *β* 1.45, td (14.0, 2.8)	25.4	*α* 2.25, m *β* 1.56, td (13.8, 2.7)	25.2	*α* 2.27, m *β* 1.58, td (13.9, 2.6)	24.3	1.60, m
12	75.7	5.25, m	76.1	5.32, m	74.3	5.31, m	74.5	5.04, d (2.9)
13	46.9		47.0		47.3		46.8	
14	45.8	2.11, m	44.3	2.13, m	41.1	2.60, m	40.0	2.41, m
15	23.3	*α* 1.83, m *β* 1.31, dd (12.3, 5.6)	37.1	*α* 2.63, dt (12.7, 7.6) *β* 1.79, m	38.0	2.55, m 2.25, m	37.3	2.50, m 2.07, m
16	27.1	1.55, dtd (13.1, 9.5, 5.5) 1.24, m	70.1	4.40, tt (8.0, 4.3)	216.1		214.5	
17	44.2	1.70, m	49.3	1.81, m	56.9	2.68, m	56.6	2.53, m
18	12.6	0.73, s	14.1	1.23, s	14.8	0.99, s	14.9	1.01, s
19	15.3	0.91, s	15.8	0.93, s	15.9	0.92, s	16.5	1.00, s
20	39.1	1.93, m	33.9	2.94, dtd (10.6, 6.9, 3.4)	35.7	2.47, td (7.8, 5.6)	34.8	2.36, m
21	13.0	1.00, d (6.7)	12.4	1.14, d (7.0)	13.5	1.00, d (7.1)	14.0	0.96, d (7.0)
22	78.7	4.40, dt (13.2, 3.5)	78.4	5.27, dt (13.3, 3.5)	77.8	5.13, m	77.4	4.84, ddd (12.7, 6.2, 3.4)
23	30.2	2.37, m 2.03, dd (18.1, 3.1)	30.8	2.49, dt (12.7, 7.6) 1.79, m	32.2	2.25, m	32.6	2.36, m 2.15, m
24	154.3		154.3		154.2		152.4	
25	127.9		127.9		127.9		126.1	
26	166.7		166.9		166.5		166.5	
27	56.6	4.87, d (11.7) 4.77, d (11.7)	56.7	4.75, m	56.7	4.71, m	57.5	4.34, m
28	20.5	2.11, s	20.5	2.10, s	20.5	2.00, s	20.8	2.03, s
1-OAc							20.1 171.5	2.03, s
2-OAc	21.4 171.0	2.17, s	21.4 171.0	2.17, s	21.4 171.0	2.15, s		
12-OAc	21.3 170.3	1.94, s	21.4 170.3	1.98, s	21.5 170.5	2.03, s	21.4 169.6	2.13, s
1-OH		5.76, d (10.0)		5.71, d (9.9)				
5-OH		7.41, s		7.32, s				3.19, s
15-OH				6.37, d (4.5)				

*^a^* Recorded at 600 MHz (^1^H) and 125 MHz (^13^C) in pyridine-*d_5_*. *^b^* Recorded at 500 MHz (^1^H) and 125 MHz (^13^C) in chloroform-*d*.

**Table 3:** ^1^H and ^13^C NMR data for compounds **9**–**11** (*δ* in ppm, *J* in Hz, in pyridine-*d_5_*).

Position	9 *^a^*		10 *^b^*		11 *^a^*	
	*δ*_C_	*δ*_H_ (*J* in Hz)	*δ*_C_	*δ*_H_ (*J* in Hz)	*δ*_C_	*δ*_H_ (*J* in Hz)
1	78.5	3.83, dd (11.9, 4.2)	71.0	4.54, dt (11.1, 4.7)	210.6	
2	44.2	*α* 2.66, m *β* 2.28, q (11.7)	43.5	*α* 2.81, dtd (12.8, 5.6, 1.6) *β* 2.32, q (11.6)	49.2	*α* 3.04, dd (13.5, 6.8) *β* 3.33, dd (13.5, 9.8)
3	68.4	4.01, tdd (9.6, 7.2, 4.4)	64.7	4.89, m	66.4	5.06, m
4	44.0	2.71, m	43.9	*α* 2.37, m *β* 2.14, m	43.2	2.56, m
5	141.1		72.9		73.7	
6	131.8	5.94, d (1.7)	59.4	3.14, d (3.8)	57.4	3.20, m
7	72.8	4.10, d (8.3)	57.8	3.26, dd (3.8, 2.4)	56.6	3.20, m
8	42.5	1.73, m	37.1	1.91, m	36.5	1.67, m
9	50.2	1.55, m	39.9	1.90, m	36.5	2.07, m
10	43.7		44.3		53.9	
11	24.9	*α* 2.96, dq (14.3, 3.7) *β* 1.74, m	25.0	*α* 2.42, m *β* 1.78, m	22.5	*α* 2.72, dq (13.0, 3.5) *β* 1.27, m
12	41.1	2.01, dt (14.2, 4.3) 1.29, m	41.0	1.97, m 1.13, m	40.7	1.91, m 1.14, m
13	43.7		44.0		44.2	
14	57.2	1.26, m	51.9	1.34, m	52.0	1.28, m
15	28.0	*α* 2.23, dt (9.3, 3.1) *β* 1.80, m	24.2	*α* 1.69, m *β* 1.19, m	24.1	*α* 1.64, m *β* 1.17 m
16	28.2	1.80, m 1.32, m	27.7	*α* 1.66, m *β* 1.25, m	27.7	1.64, m 1.21, m
17	52.4	1.14, m	52.6	1.06, m	52.5	0.99, m
18	12.6	0.74, s	12.5	0.67, s	12.7	0.64, s
19	14.0	1.34, s	12.1	1.17, s	16.8	1.30, s
20	40.1	2.08, ddt (9.3, 6.5, 3.1)	40.0	2.04, ddt (9.8, 7.0, 3.6)	39.9	2.01, m
21	13.6	1.06, d (6.6)	13.4	1.00, d (6.6)	13.3	0.97, d (6.6)
22	79.1	5.13, m	78.9	5.09, m	78.8	5.09, m
23	37.1	1.98, m 1.90, m	37.1	1.96, m 1.89, m	37.0	1.88, m
24	69.9		69.8		69.8	
25	47.6	2.58, q (7.0)	47.5	2.57, q (7.0)	47.5	2.56, m
26	175.1		175.0		175.0	
27	10.6	1.65, d (7.0)	10.6	1.64, d (7.0)	10.6	1.64, d (7.0)
28	28.7	1.56, s	28.6	1.56, s	28.6	1.53, s
5-OH				4.69, s		5.93, s

*^a^* Recorded at 600 MHz (^1^H) and 125 MHz (^13^C). *^b^* Recorded at 500 MHz (^1^H) and 125 MHz (^13^C).

## Data Availability

All data generated or analyzed during this study are included in this published article.

## Supplementary Materials

The following supporting information can be downloaded at: https://www.mdpi.com/article/10.3390/molecules27238197/s1, Key ^1^H-^1^H COSY and HMBC correlations of compounds **1**–**4**, **6**–**8**, **10**, and **11**; key NOESY correlations of compounds **1**–**3**; key ROESY correlations of compounds **4**, **6**–**8**, **10**, and **11**; 1D and 2D NMR data, IR spectra, and HRESIMS spectra of new compounds (**1**–**11**); 1D NMR of known compounds (**12**–**17**); Cytotoxicity of compounds **1**–**17** on AML-12 hepatocytes; X-ray crystallographic data (CIFs) for compounds **1**, **2**, **4**, **5**, **9**, **10**, and **11**.

## Sample Availability

Samples of compounds **1**–**17** are available from the authors.
