# Supplementary material for: Chantriolides F–P, Highly Oxidized Withanolides with Hepatoprotective Activity from Tacca chantrieri
Source: Molecules. 2022 Nov 24;27(23):8197. doi: 10.3390/molecules27238197 (PMC9739516; doi:10.3390/molecules27238197)
Supplement: Supplementary file 1 [file molecules-27-08197-s001.zip › molecules-2018616-supplementary.pdf]

Supporting information for

Chantriolides F–P, Highly Oxidized Withanolides with Hepatoprotective Activity from *Tacca chantrieri*

Yue Yang,[1,2] Fei Zhou,[3] Min Wang,[2,4] Turanazarov Mukhammadrizo,[2,4] Xiao-Rong Wang,[5] Changqiang Ke,[1,2] Sheng Yao,[1,2] Ligen Lin,[3] Chunping Tang, [1,2,*] Yang Ye[1,2,4,6,*]

[1] State Key Laboratory of Drug Research, Shanghai Institute of Materia Medica, Chinese Academy of Sciences, Shanghai 201203, China

[2] Natural Products Chemistry Department, Shanghai Institute of Materia Medica, Chinese Academy of Sciences, Shanghai 201203, China

[3] State Key Laboratory of Quality Research in Chinese Medicine, Institute of Chinese Medical Sciences, University of Macau, Avenida da Universidade, Taipa, Macau 999078, China

[4] University of Chinese Academy of Sciences, No. 19A Yuquan Road, Beijing 100049, China

[5] Xishuangbanna Research Institute of Nationality Medicine, Xishuangbanna Hospital of Traditional Dai Medicine, No.8, Zhuangdong Western Road of Xishuangbanna Tourism and Resort Zone, Xishuangbanna 666100, China

[6] School of Life Science and Technology, ShanghaiTech University, 393 Middle Huaxia Road, Pudong, Shanghai 201210, China

***** Correspondence: tangcp@simm.ac.cn (C.T.); yye@simm.ac.cn (Y.Y.)

Contents

Figure S1. Key ^{1}H-^{1}H COSY and HMBC correlations of compounds **1–4**, **6–8**, **10**, and **11**...5
Figure S2. NOESY correlations of compounds **1–3** and ROESY correlations of compounds **4**, **6–8**, **10**, and **11**....5
Figure S3. HRESIMS spectrum of chantriolide F (**1**)....6
Figure S4. IR spectrum of chantriolide F (**1**)....6
Figure S5. ^{1}H NMR (500 MHz) spectrum of chantriolide F (**1**) in C_5D_5N....7
Figure S6. ^{13}C and DEPT-135 NMR (125 MHz) spectra of chantriolide F (**1**) in C_5D_5N....7
Figure S7. HSQC spectrum of chantriolide F (**1**) in C_5D_5N....8
Figure S8. ^{1}H-^{1}H COSY spectrum of chantriolide F (**1**) in C_5D_5N....8
Figure S9. HMBC spectrum of chantriolide F (**1**) in C_5D_5N....9
Figure S10. NOESY spectrum of chantriolide F (**1**) in C_5D_5N....9
Figure S11. HRESIMS spectrum of chantriolide G (**2**)....10
Figure S12. IR spectrum of chantriolide G (**2**)....10
Figure S13. ^{1}H NMR (600 MHz) spectrum of chantriolide G (**2**) in C_5D_5N....11
Figure S14. ^{13}C and DEPT-135 NMR (125 MHz) spectra of chantriolide G (**2**) in C_5D_5N....11
Figure S15. HSQC spectrum of chantriolide G (**2**) in C_5D_5N....12
Figure S16. ^{1}H-^{1}H COSY spectrum of chantriolide G (**2**) in C_5D_5N....12
Figure S17. HMBC spectrum of chantriolide G (**2**) in C_5D_5N....13
Figure S18. NOESY spectrum of chantriolide G (**2**) in C_5D_5N....13
Figure S19. HRESIMS spectrum of chantriolide H (**3**)....14
Figure S20. IR spectrum of chantriolide H (**3**)....14
Figure S21. ^{1}H NMR (600 MHz) spectrum of chantriolide H (**3**) in C_5D_5N....15
Figure S22. ^{13}C and DEPT-135 NMR (125 MHz) spectra of chantriolide H (**3**) in C_5D_5N....15
Figure S23. HSQC spectrum of chantriolide H (**3**) in C_5D_5N....16
Figure S24. ^{1}H-^{1}H COSY spectrum of chantriolide H (**3**) in C_5D_5N....16
Figure S25. HMBC spectrum of chantriolide H (**3**) in C_5D_5N....17
Figure S26. NOESY spectrum of chantriolide H (**3**) in C_5D_5N....17
Figure S27. HRESIMS spectrum of chantriolide I (**4**)....18
Figure S28. IR spectrum of chantriolide I (**4**)....18
Figure S29. ^{1}H NMR (400 MHz) spectrum of chantriolide I (**4**) in $CDCl_3$....19
Figure S30. ^{13}C and DEPT-135 NMR (125 MHz) spectra of chantriolide I (**4**) in $CDCl_3$....19
Figure S31. HSQC spectrum of chantriolide I (**4**) in $CDCl_3$....20
Figure S32. ^{1}H-^{1}H COSY spectrum of chantriolide I (**4**) in $CDCl_3$....20
Figure S33. HMBC spectrum of chantriolide I (**4**) in $CDCl_3$....21
Figure S34. ROESY spectrum of chantriolide I (**4**) in $CDCl_3$....21
Figure S35. HRESIMS spectrum of chantriolide J (**5**)....22
Figure S36. IR spectrum of chantriolide J (**5**)....22
Figure S37. ^{1}H NMR (600 MHz) spectrum of chantriolide J (**5**) in C_5D_5N....23
Figure S38. ^{13}C and DEPT-135 NMR (125 MHz) spectra of chantriolide J (**5**) in C_5D_5N....23
Figure S39. HSQC spectrum of chantriolide J (**5**) in C_5D_5N....24
Figure S40. ^{1}H-^{1}H COSY spectrum of chantriolide J (**5**) in C_5D_5N....24
Figure S41. HMBC spectrum of chantriolide J (**5**) in C_5D_5N....25
Figure S42. ROESY spectrum of chantriolide J (**5**) in C_5D_5N....25

Figure S43. HRESIMS spectrum of chantriolide K (**6**) 26
Figure S44. IR spectrum of chantriolide K (**6**) 26
Figure S45. ^{1}H NMR (600 MHz) spectrum of chantriolide K (**6**) in C_5D_5N 27
Figure S46. ^{13}C and DEPT-135 NMR (125 MHz) spectra of chantriolide K (**6**) in C_5D_5N ... 27
Figure S47. HSQC spectrum of chantriolide K (**6**) in C_5D_5N 28
Figure S48. ^{1}H-^{1}H COSY spectrum of chantriolide K (**6**) in C_5D_5N 28
Figure S49. HMBC spectrum of chantriolide K (**6**) in C_5D_5N 29
Figure S50. ROESY spectrum of chantriolide K (**6**) in C_5D_5N 29
Figure S51. HRESIMS spectrum of chantriolide L (**7**) 30
Figure S52. IR spectrum of chantriolide L (**7**) 30
Figure S53. ^{1}H NMR (600 MHz) spectrum of chantriolide L (**7**) in C_5D_5N 31
Figure S54. ^{13}C and DEPT-135 NMR (125 MHz) spectra of chantriolide L (**7**) in C_5D_5N 31
Figure S55. HSQC spectrum of chantriolide L (**7**) in C_5D_5N 32
Figure S56. ^{1}H-^{1}H COSY spectrum of chantriolide L (**7**) in C_5D_5N 32
Figure S57. HMBC spectrum of chantriolide L (**7**) in C_5D_5N 33
Figure S58. ROESY spectrum of chantriolide L (**7**) in C_5D_5N 33
Figure S59. HRESIMS spectrum of chantriolide M (**8**) 34
Figure S60. IR spectrum of chantriolide M (**8**) 34
Figure S61. ^{1}H NMR (500 MHz) spectrum of chantriolide M (**8**) in $CDCl_3$ 35
Figure S62. ^{13}C and DEPT-135 NMR (125 MHz) spectra of chantriolide M (**8**) in $CDCl_3$ ... 35
Figure S63. HSQC spectrum of chantriolide M (**8**) in $CDCl_3$ 36
Figure S64. ^{1}H-^{1}H COSY spectrum of chantriolide M (**8**) in $CDCl_3$ 36
Figure S65. HMBC spectrum of chantriolide M (**8**) in $CDCl_3$ 37
Figure S66. ROESY spectrum of chantriolide M (**8**) in $CDCl_3$ 37
Figure S67. HRESIMS spectrum of chantriolide N (**9**) 38
Figure S68. IR spectrum of chantriolide N (**9**) 38
Figure S69. ^{1}H NMR (600 MHz) spectrum of chantriolide N (**9**) in C_5D_5N 39
Figure S70. ^{13}C and DEPT-135 NMR (125 MHz) spectra of chantriolide N (**9**) in C_5D_5N ... 39
Figure S71. HSQC spectrum of chantriolide N (**9**) in C_5D_5N 40
Figure S72. ^{1}H-^{1}H COSY spectrum of chantriolide N (**9**) in C_5D_5N 40
Figure S73. HMBC spectrum of chantriolide N (**9**) in C_5D_5N 41
Figure S74. ROESY spectrum of chantriolide N (**9**) in C_5D_5N 41
Figure S75. HRESIMS spectrum of chantriolide O (**10**) 42
Figure S76. IR spectrum of chantriolide O (**10**) 42
Figure S77. ^{1}H NMR (500 MHz) spectrum of chantriolide O (**10**) in C_5D_5N 43
Figure S78. ^{13}C and DEPT-135 NMR (125 MHz) spectra of chantriolide O (**10**) in C_5D_5N . 43
Figure S79. HSQC spectrum of chantriolide O (**10**) in C_5D_5N 44
Figure S80. ^{1}H-^{1}H COSY spectrum of chantriolide O (**10**) in C_5D_5N 44
Figure S81. HMBC spectrum of chantriolide O (**10**) in C_5D_5N 45
Figure S82. ROESY spectrum of chantriolide O (**10**) in C_5D_5N 45
Figure S83. HRESIMS spectrum of chantriolide P (**11**) 46
Figure S84. IR spectrum of chantriolide P (**11**) 46
Figure S85. ^{1}H NMR (600 MHz) spectrum of chantriolide P (**11**) in C_5D_5N 47
Figure S86. ^{13}C and DEPT-135 NMR (125 MHz) spectra of chantriolide P (**11**) in C_5D_5N .. 47

Figure S87. HSQC spectrum of chantriolide P (**11**) in C_5D_5N 48
Figure S88. ^1H-^1H COSY spectrum of chantriolide P (**11**) in C_5D_5N 48
Figure S89. HMBC spectrum of chantriolide P (**11**) in C_5D_5N 49
Figure S90. ROESY spectrum of chantriolide P (**11**) in C_5D_5N 49
Figure S91. ^1H NMR (500 MHz) spectrum of plantagiolide E (**12**) in C_5D_5N 50
Figure S92. ^{13}C and DEPT-135 NMR (125 MHz) spectra of plantagiolide E (**12**) in C_5D_5N 50
Figure S93. ^1H NMR (500 MHz) spectrum of plantagiolide C (**13**) in C_5D_5N 51
Figure S94. ^{13}C and DEPT-135 NMR (125 MHz) spectra of plantagiolide C (**13**) in C_5D_5N 51
Figure S95. ^1H NMR (400 MHz) spectrum of plantagiolide B (**14**) in DMSO-d_6 52
Figure S96. ^{13}C and DEPT-135 NMR (125 MHz) spectra of plantagiolide B (**14**) DMSO-d_6 52
Figure S97. ^1H NMR (400 MHz) spectrum of plantagiolide A (**15**) in C_5D_5N 53
Figure S98. ^{13}C and DEPT-135 NMR (125 MHz) spectra of plantagiolide A (**15**) in C_5D_5N 53
Figure S99. ^1H NMR (500 MHz) spectrum of plantagiolide M (**16**) in CD_3OD 54
Figure S100. ^{13}C and DEPT-135 NMR (125 MHz) spectra of plantagiolide M (**16**) in CD_3OD 54
Figure S101. ^1H NMR (400 MHz) spectrum of chantriolide D (**17**) in C_5D_5N 55
Figure S102. ^{13}C and DEPT-135 NMR (125 MHz) spectra of chantriolide D (**17**) in C_5D_5N55
Figure S103. Cytotoxicity of compounds **1–17** on AML-12 hepatocytes. AML-12 cells were treated with different compounds at indicated concentrations for 24 hours. Data are shown as mean ± S.D., n = 3. *$P < 0.05$ and **$P < 0.01$ vs. DMSO. 56

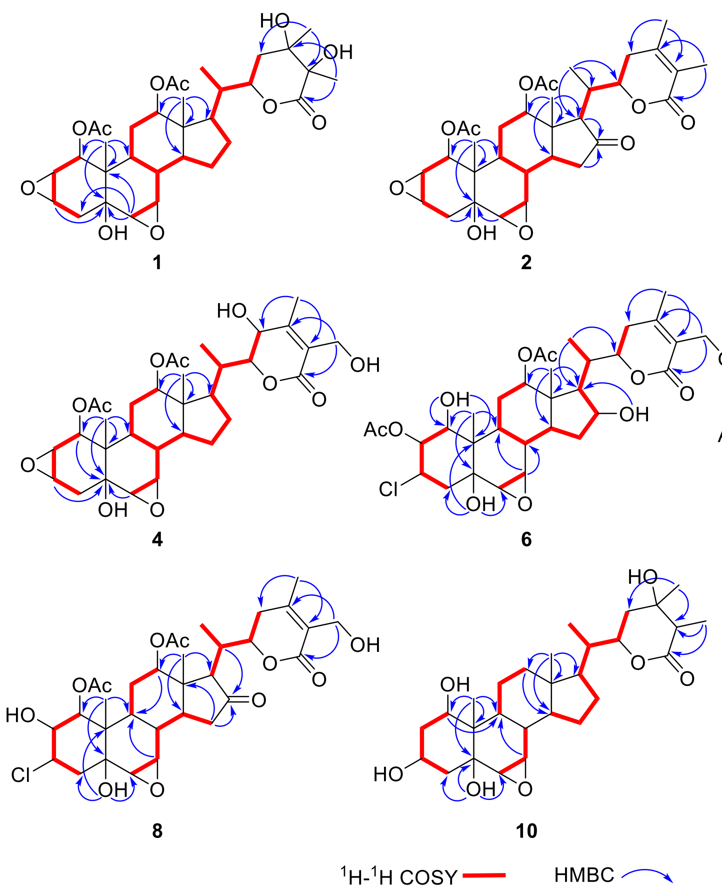

Figure S1. Key ^{1}H-^{1}H COSY and HMBC correlations of compounds **1**–**4**, **6**–**8**, **10**, and **11**.

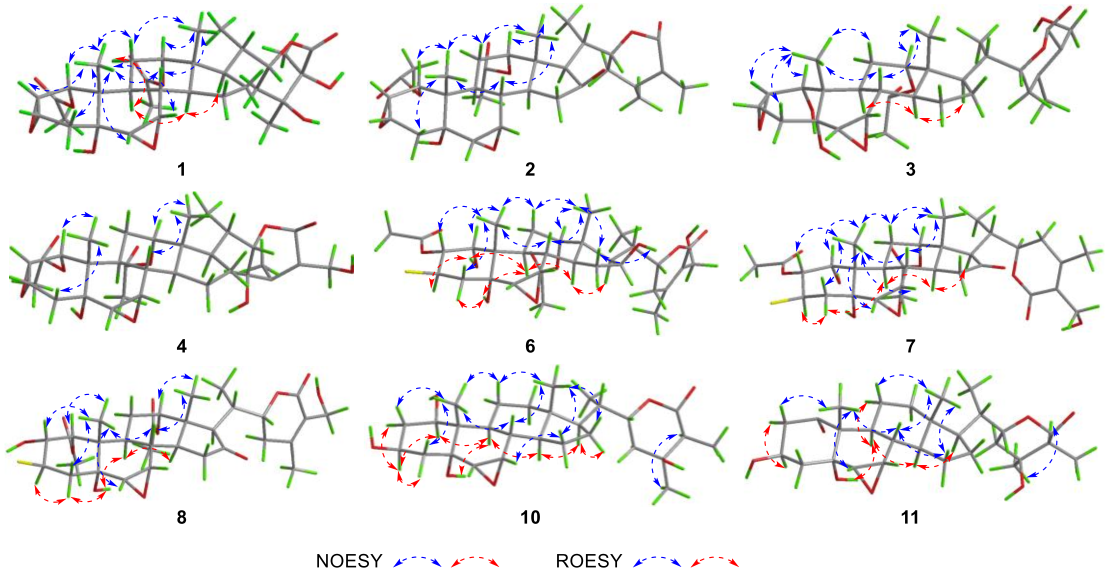

Figure S2. NOESY correlations of compounds **1**–**3** and ROESY correlations of compounds **4**, **6**–**8**, **10**, and **11**.

Elemental Composition Report

Single Mass Analysis

Tolerance = 10.0 PPM / DBE: min = -1.5, max = 50.0

Element prediction: Off

Number of isotope peaks used for i-FIT = 3

Monoisotopic Mass, Even Electron Ions

104 formula(e) evaluated with 3 results within limits (up to 10 best isotopic matches for each mass)

Elements Used:

C: 0-50 H: 0-60 O: 0-20

Minimum:				-1.5				
Maximum:		5.0	10.0	50.0				
Mass	Calc. Mass	mDa	PPM	DBE	i-FIT	Norm	Conf(%)	Formula
651.3023	651.3017	0.6	0.9	10.5	592.1	0.232	79.31	C33 H47 O13

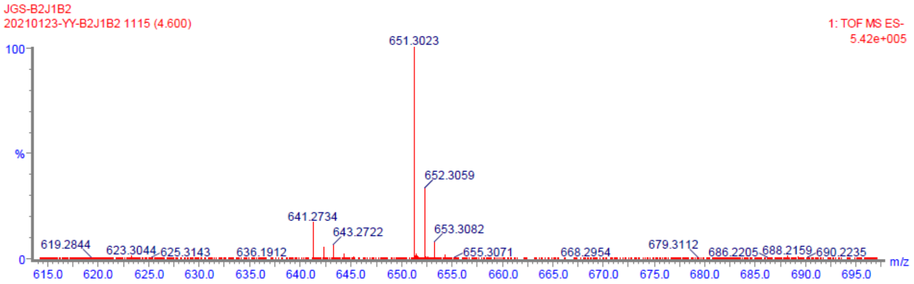

Figure S3. HRESIMS spectrum of chantriolide F (**1**)

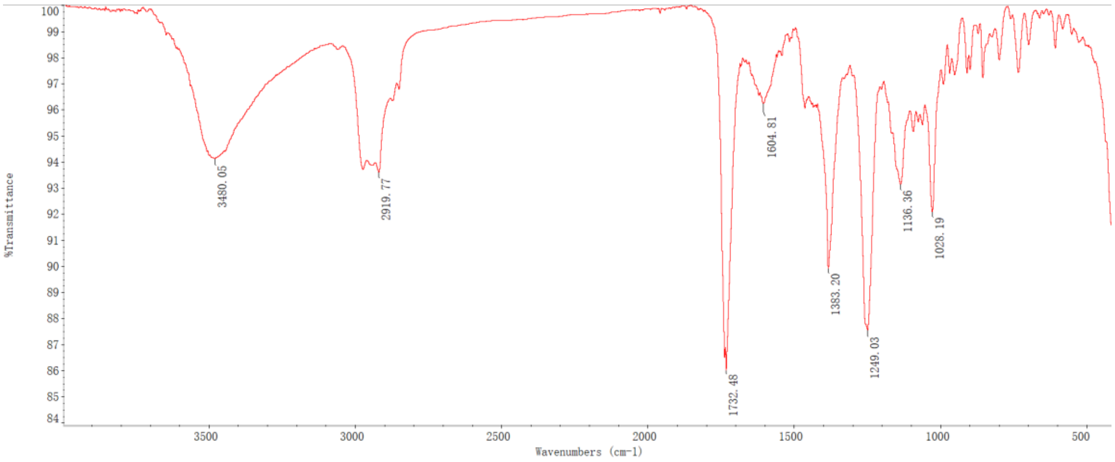

Figure S4. IR spectrum of chantriolide F (**1**)

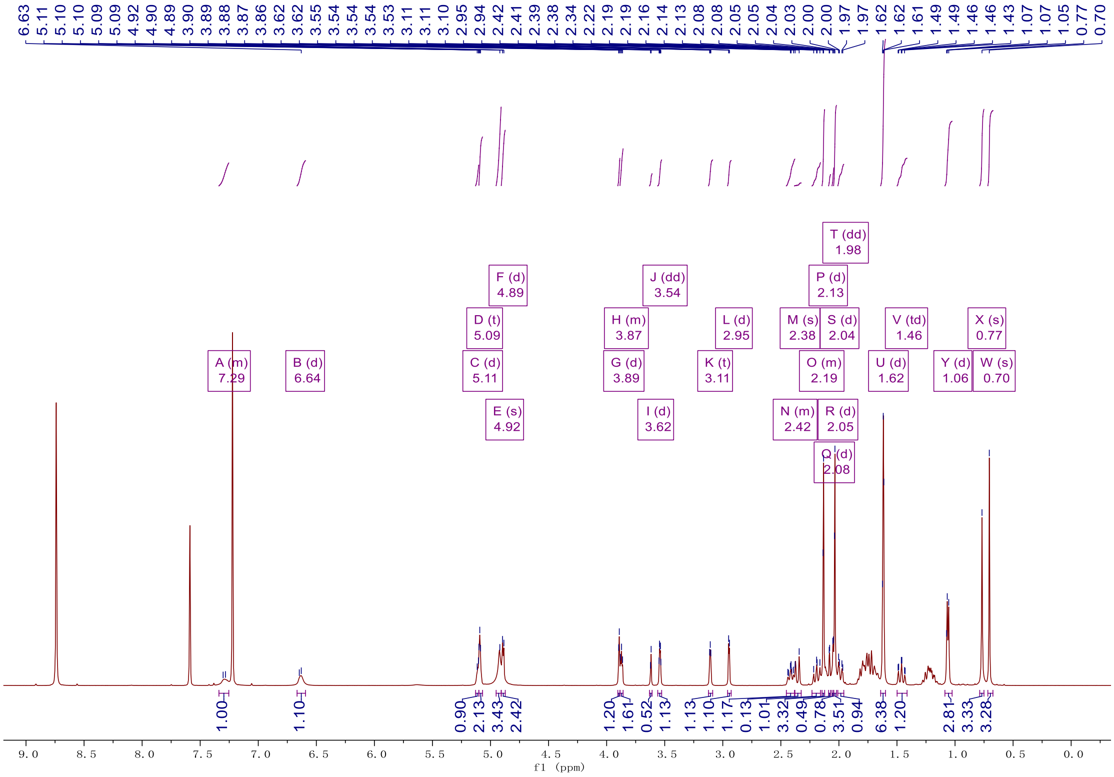

Figure S5. ^{1}H NMR (500 MHz) spectrum of chantriolide F (**1**) in C_5D_5N

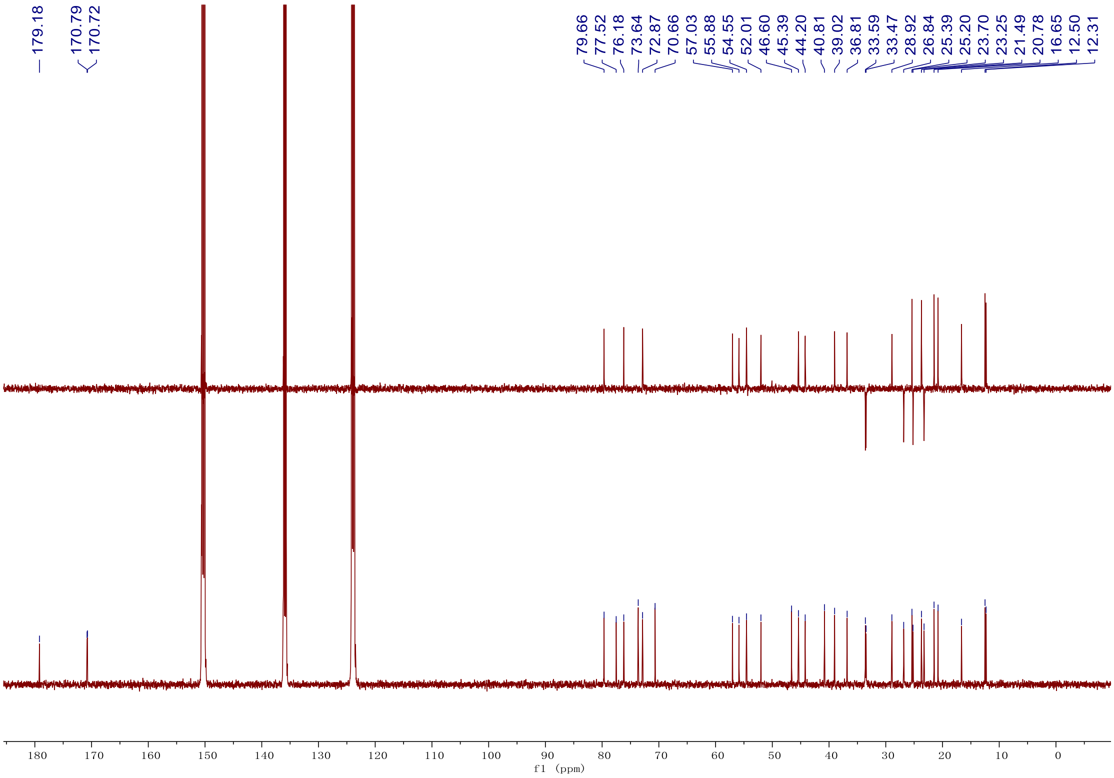

Figure S6. ^{13}C and DEPT-135 NMR (125 MHz) spectra of chantriolide F (**1**) in C_5D_5N

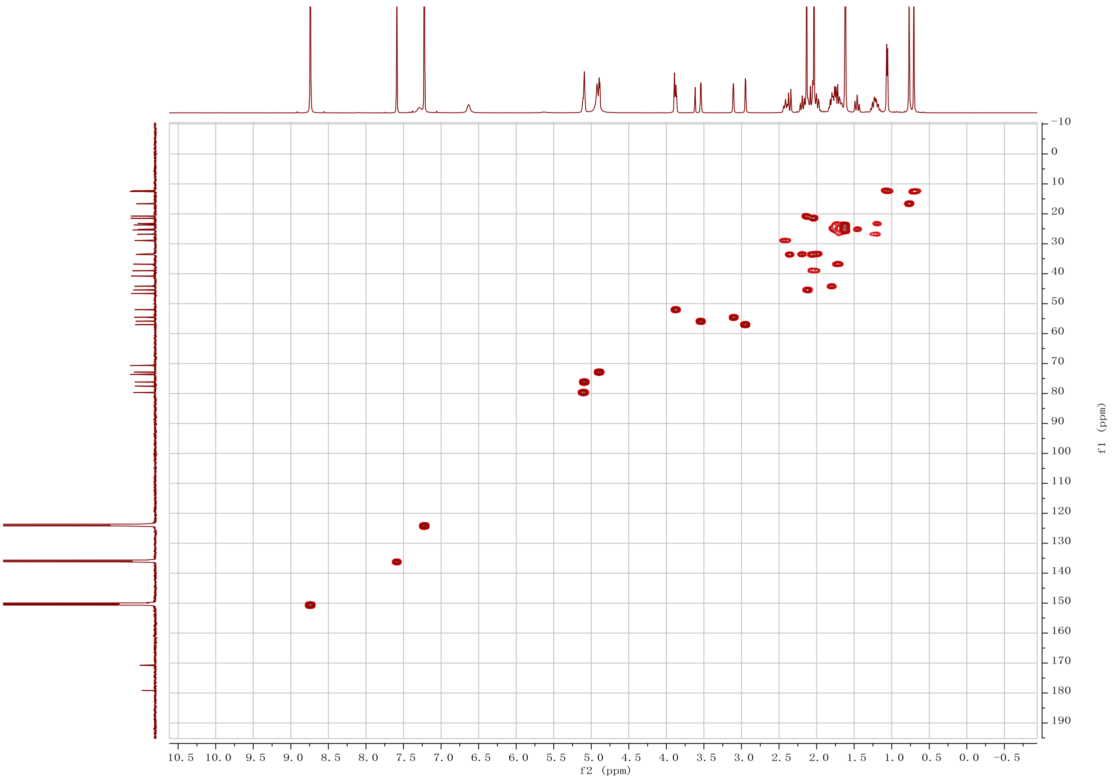

Figure S7. HSQC spectrum of chantriolide F (**1**) in C_5D_5N

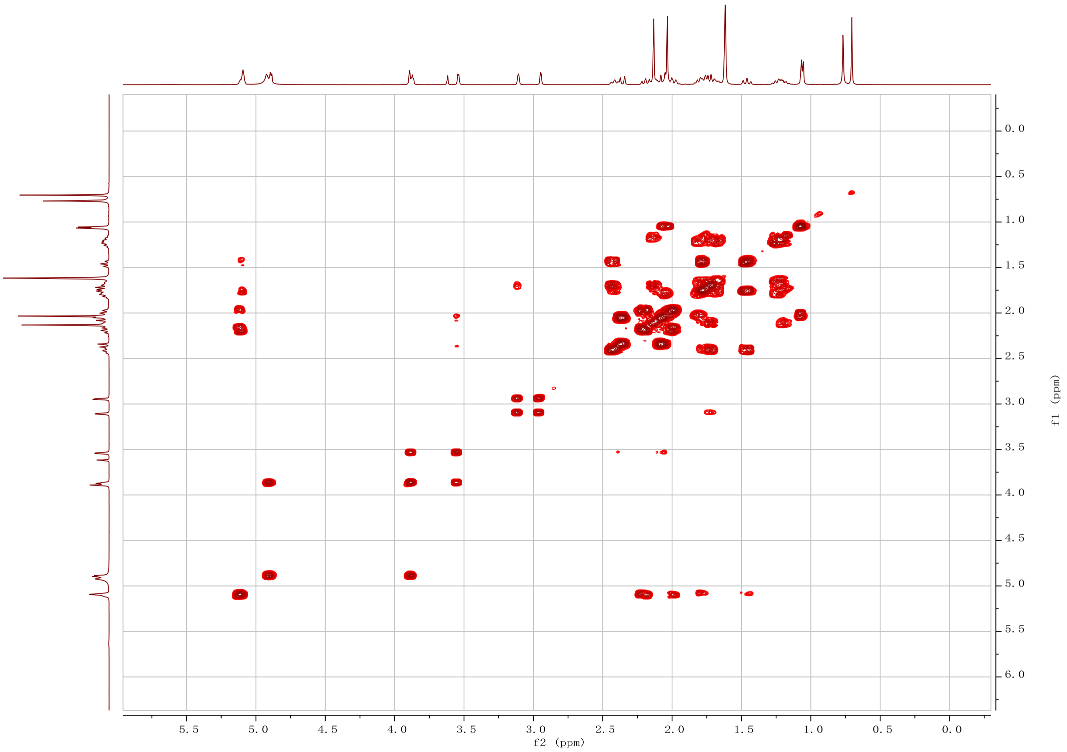

Figure S8. ^{1}H-^{1}H COSY spectrum of chantriolide F (**1**) in C_5D_5N

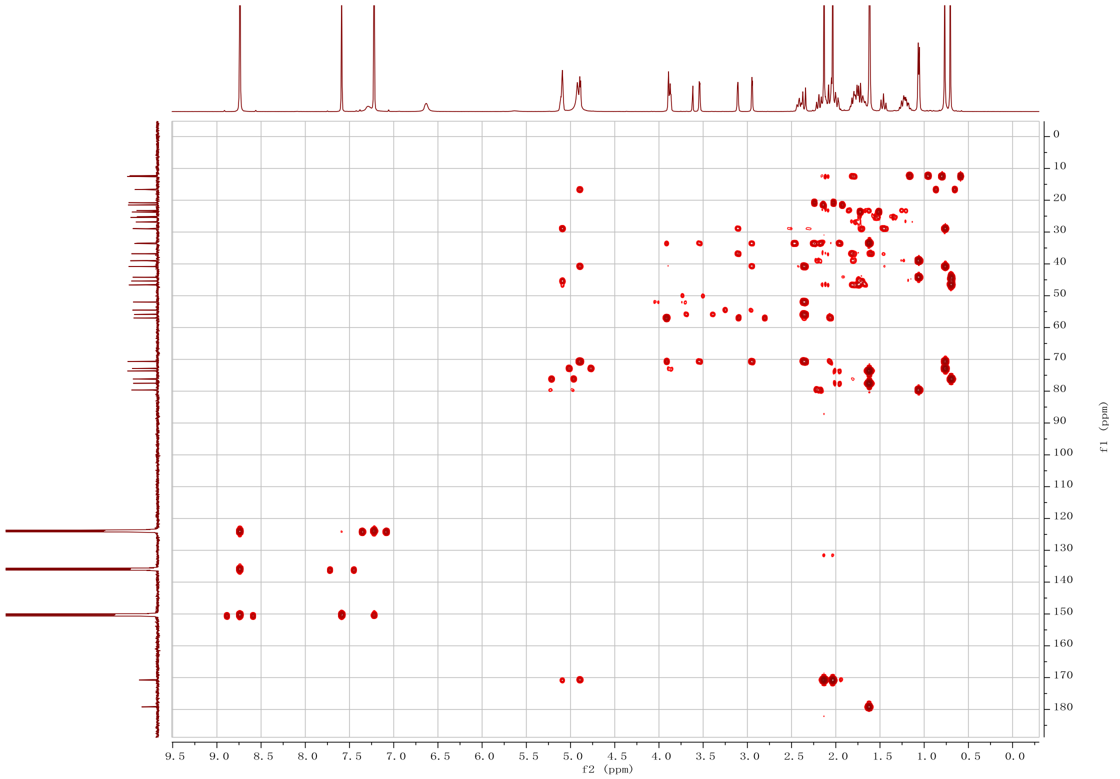

Figure S9. HMBC spectrum of chantriolide F (**1**) in C_5D_5N

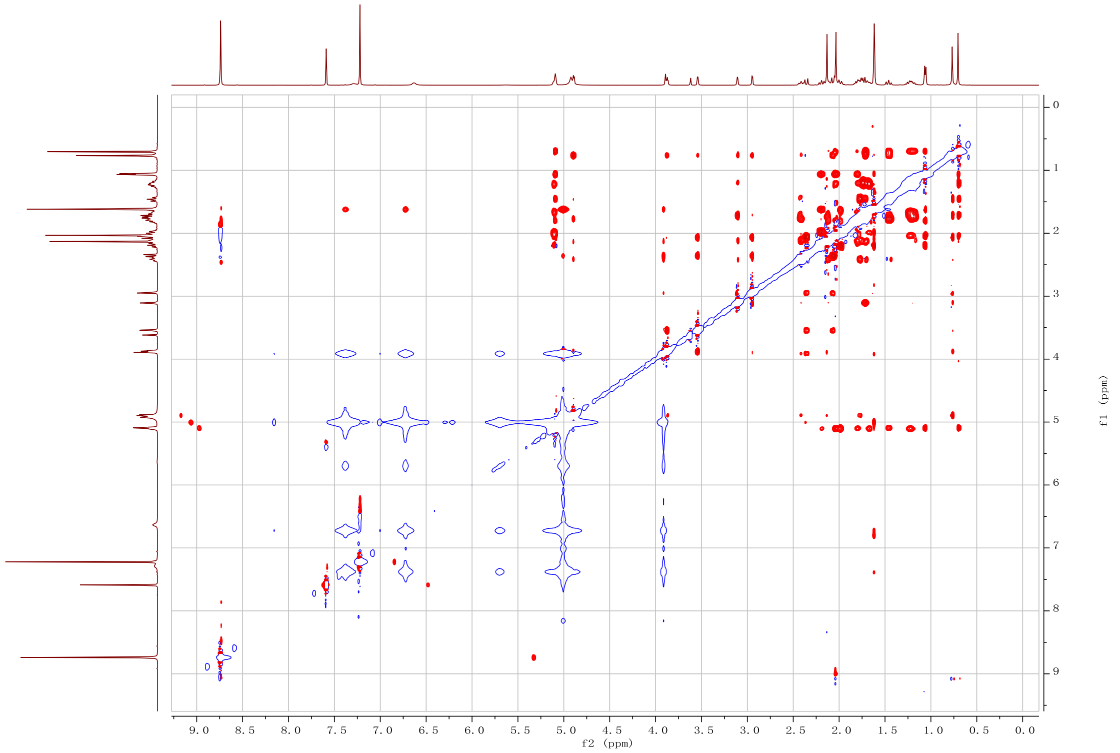

Figure S10. NOESY spectrum of chantriolide F (**1**) in C_5D_5N

Elemental Composition Report

Tolerance = 5.0 PPM / DBE: min = -1.5, max = 50.0
Element prediction: Off
Number of isotope peaks used for i-FIT = 3

Monoisotopic Mass, Even Electron Ions
104 formula(e) evaluated with 1 results within limits (up to 50 best isotopic matches for each mass)
Elements Used:
C: 0-34 H: 0-200 O: 0-30

Minimum:	80.00				-1.5				
Maximum:	100.00		5.0	5.0	50.0				
Mass	RA	Calc. Mass	mDa	PPM	DBE	i-FIT	Norm	Conf(%)	Formula
587.2865	100.00	587.2856	0.9	1.5	11.5	518.4	n/a	n/a	C32 H43 O10

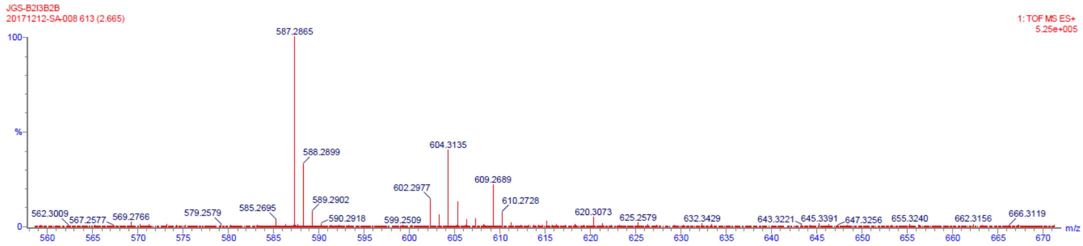

Figure S11. HRESIMS spectrum of chantriolide G (**2**)

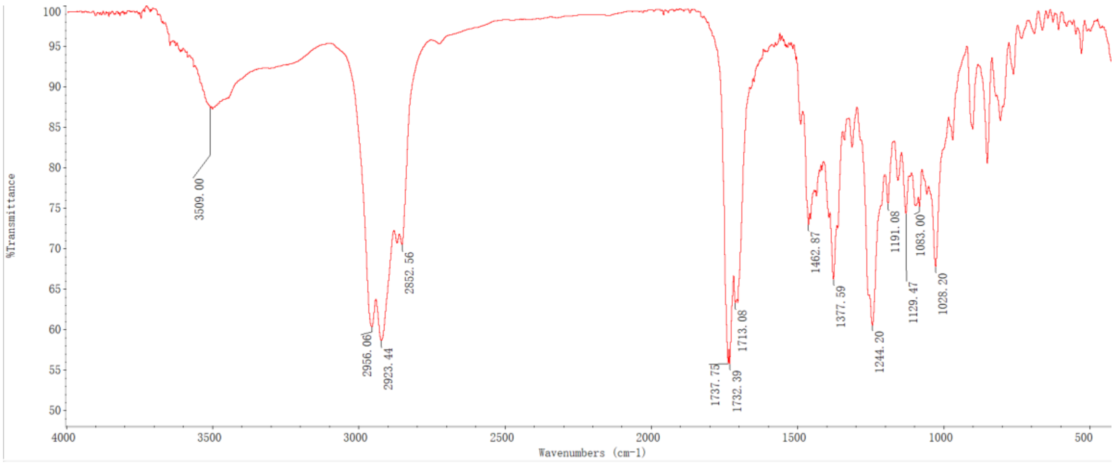

Figure S12. IR spectrum of chantriolide G (**2**)

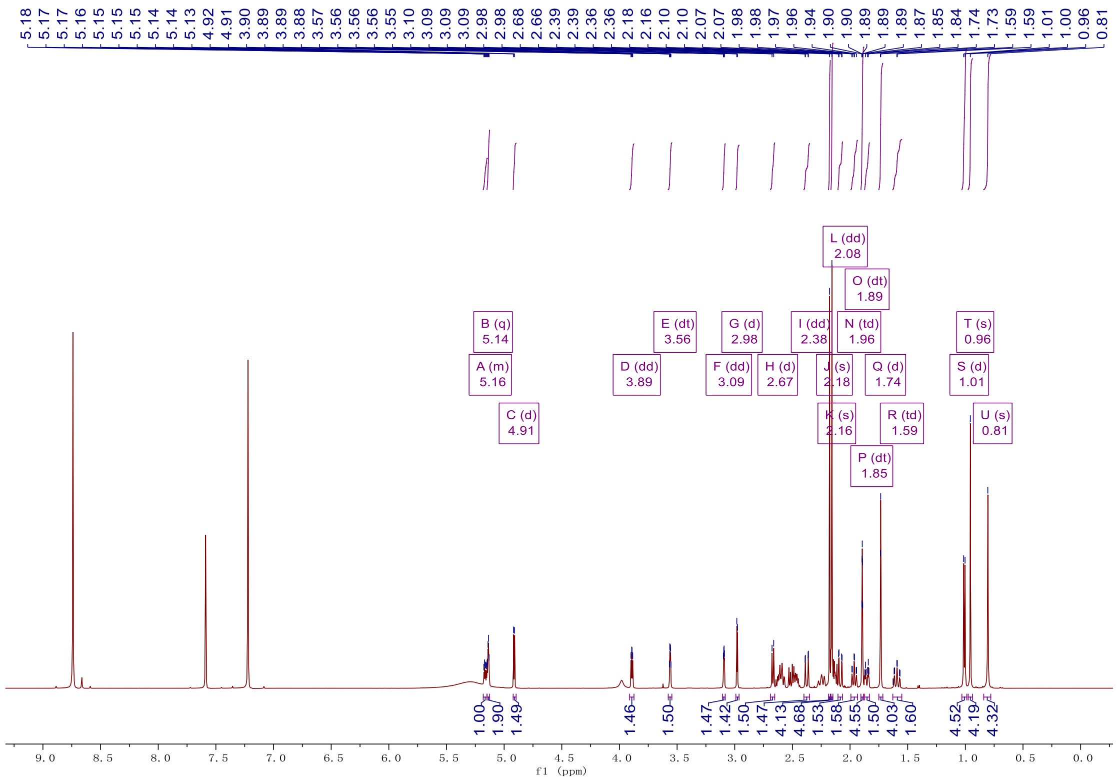

Figure S13. ^{1}H NMR (600 MHz) spectrum of chantriolide G (**2**) in C_5D_5N

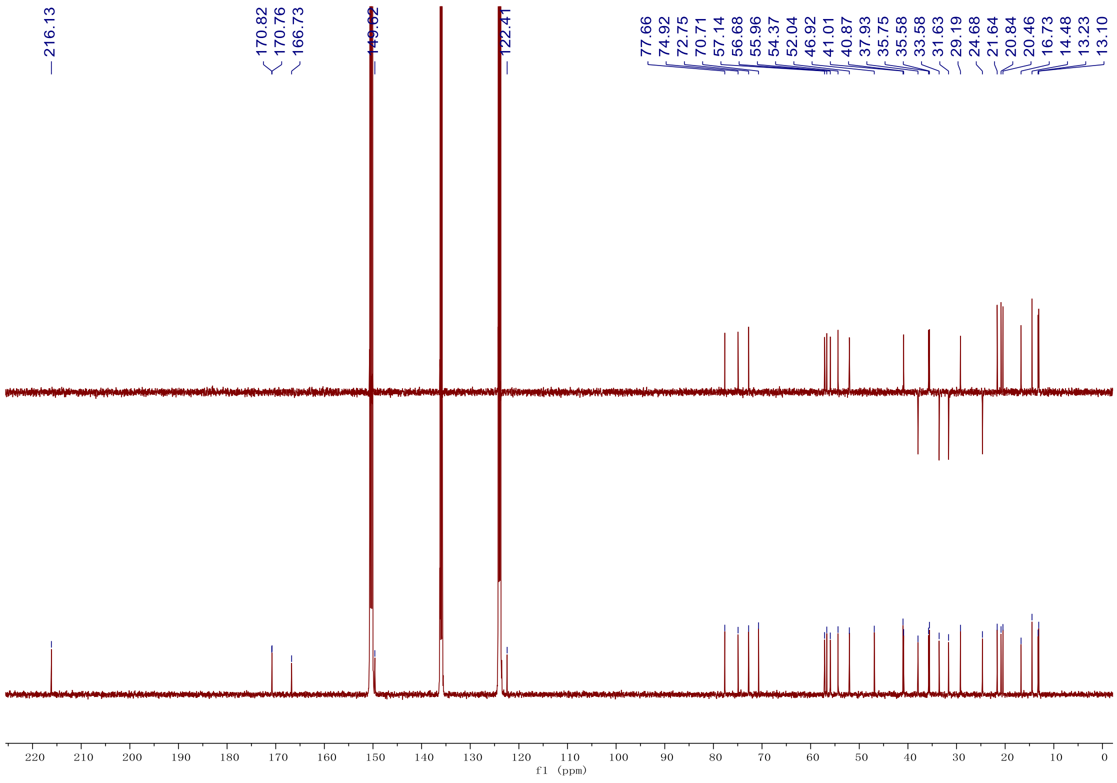

Figure S14. ^{13}C and DEPT-135 NMR (125 MHz) spectra of chantriolide G (**2**) in C_5D_5N

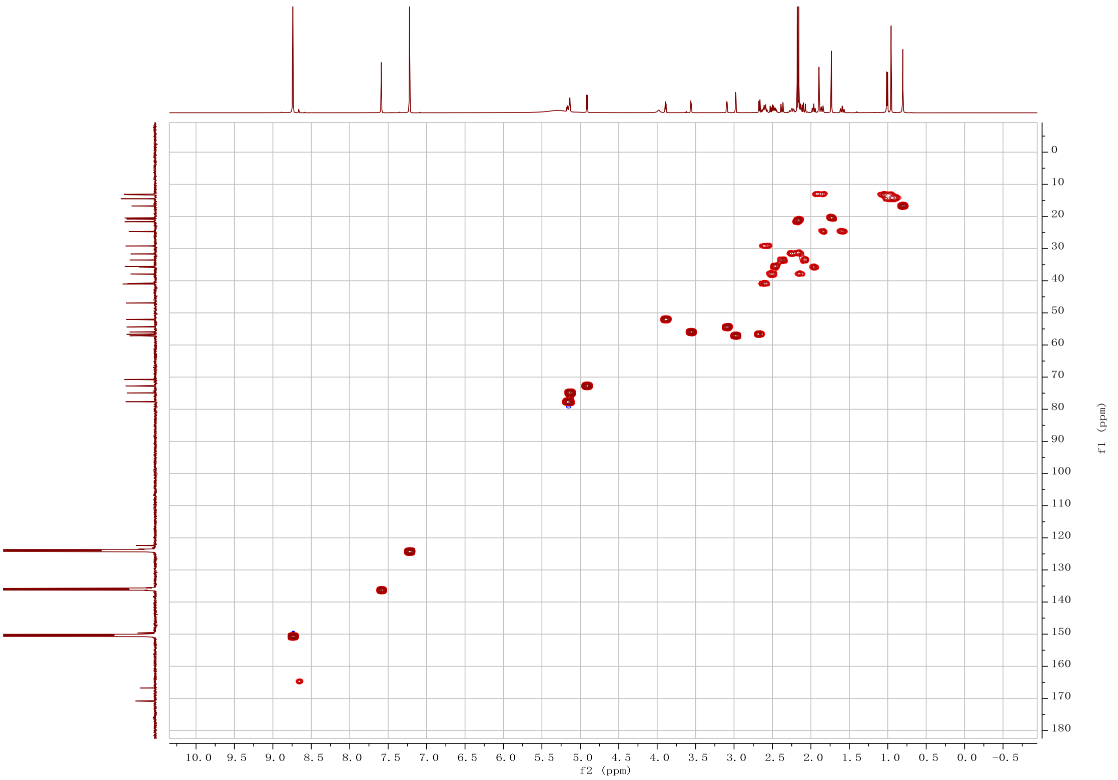

Figure S15. HSQC spectrum of chantriolide G (**2**) in C_5D_5N

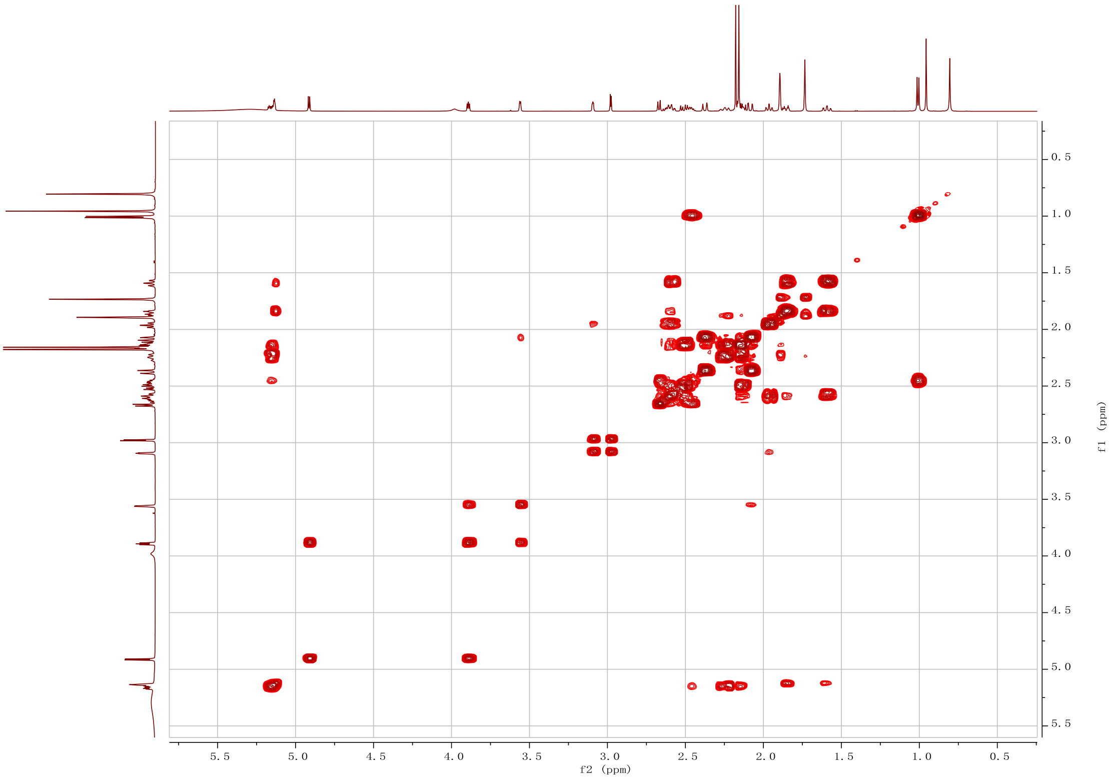

Figure S16. ^1H-^1H COSY spectrum of chantriolide G (**2**) in C_5D_5N

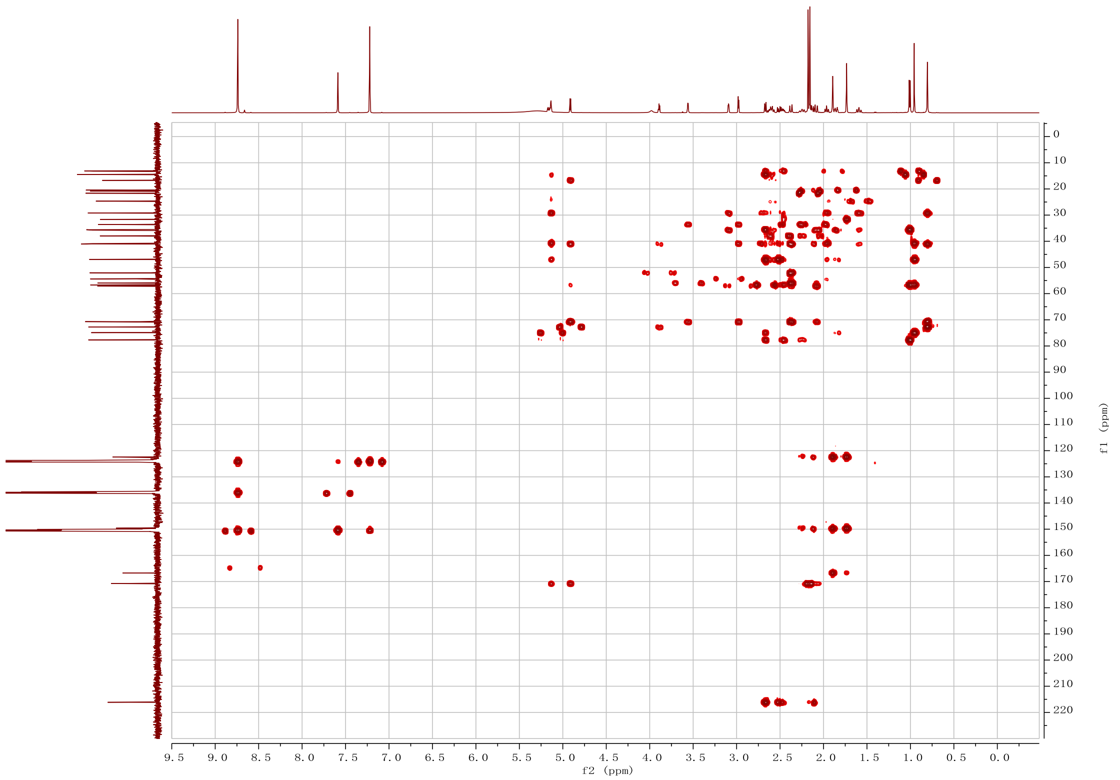

Figure S17. HMBC spectrum of chantriolide G (**2**) in C_5D_5N

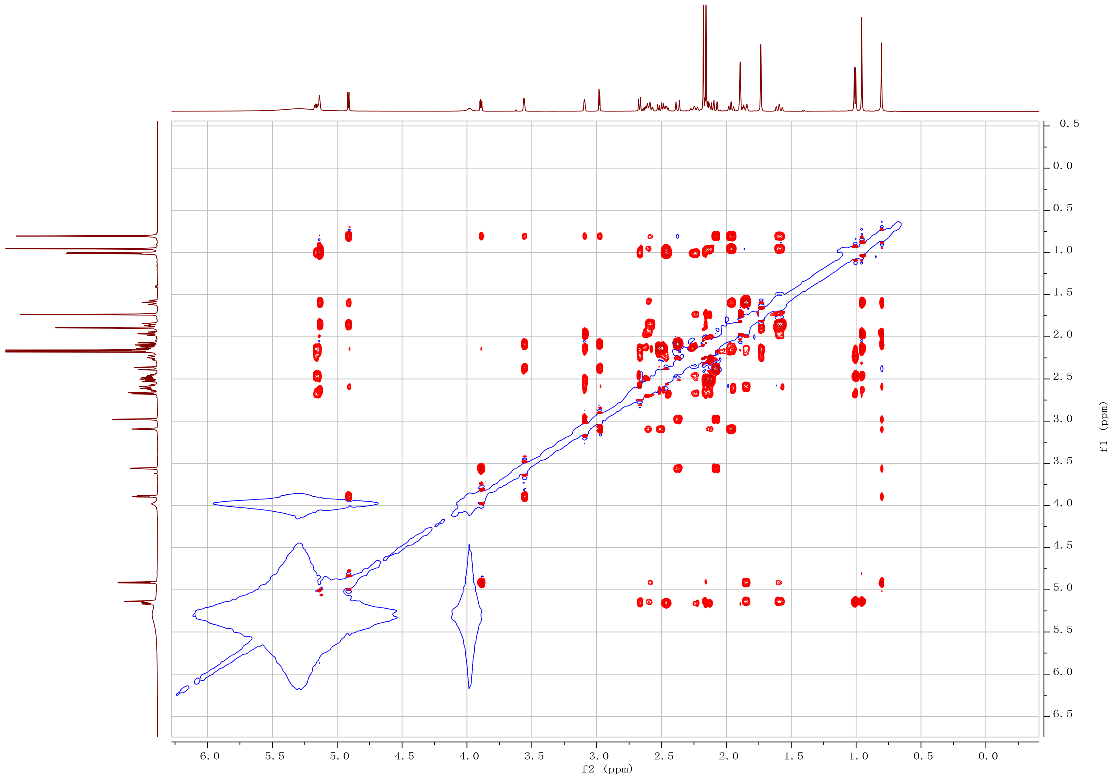

Figure S18. NOESY spectrum of chantriolide G (**2**) in C_5D_5N

Elemental Composition Report

Tolerance = 5.0 PPM / DBE: min = -1.5, max = 50.0
Element prediction: Off
Number of isotope peaks used for i-FIT = 3

Monoisotopic Mass, Even Electron Ions
105 formula(e) evaluated with 1 results within limits (up to 50 best isotopic matches for each mass)
Elements Used:
C: 0-34 H: 0-200 O: 0-30

Minimum:	80.00				-1.5				
Maximum:	100.00		5.0	5.0	50.0				
Mass	RA	Calc. Mass	mDa	PPM	DBE	i-FIT	Norm	Conf(%)	Formula
547.2914	100.00	547.2907	0.7	1.3	9.5	525.0	n/a	n/a	C30 H43 O9

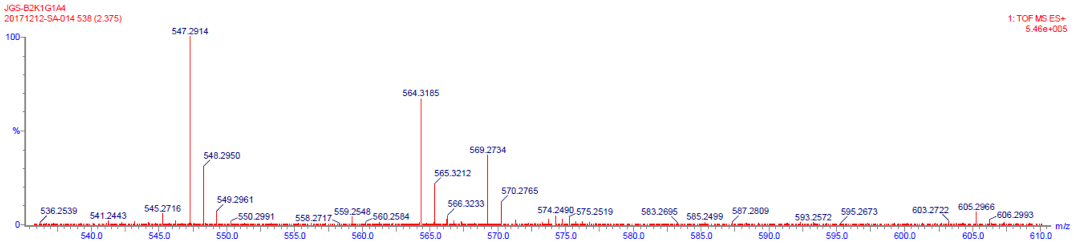

Figure S19. HRESIMS spectrum of chantriolide H (**3**)

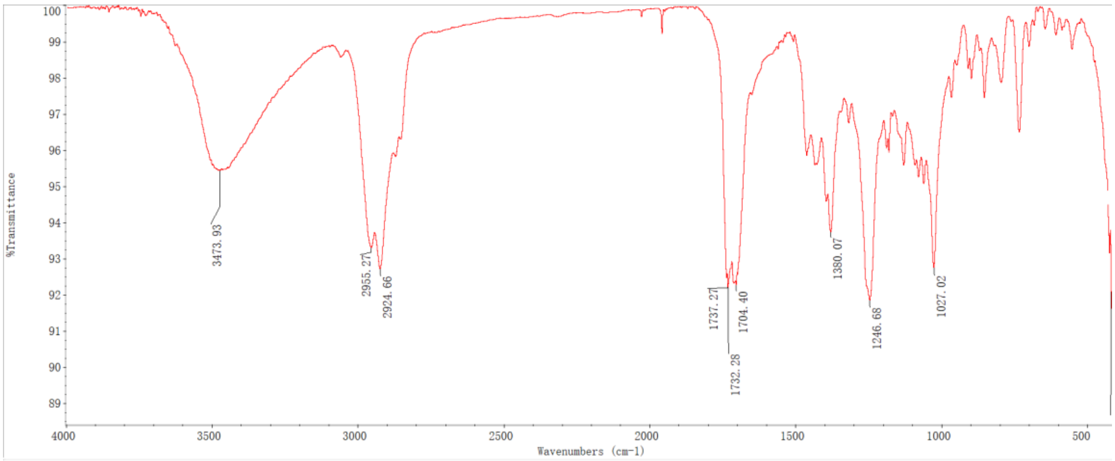

Figure S20. IR spectrum of chantriolide H (**3**)

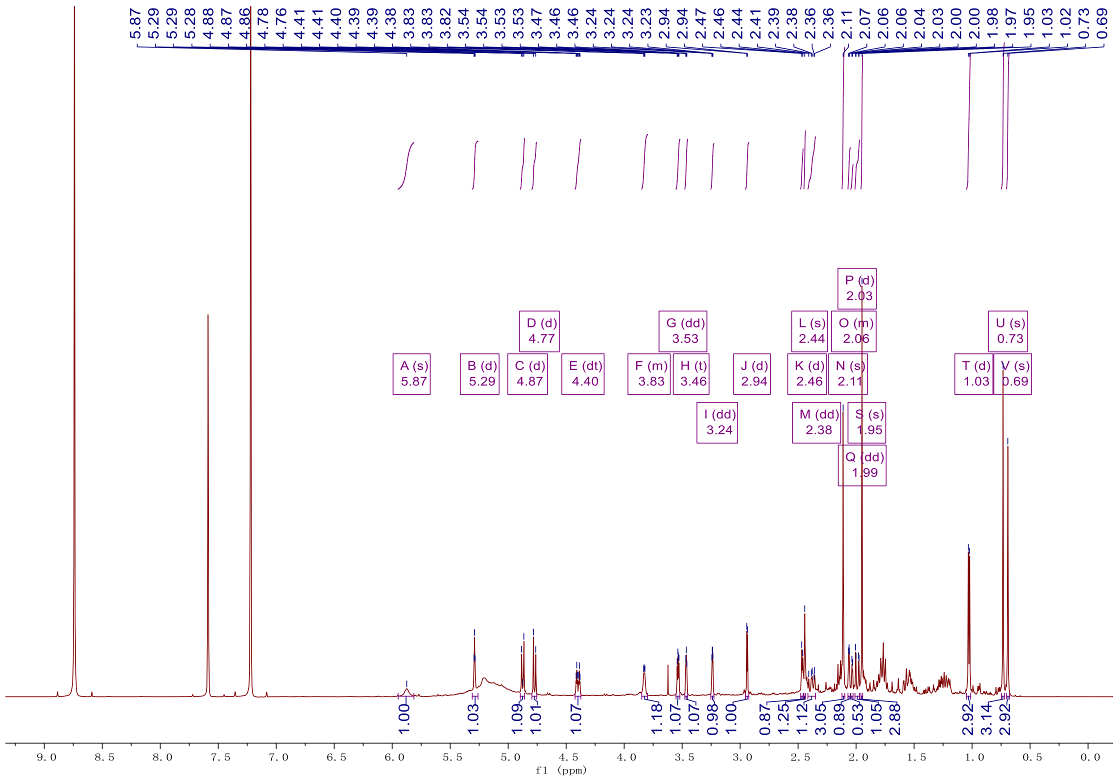

Figure S21. ^{1}H NMR (600 MHz) spectrum of chantriolide H (**3**) in C_5D_5N

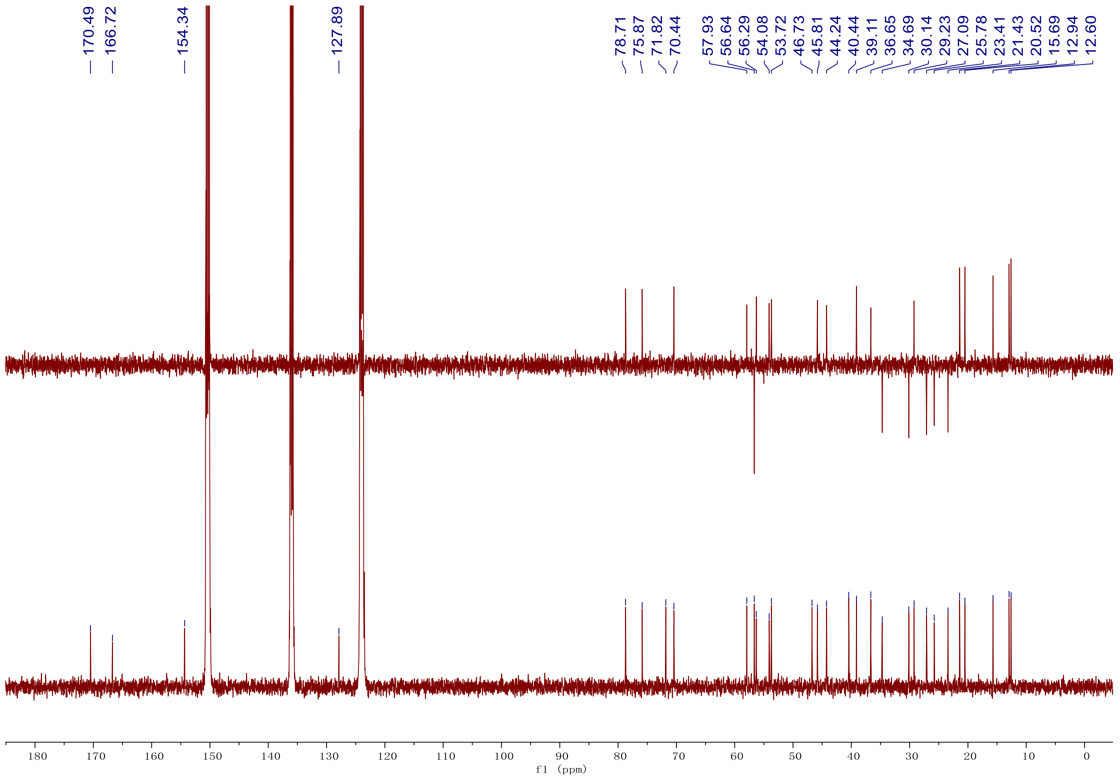

Figure S22. ^{13}C and DEPT-135 NMR (125 MHz) spectra of chantriolide H (**3**) in C_5D_5N

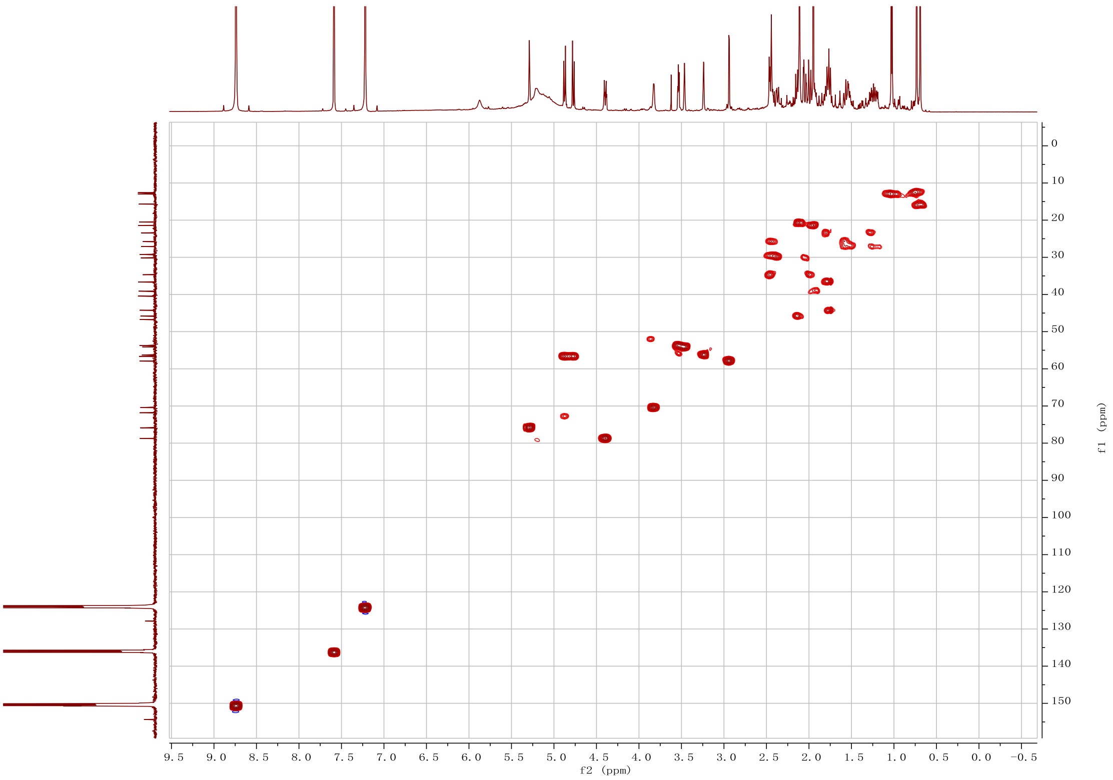

Figure S23. HSQC spectrum of chantriolide H (**3**) in C_5D_5N

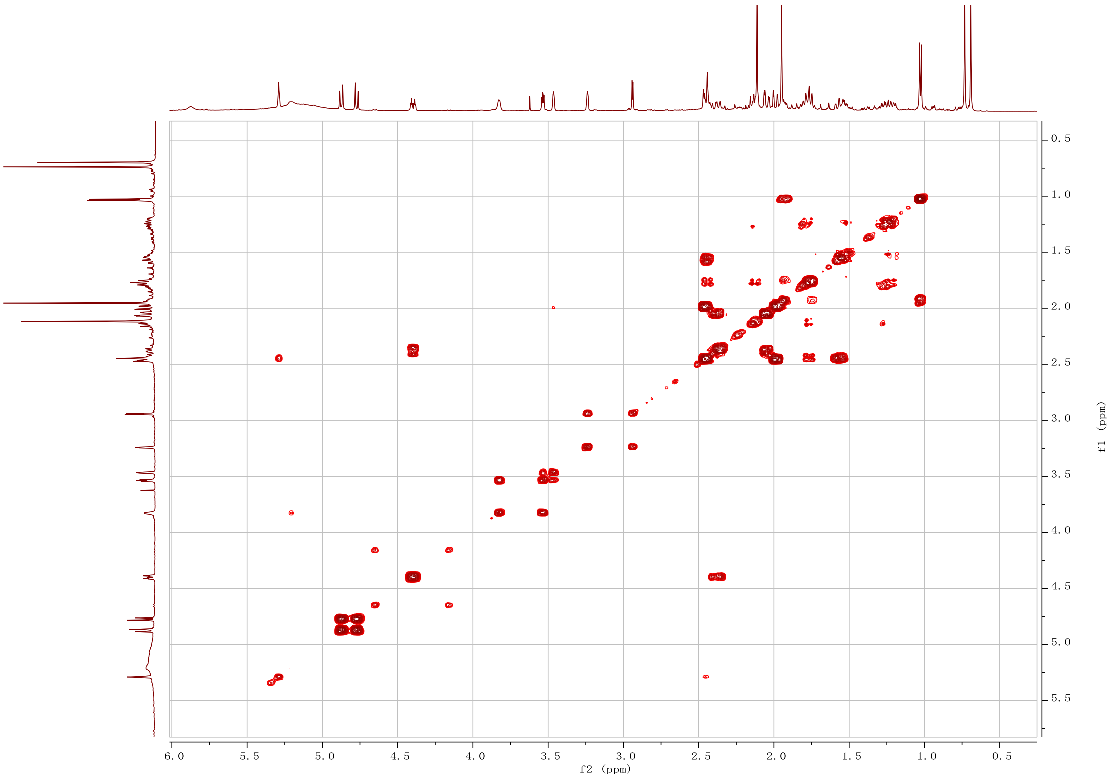

Figure S24. ^{1}H-^{1}H COSY spectrum of chantriolide H (**3**) in C_5D_5N

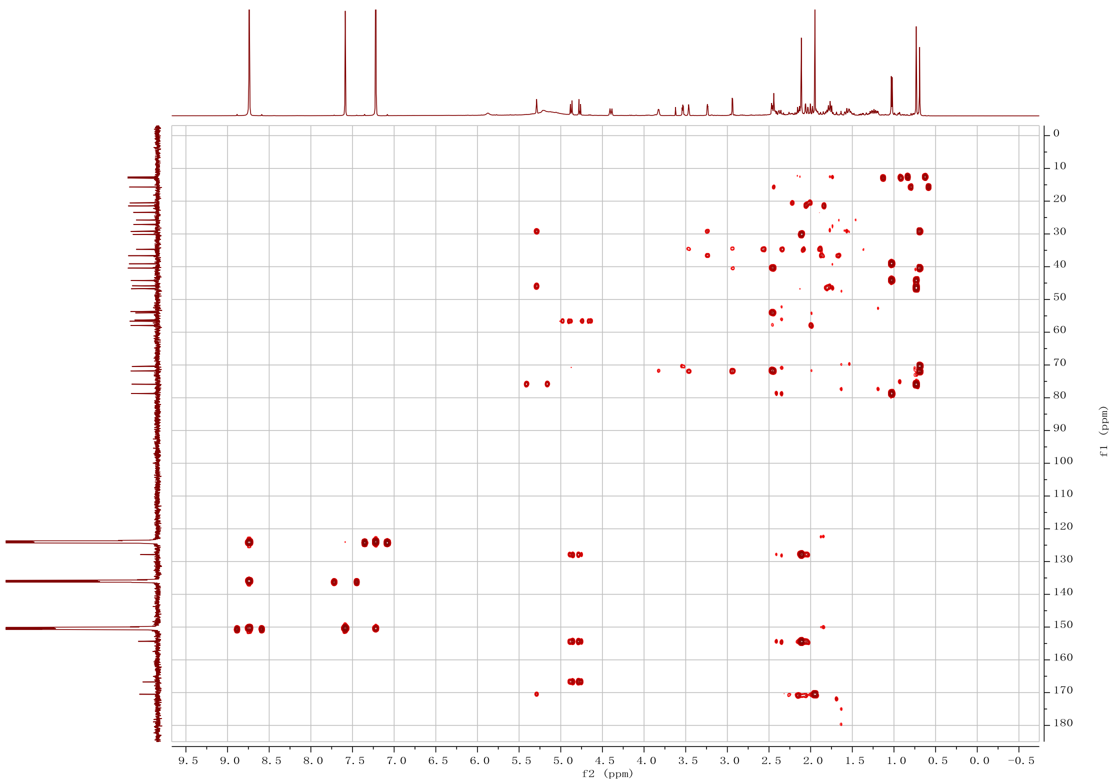

Figure S25. HMBC spectrum of chantriolide H (**3**) in C_5D_5N

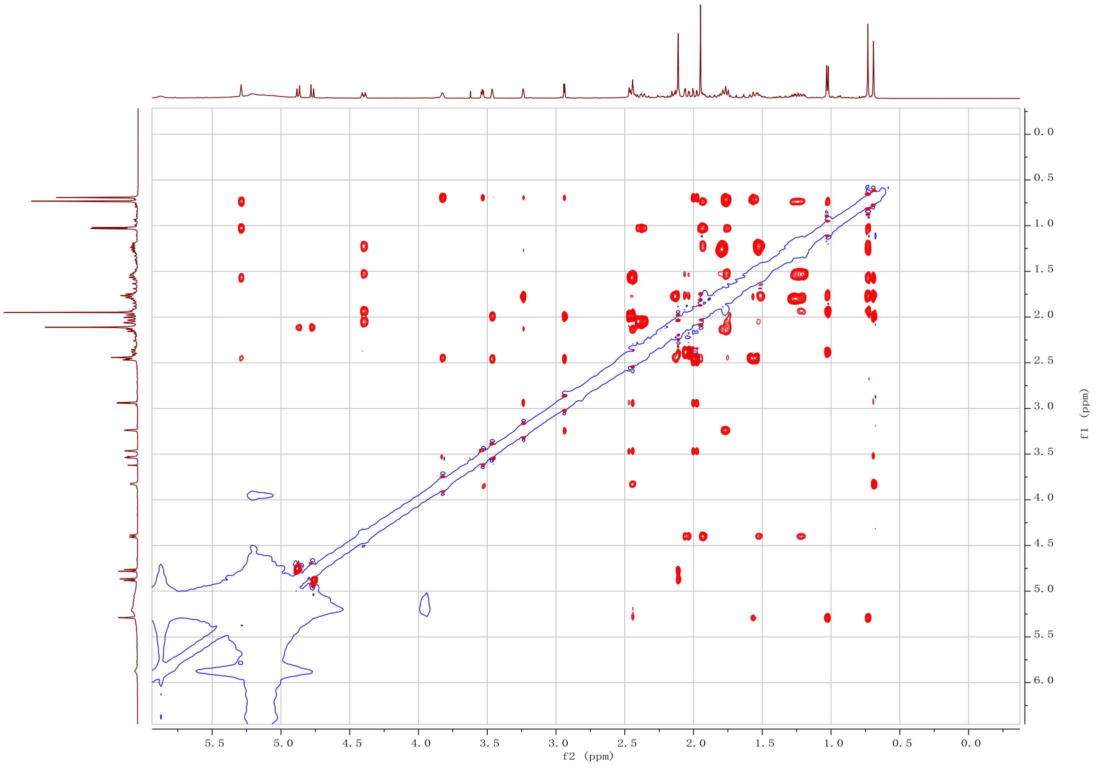

Figure S26. NOESY spectrum of chantriolide H (**3**) in C_5D_5N

Elemental Composition Report

Tolerance = 5.0 PPM / DBE: min = -1.5, max = 50.0
Element prediction: Off
Number of isotope peaks used for i-FIT = 3

Monoisotopic Mass, Even Electron Ions
196 formula(e) evaluated with 1 results within limits (up to 50 best isotopic matches for each mass)
Elements Used:
C: 0-34 H: 0-200 N: 0-1 O: 0-30

Minimum:	80.00				-1.5				
Maximum:	100.00		5.0	5.0	50.0				
Mass	RA	Calc. Mass	mDa	PPM	DBE	i-FIT	Norm	Conf(%)	Formula
622.3237	100.00	622.3227	1.0	1.6	9.5	475.6	n/a	n/a	C32 H48 N O11

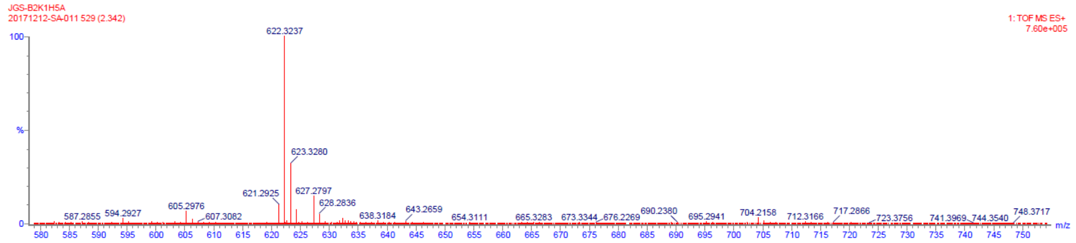

Figure S27. HRESIMS spectrum of chantriolide I (**4**)

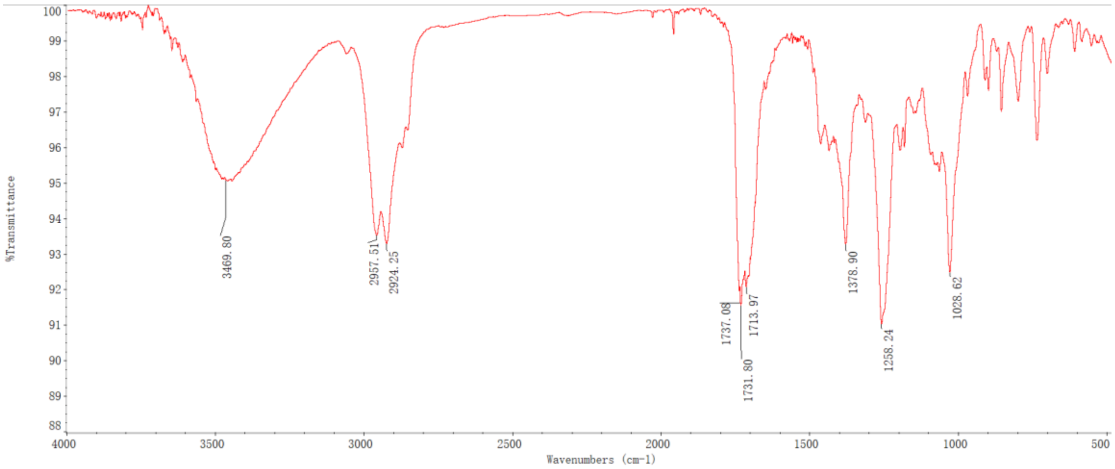

Figure S28. IR spectrum of chantriolide I (**4**)

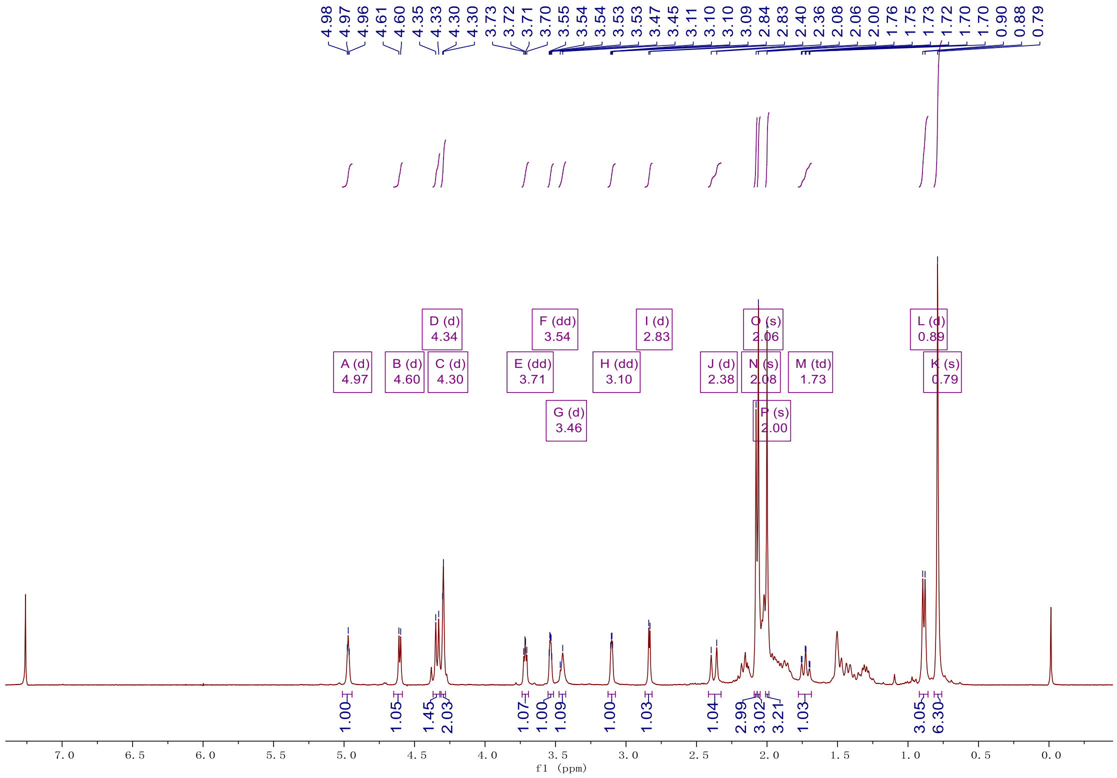

Figure S29. ^{1}H NMR (400 MHz) spectrum of chantriolide I (**4**) in $CDCl_3$

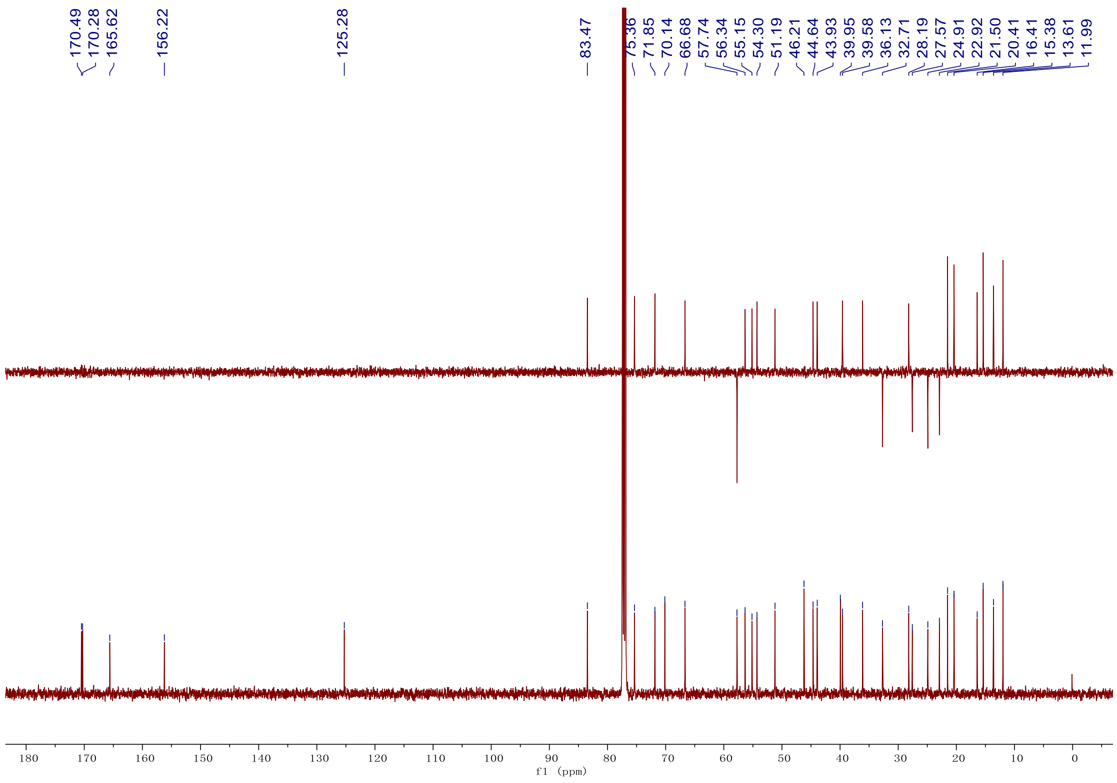

Figure S30. ^{13}C and DEPT-135 NMR (125 MHz) spectra of chantriolide I (**4**) in $CDCl_3$

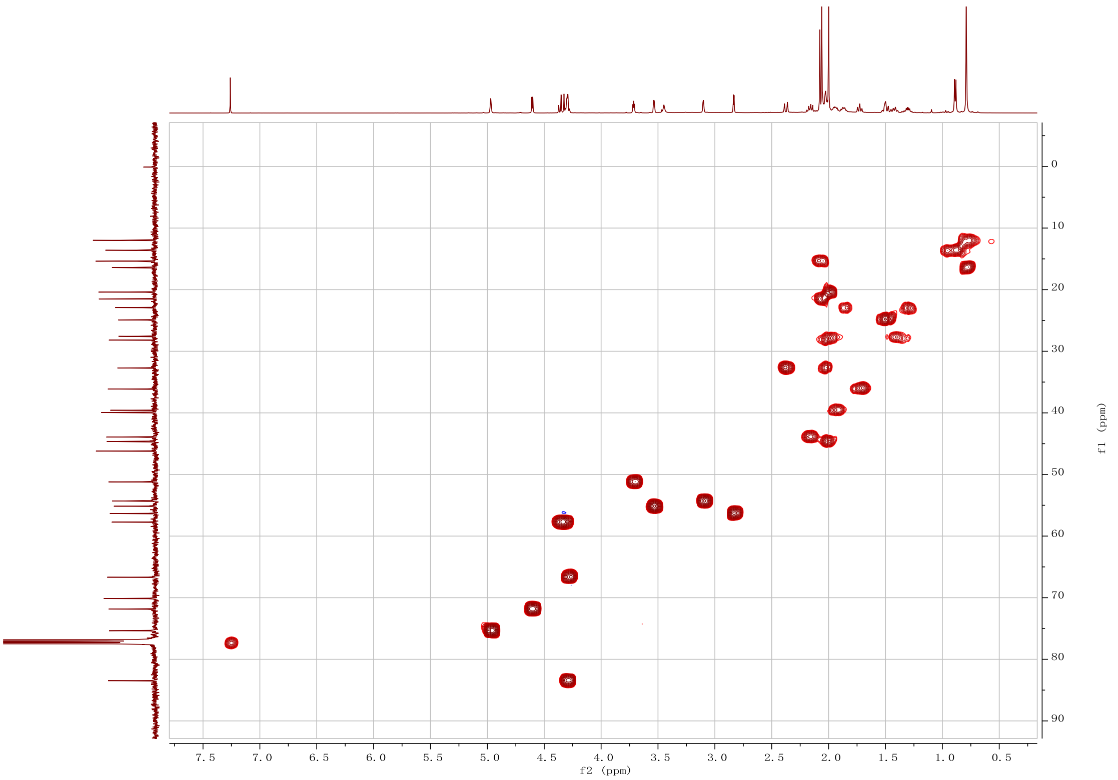

Figure S31. HSQC spectrum of chantriolide I (**4**) in $CDCl_3$

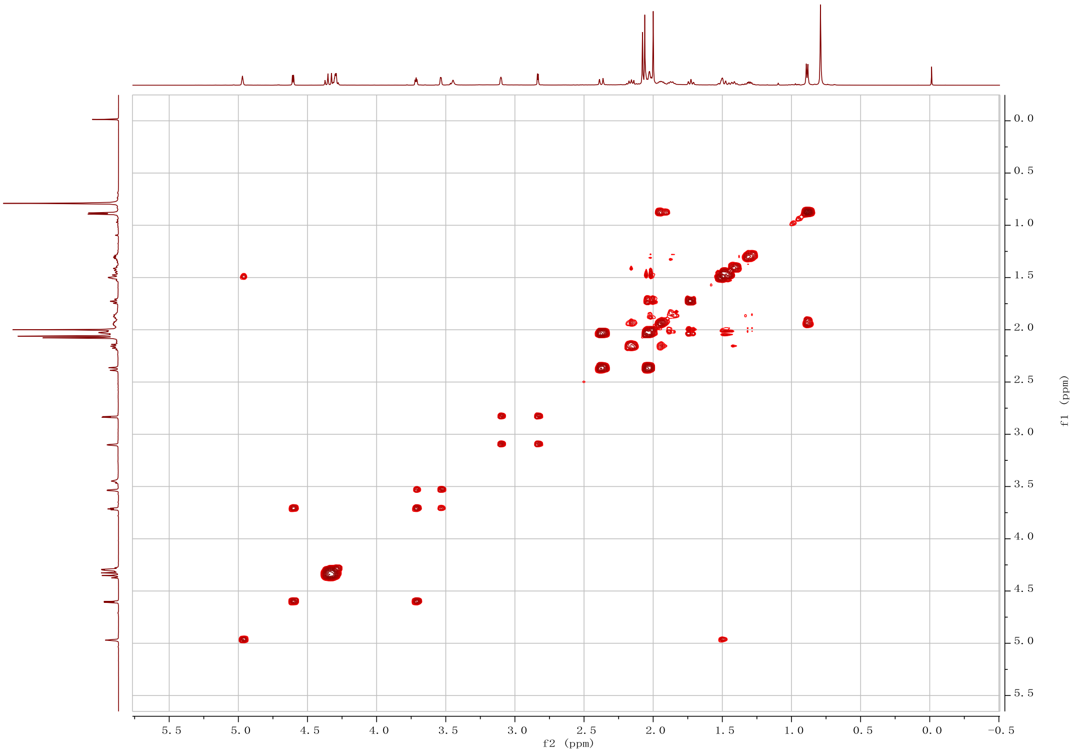

Figure S32. ^1H-^1H COSY spectrum of chantriolide I (**4**) in $CDCl_3$

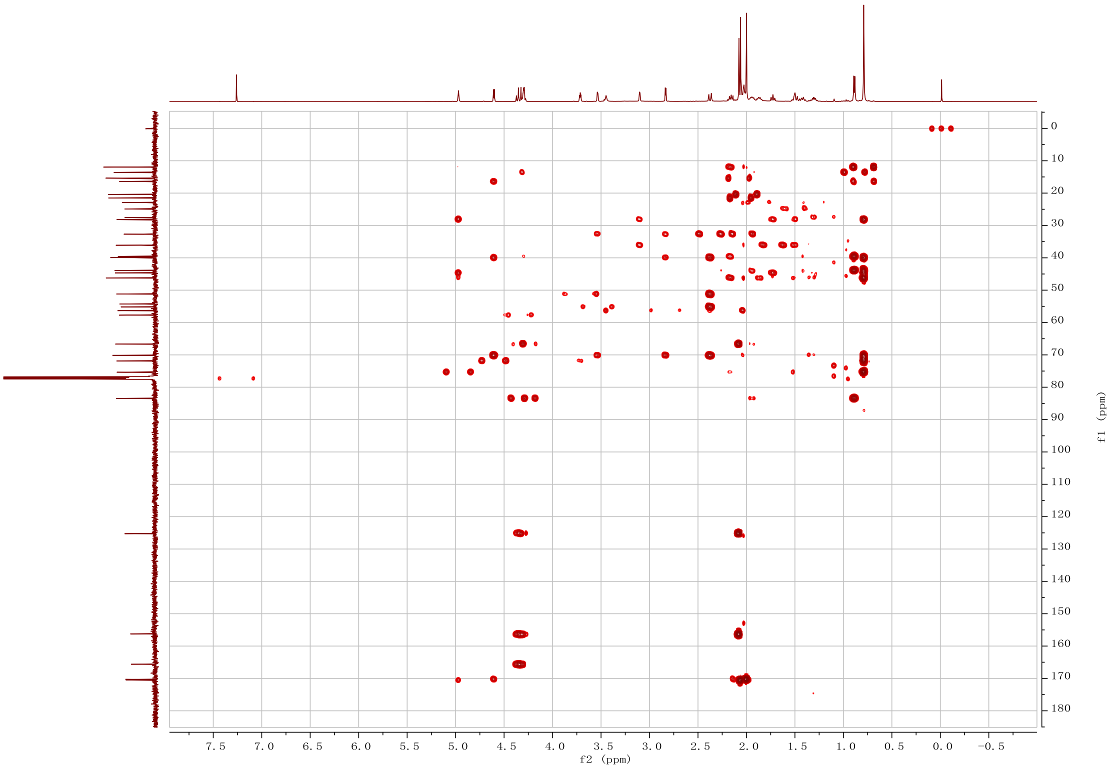

Figure S33. HMBC spectrum of chantriolide I (**4**) in $CDCl_3$

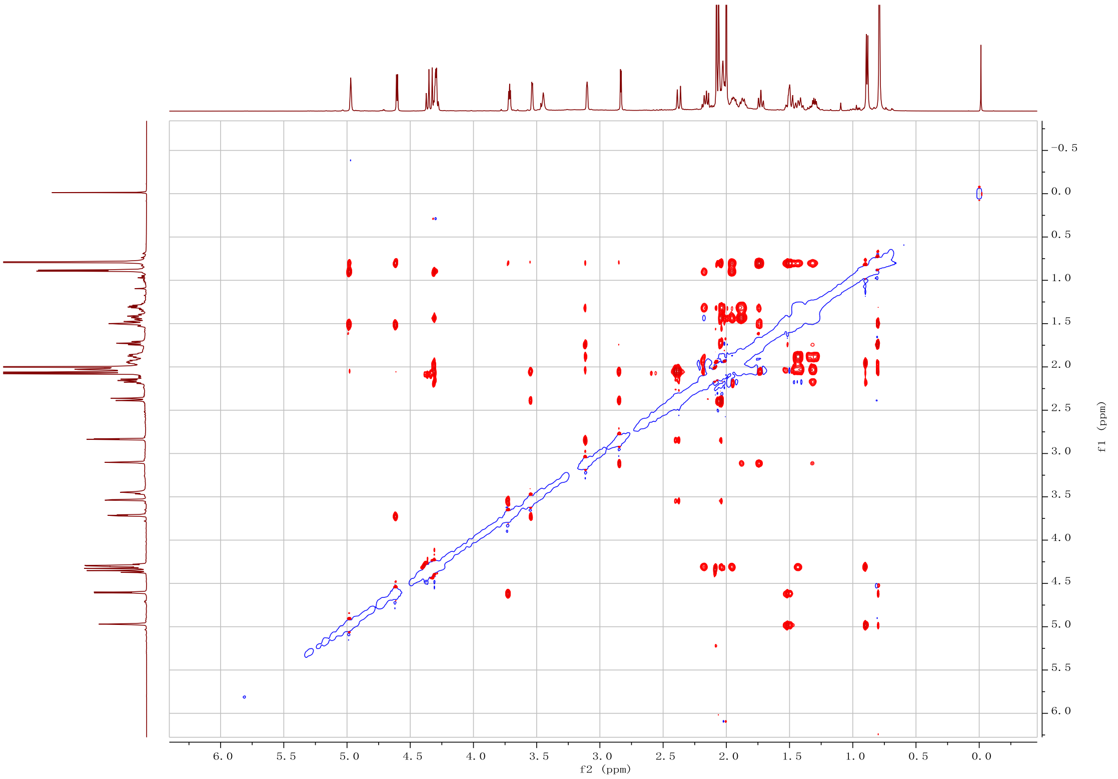

Figure S34. ROESY spectrum of chantriolide I (**4**) in $CDCl_3$

Elemental Composition Report

Single Mass Analysis

Tolerance = 10.0 PPM / DBE: min = -1.5, max = 50.0

Element prediction: Off

Number of isotope peaks used for i-FIT = 3

Monoisotopic Mass, Even Electron Ions

161 formula(e) evaluated with 3 results within limits (up to 10 best isotopic matches for each mass)

Elements Used:

C: 30-50 H: 0-60 O: 0-20 Cl: 0-1

Minimum:				-1.5				
Maximum:		5.0	10.0	50.0				
Mass	Calc. Mass	mDa	PPM	DBE	i-FIT	Norm	Conf(%)	Formula
669.2689	669.2678	1.1	1.6	10.5	625.5	0.000	100.00	C33 H46 O12 Cl

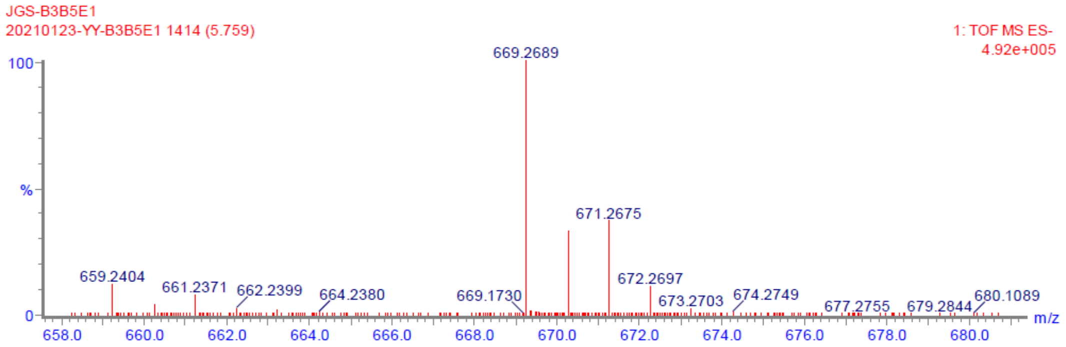

Figure S35. HRESIMS spectrum of chantriolide J (**5**)

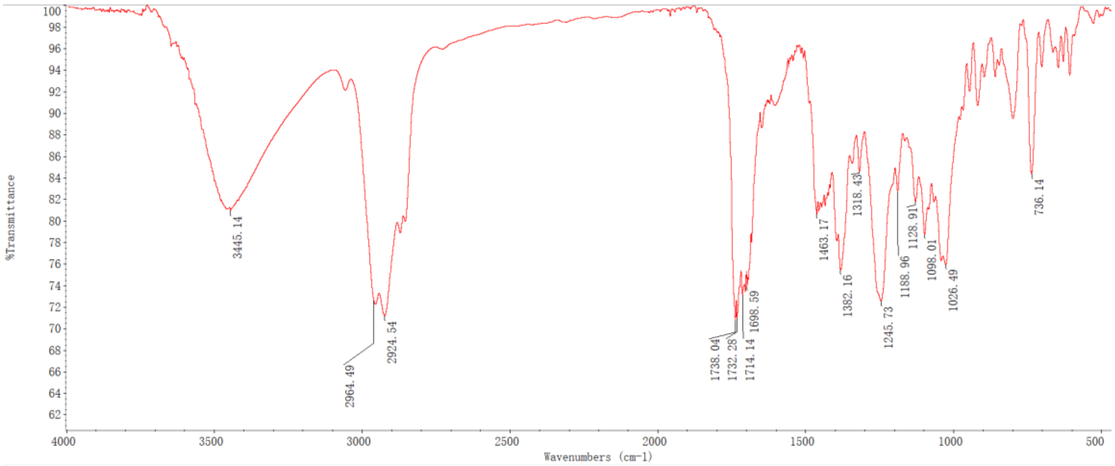

Figure S36. IR spectrum of chantriolide J (**5**)

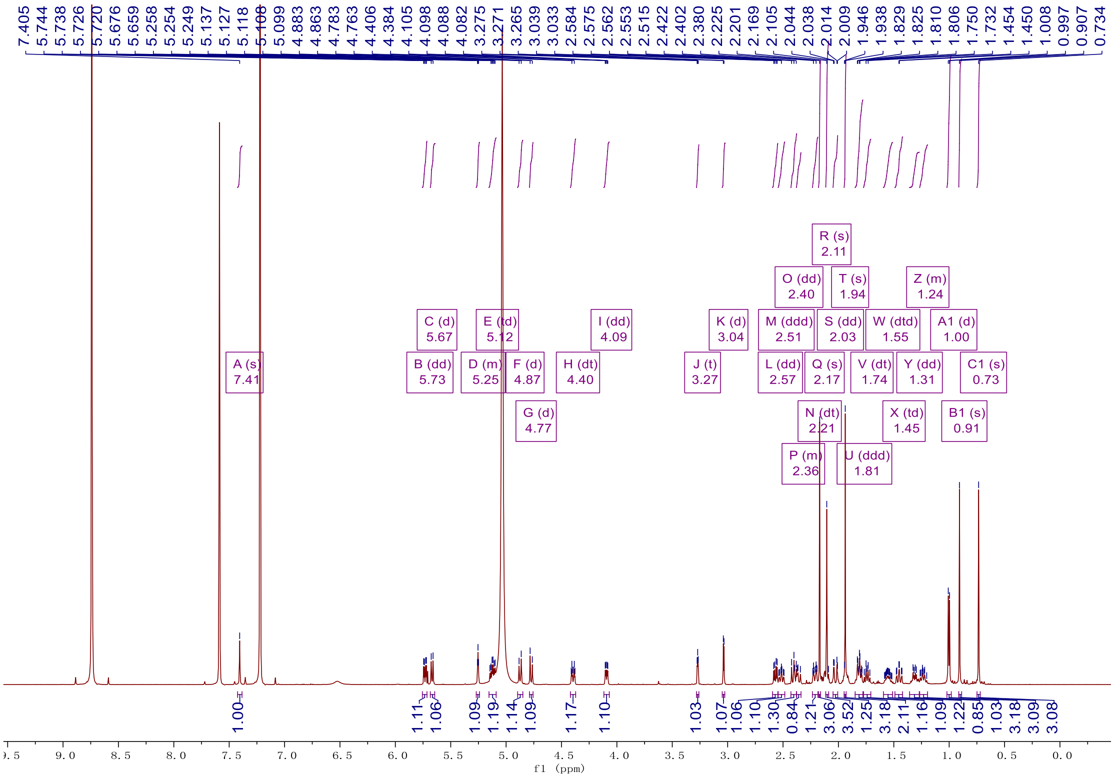

Figure S37. ^{1}H NMR (600 MHz) spectrum of chantriolide J (**5**) in C_5D_5N

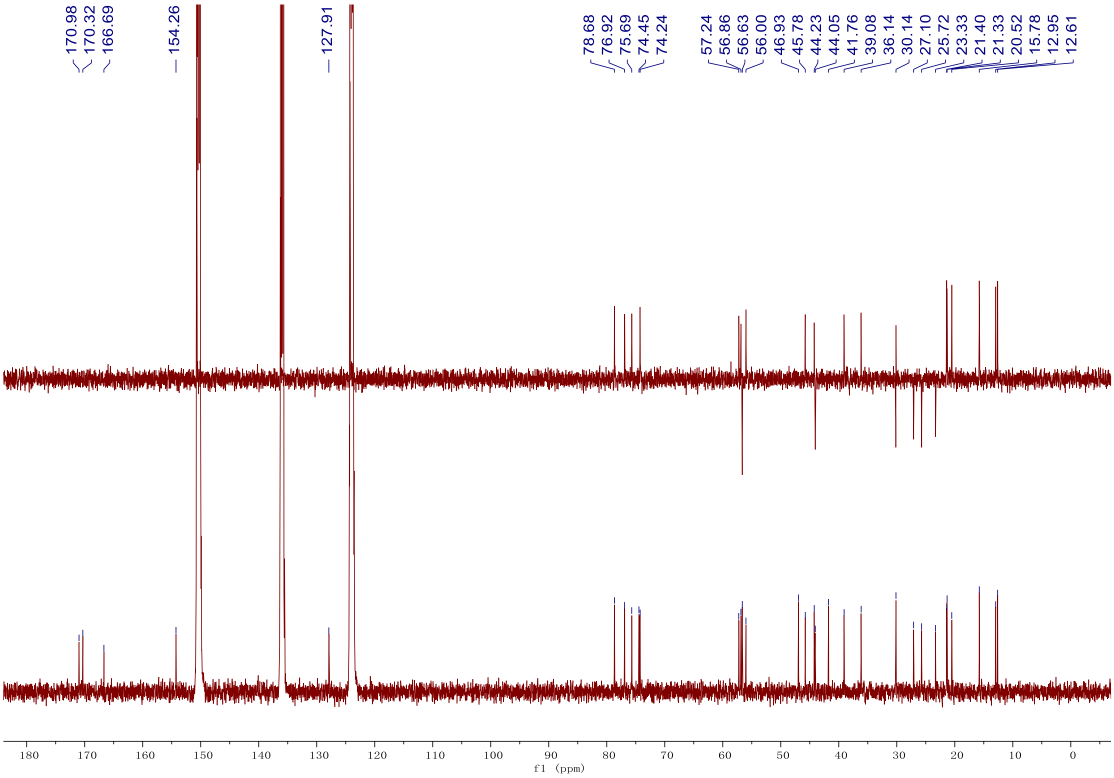

Figure S38. ^{13}C and DEPT-135 NMR (125 MHz) spectra of chantriolide J (**5**) in C_5D_5N

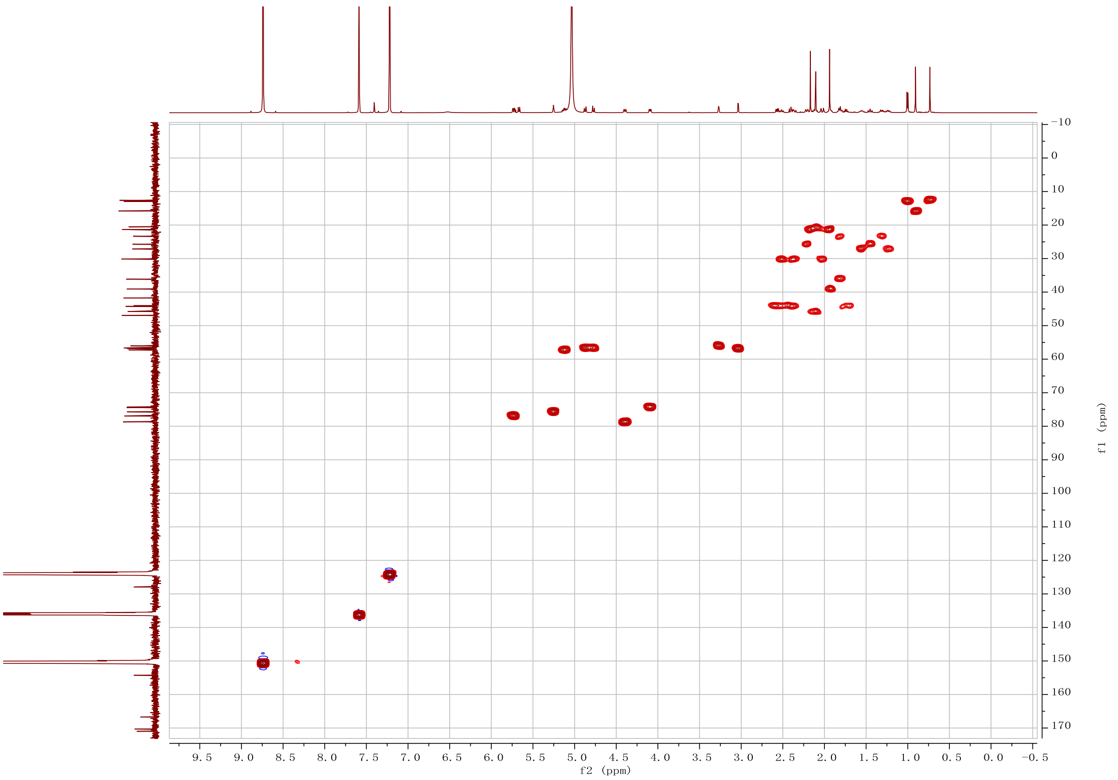

Figure S39. HSQC spectrum of chantriolide J (**5**) in C_5D_5N

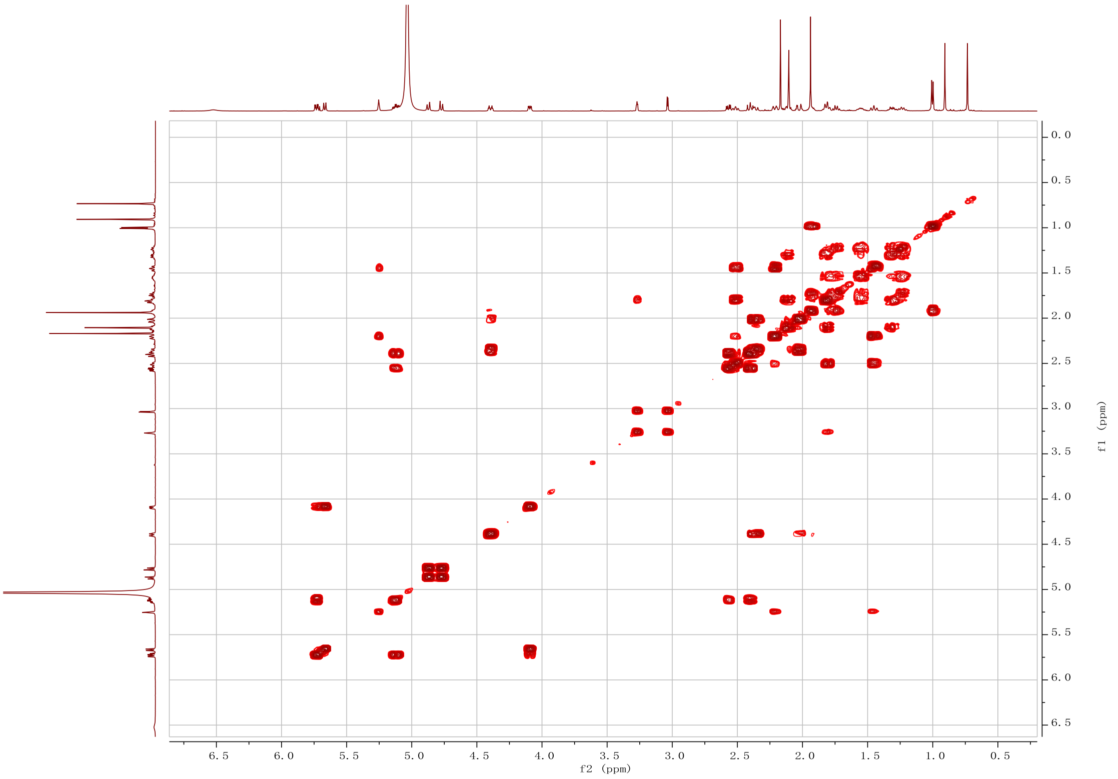

Figure S40. ^1H-^1H COSY spectrum of chantriolide J (**5**) in C_5D_5N

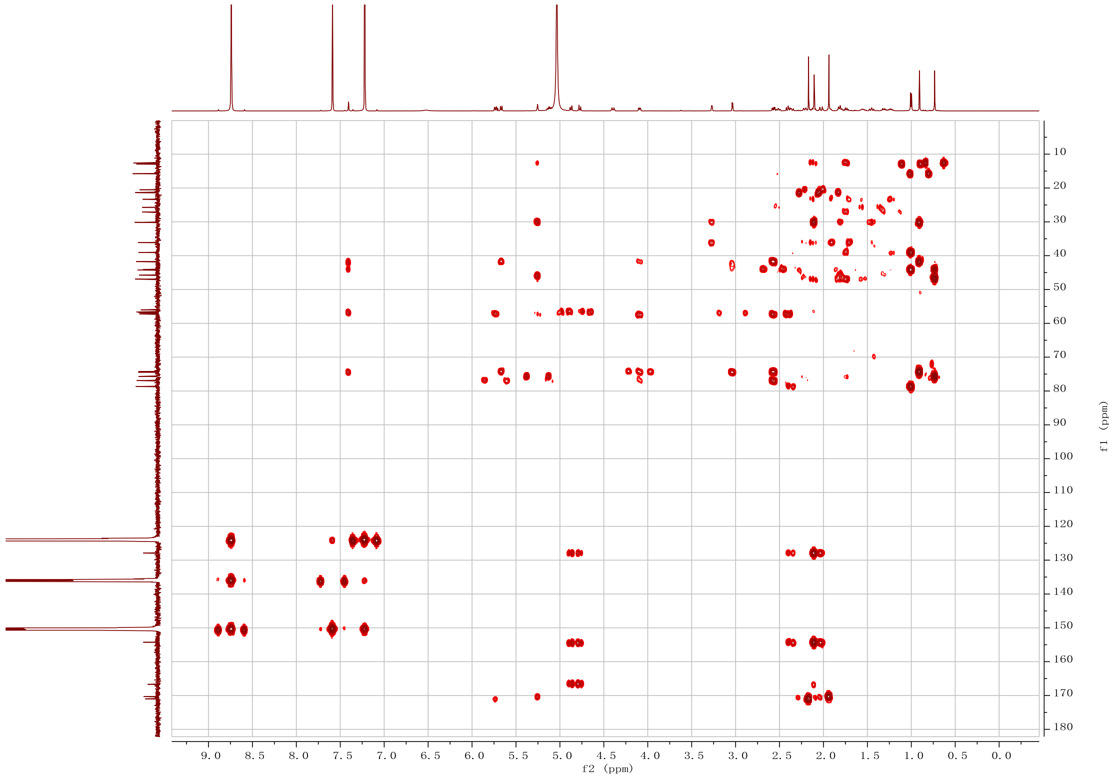

Figure S41. HMBC spectrum of chantriolide J (**5**) in C_5D_5N

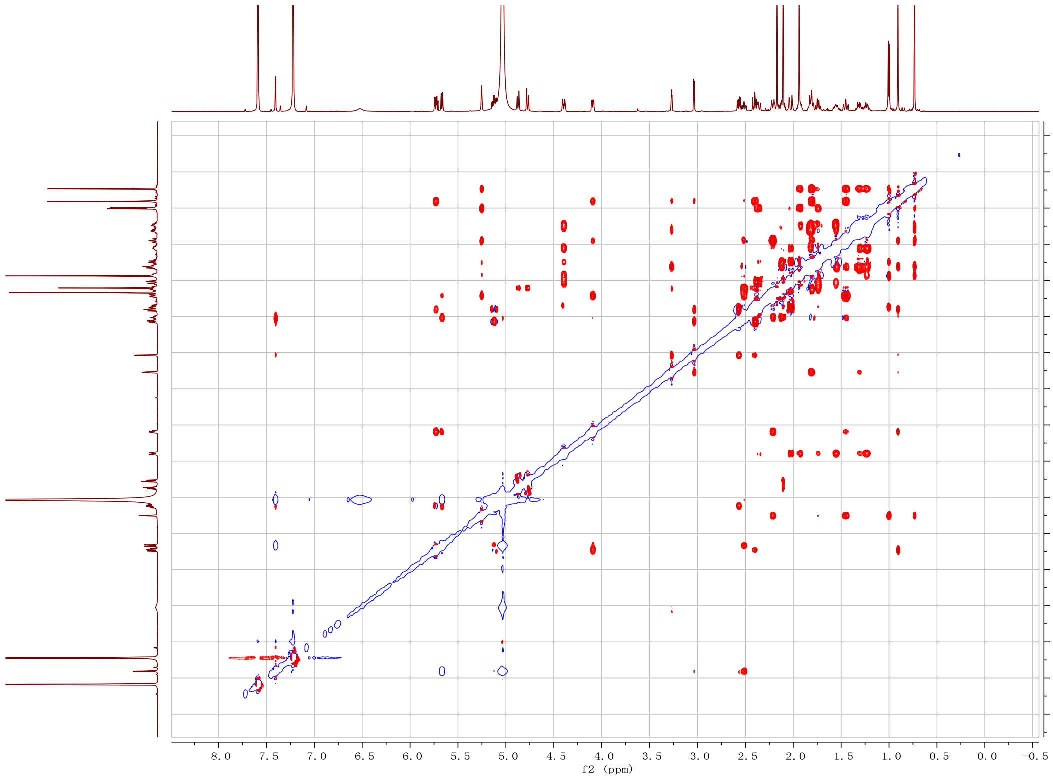

Figure S42. ROESY spectrum of chantriolide J (**5**) in C_5D_5N

Elemental Composition Report

Single Mass Analysis

Tolerance = 5.0 PPM / DBE: min = -1.5, max = 50.0

Element prediction: Off

Number of isotope peaks used for i-FIT = 3

Monoisotopic Mass, Even Electron Ions

1102 formula(e) evaluated with 7 results within limits (up to 10 closest results for each mass)

Elements Used:

C: 20-50 H: 0-70 N: 0-1 O: 0-15 Na: 0-1 Cl: 0-2

Minimum:				-1.5				
Maximum:		5.0	5.0	50.0				
Mass	Calc. Mass	mDa	PPM	DBE	i-FIT	Norm	Conf(%)	Formula
641.2735	641.2729	0.6	0.9	9.5	506.2	0.471	62.42	C32 H46 O11 Cl

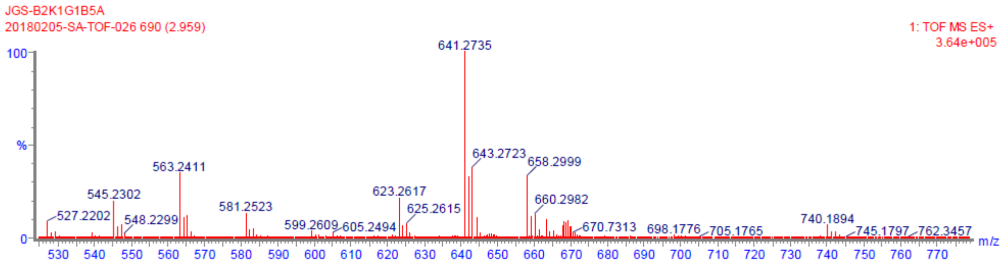

Figure S43. HRESIMS spectrum of chantriolide K (**6**)

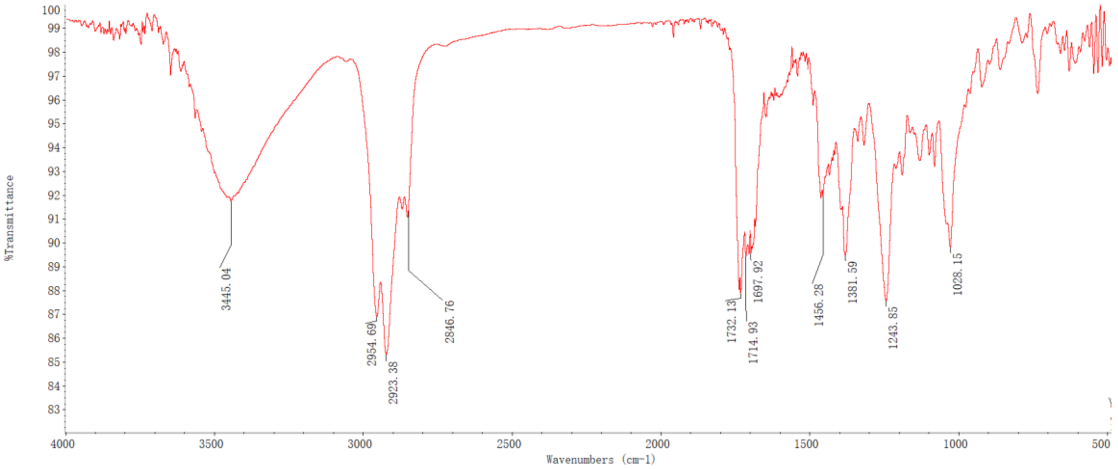

Figure S44. IR spectrum of chantriolide K (**6**)

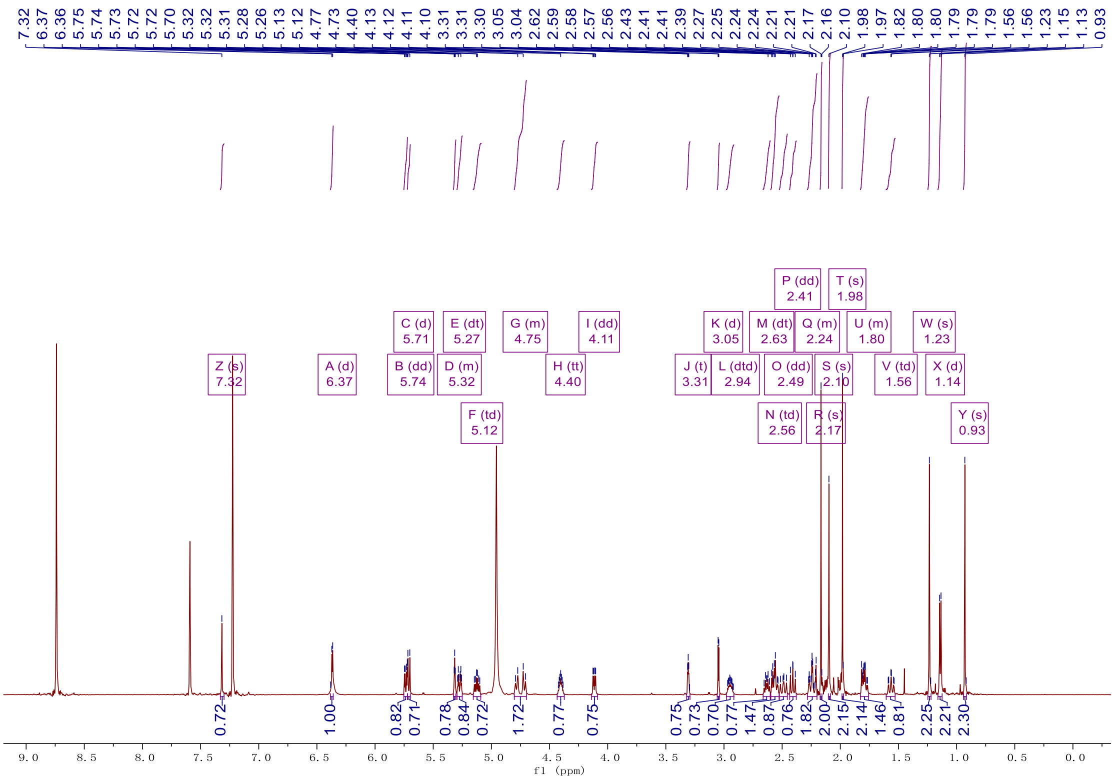

Figure S45. ^{1}H NMR (600 MHz) spectrum of chantriolide K (**6**) in C_5D_5N

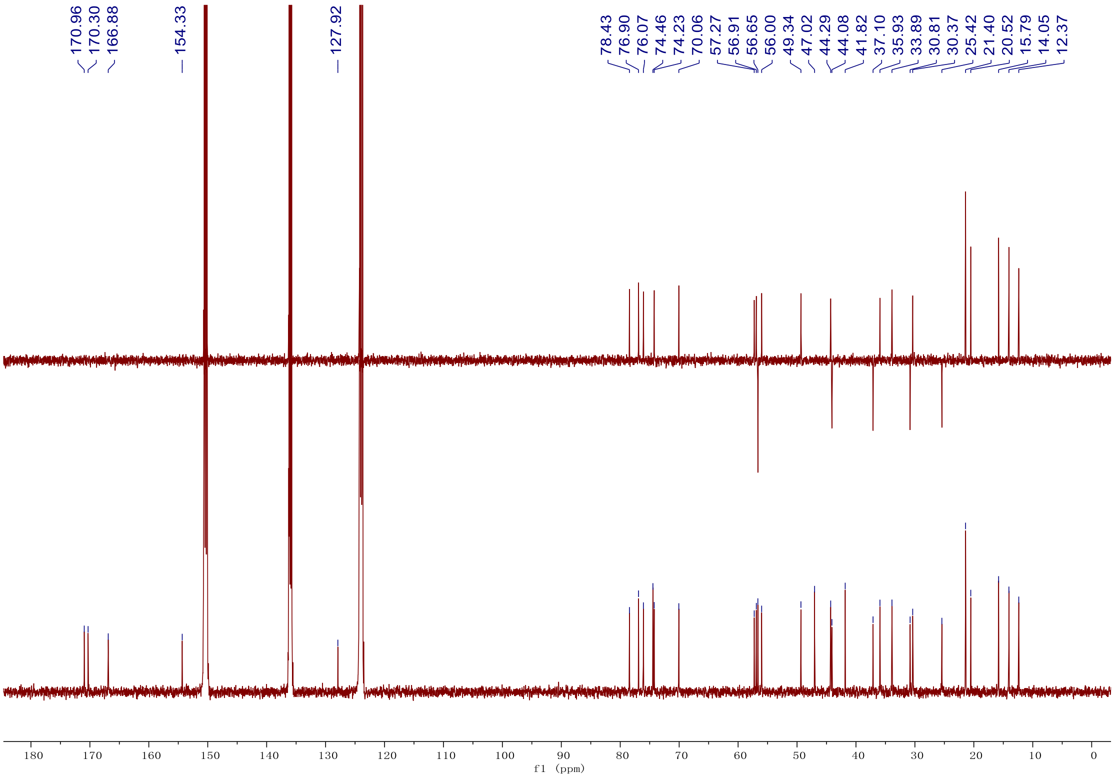

Figure S46. ^{13}C and DEPT-135 NMR (125 MHz) spectra of chantriolide K (**6**) in C_5D_5N

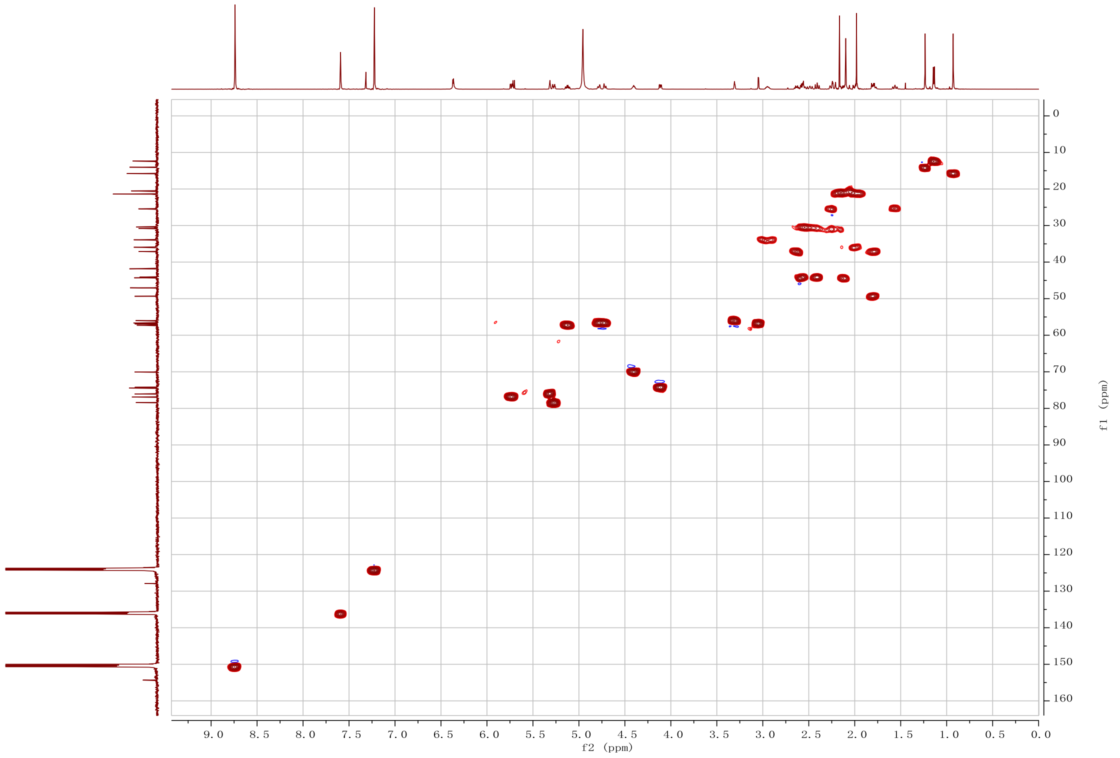

Figure S47. HSQC spectrum of chantriolide K (**6**) in C_5D_5N

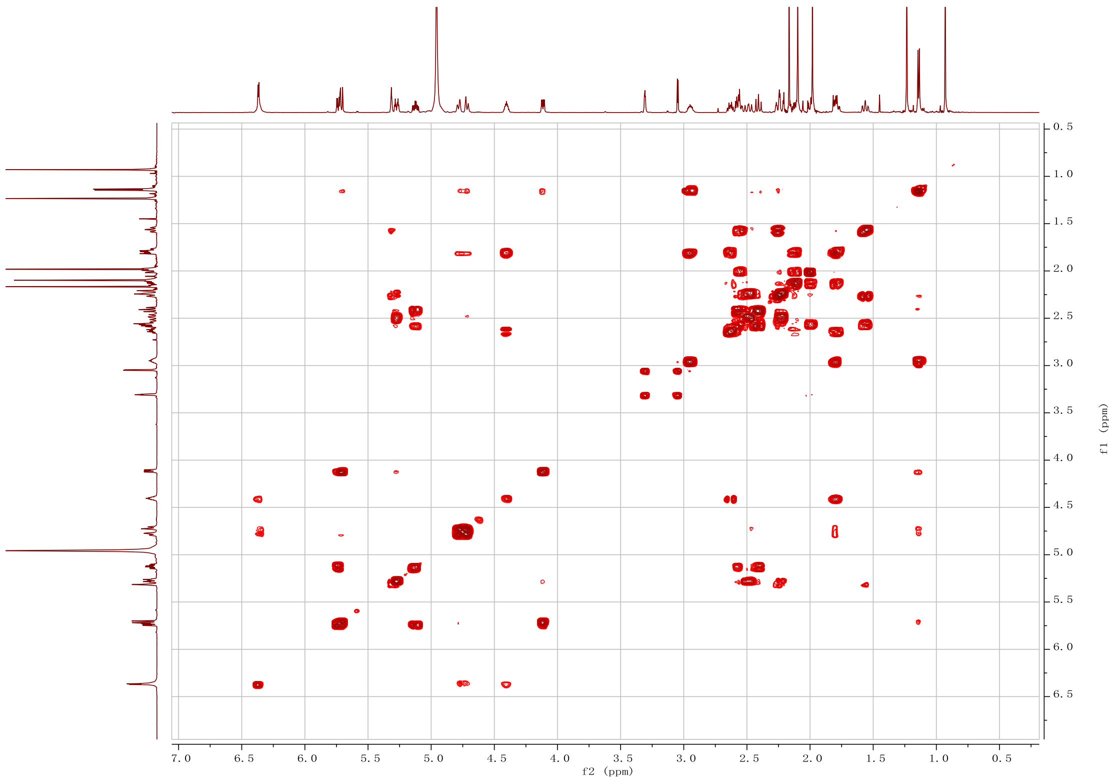

Figure S48. ^1H-^1H COSY spectrum of chantriolide K (**6**) in C_5D_5N

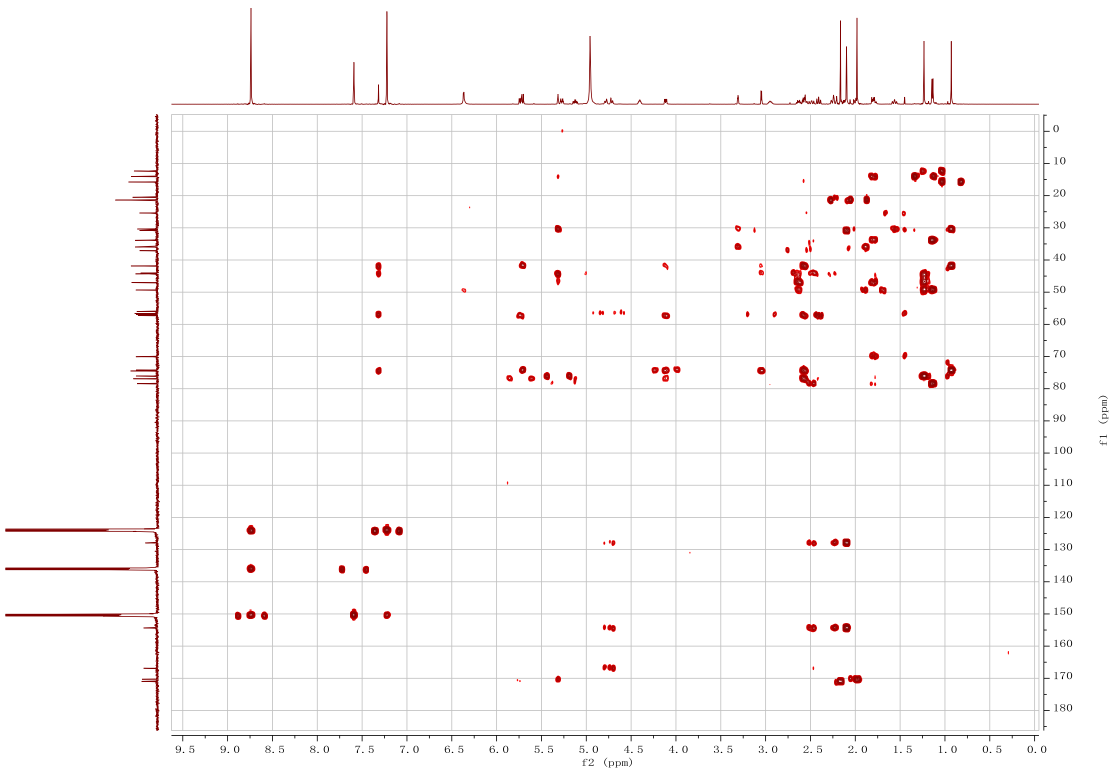

Figure S49. HMBC spectrum of chantriolide K (**6**) in C_5D_5N

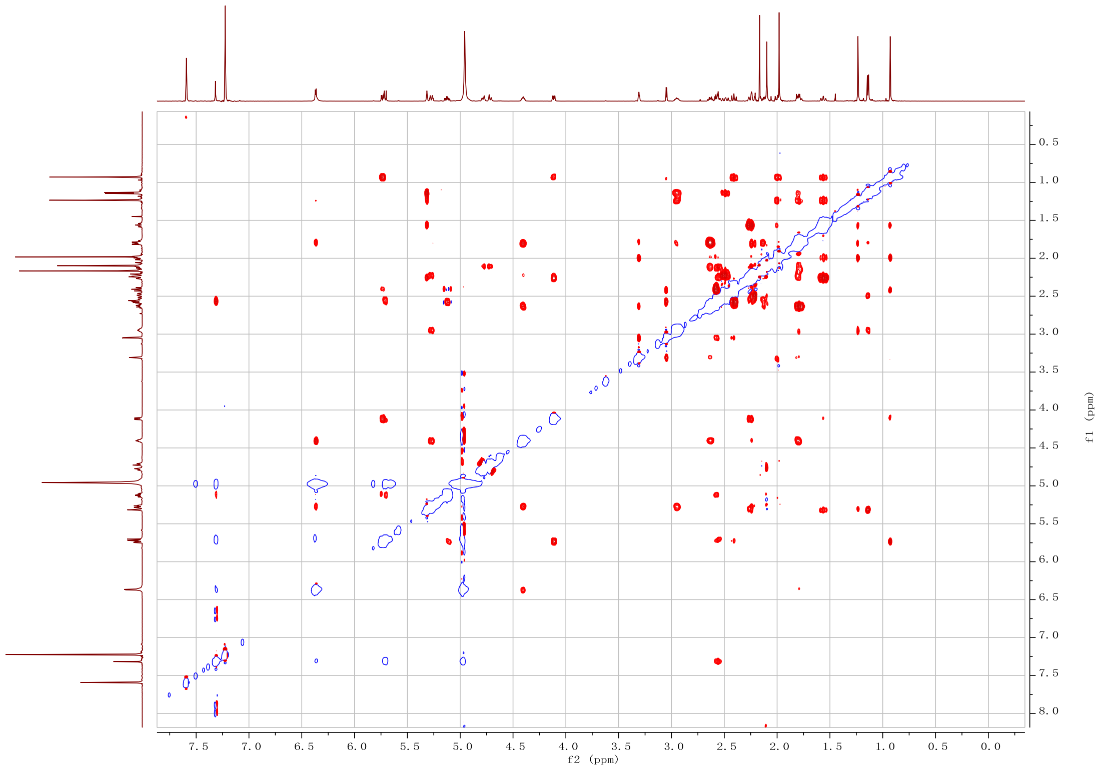

Figure S50. ROESY spectrum of chantriolide K (**6**) in C_5D_5N

**Data Filename**	20171010_ESIH_YY_YY_172240.d	**Sample Name**	D3-JGS-B2K1E5C
**Sample Type**	Sample	**Position**	P2-A3
**Instrument Name**	Agilent G6520 Q-TOF	**Acq Method**	20160322_MS_ESIH_POS_1min.m
**Acquired Time**	10/10/2017 10:34:22 AM	**IRM Calibration Status**	Success
**DA Method**	small molecular data analysis method.m	**Comment**	ESIH by ZZY

User Spectra

Spectrum Source	Fragmentor Voltage	Collision Energy	Ionization Mode
	110	0	ESI

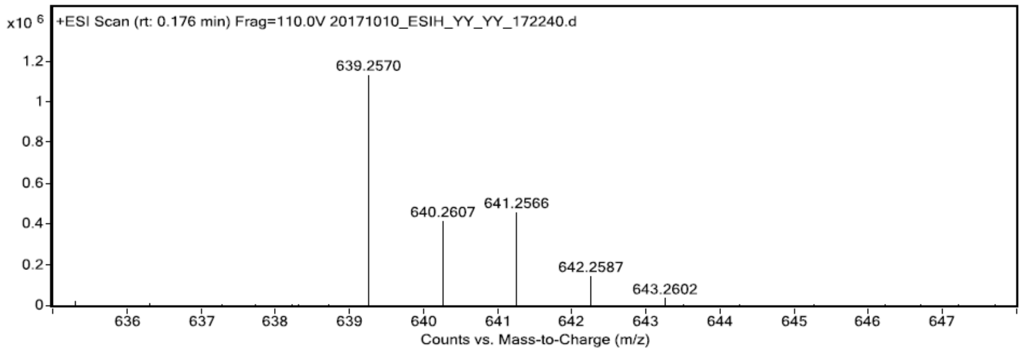

Formula Calculator Results

m/z	Calc m/z	Diff (mDa)	Diff (ppm)	Ion Formula	Ion
639.257	639.2567	-0.29	-0.45	C32 H44 Cl O11	(M+H)+

Figure S51. HRESIMS spectrum of chantriolide L (**7**)

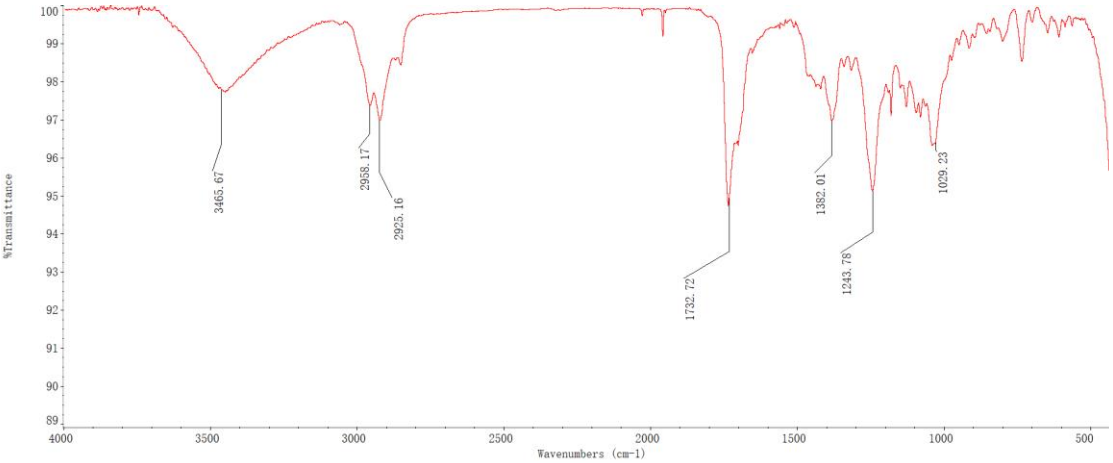

Figure S52. IR spectrum of chantriolide L (**7**)

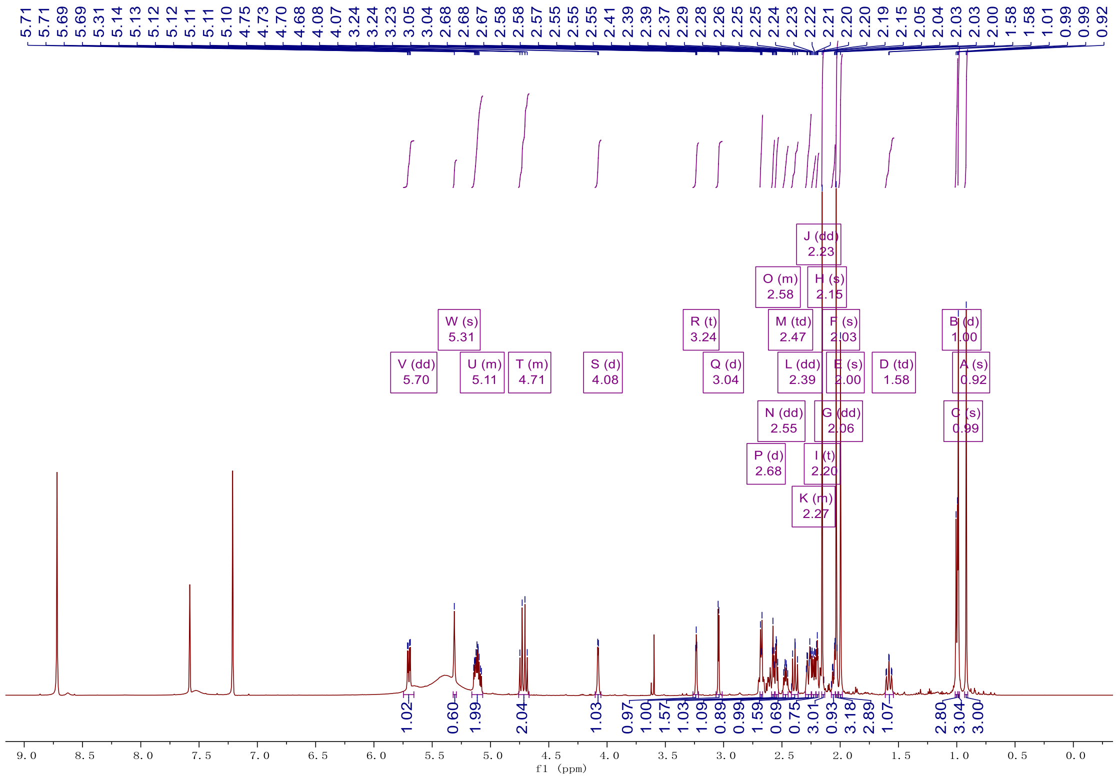

Figure S53. ^{1}H NMR (600 MHz) spectrum of chantriolide L (**7**) in C_5D_5N

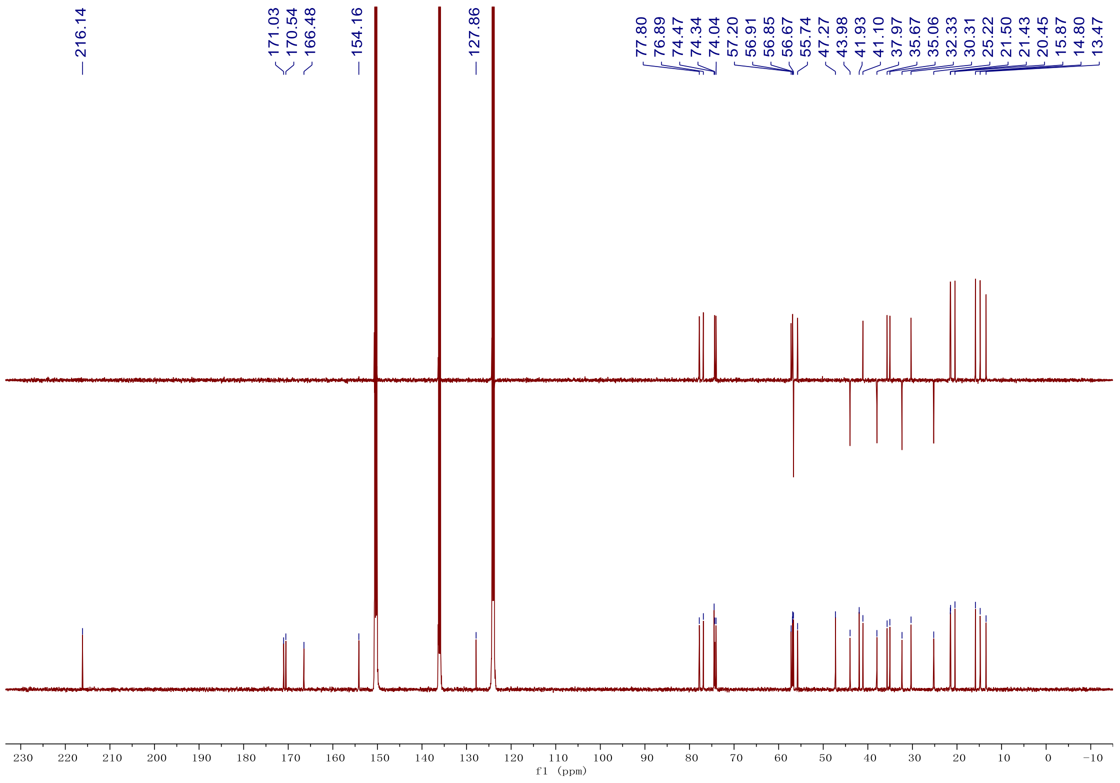

Figure S54. ^{13}C and DEPT-135 NMR (125 MHz) spectra of chantriolide L (**7**) in C_5D_5N

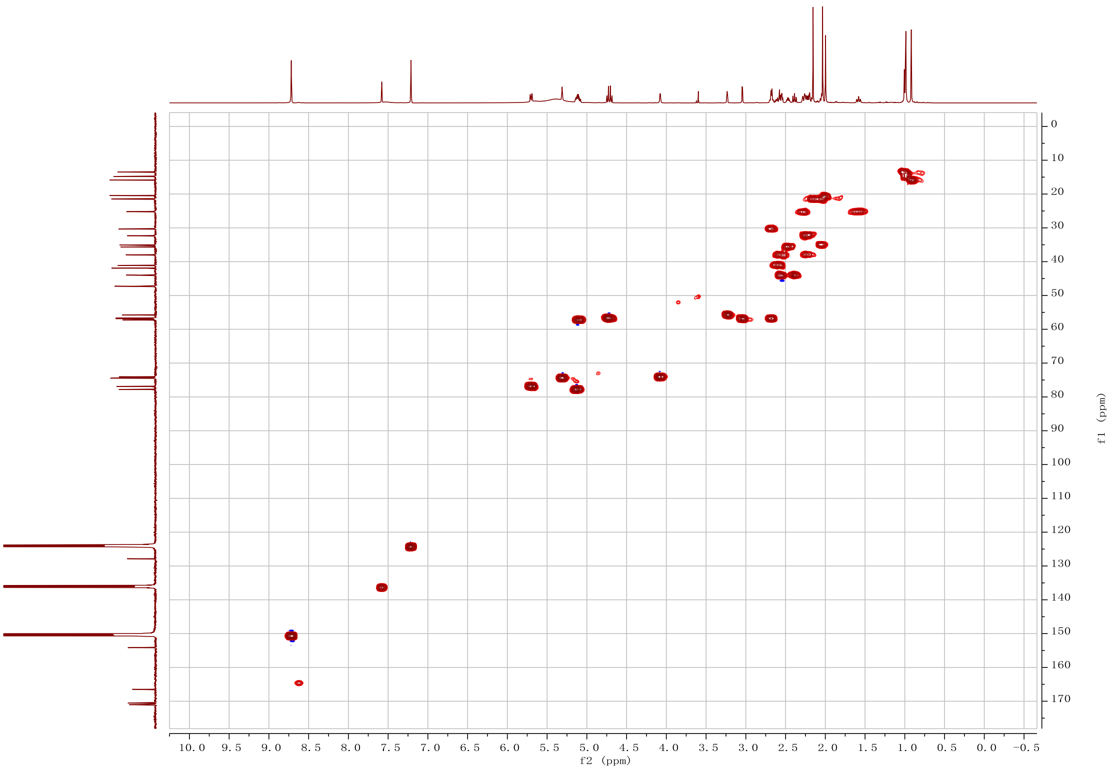

Figure S55. HSQC spectrum of chantriolide L (**7**) in C_5D_5N

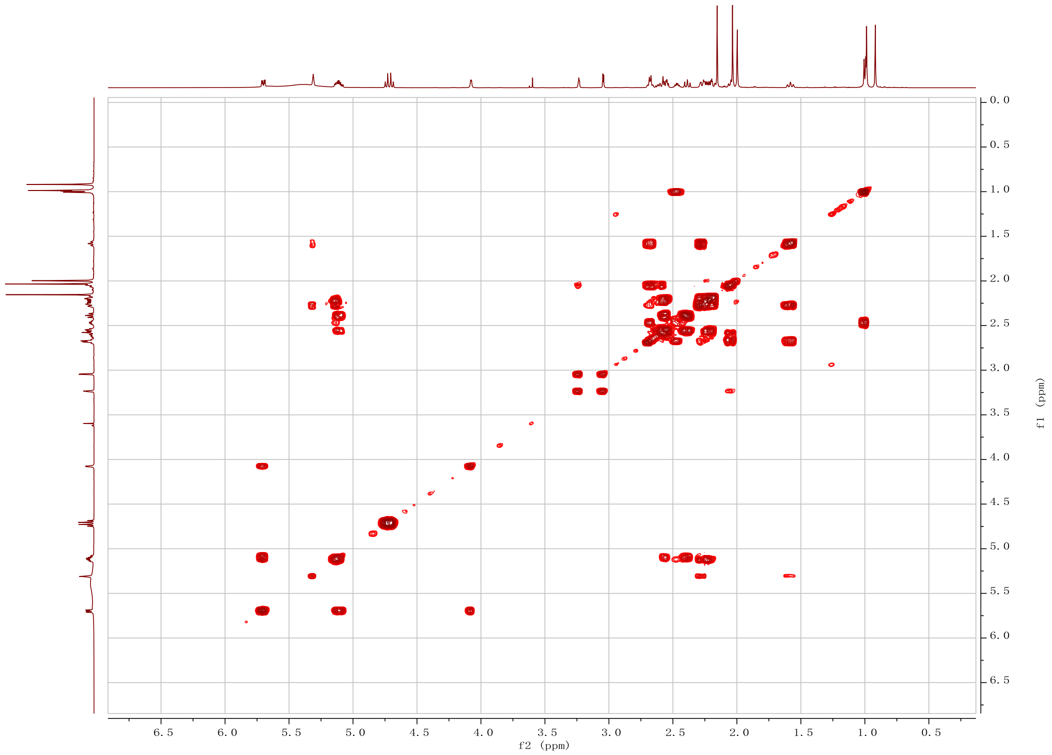

Figure S56. ^1H-^1H COSY spectrum of chantriolide L (**7**) in C_5D_5N

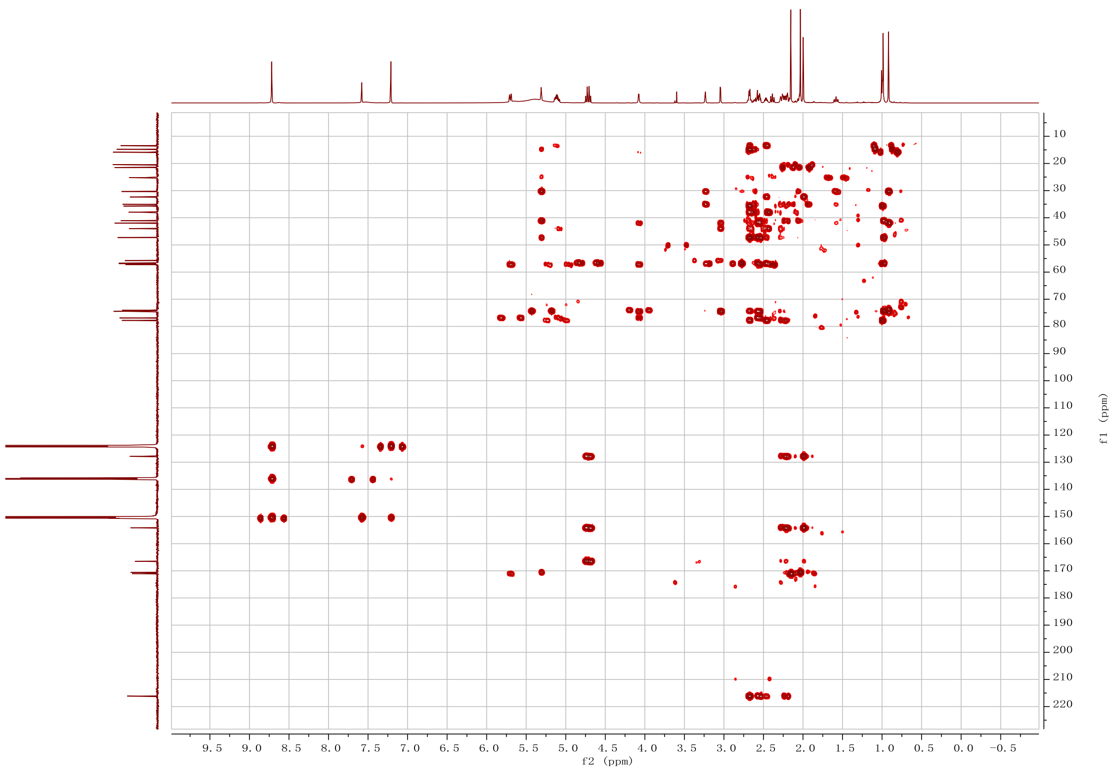

Figure S57. HMBC spectrum of chantriolide L (**7**) in C_5D_5N

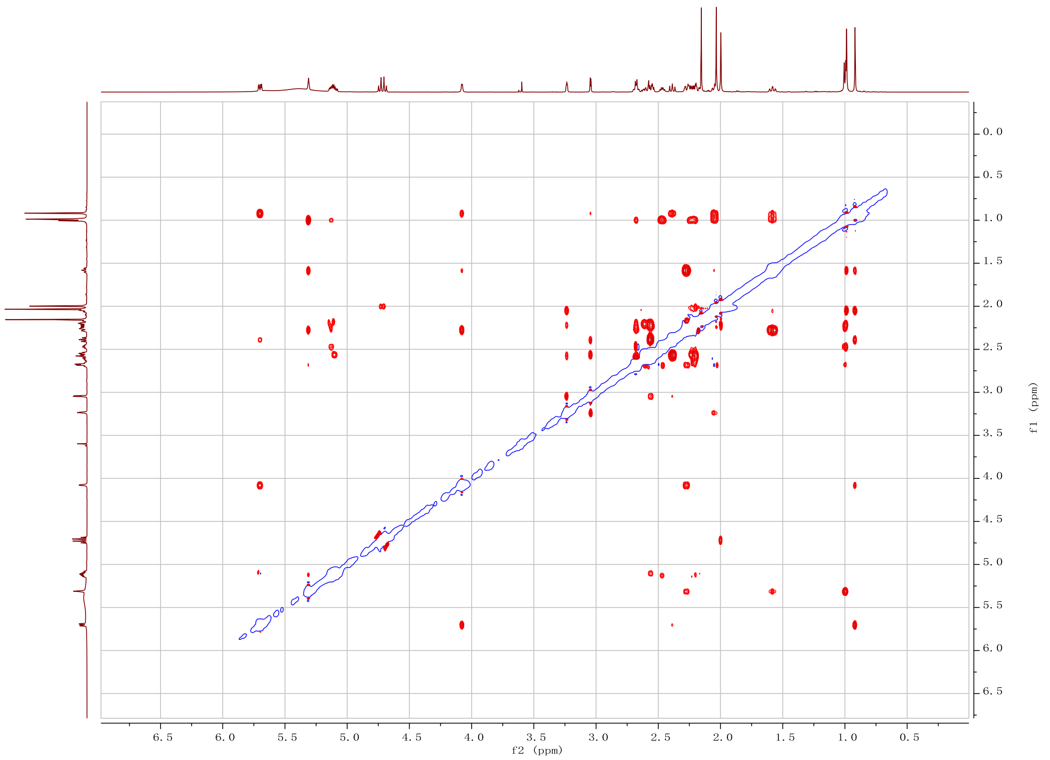

Figure S58. ROESY spectrum of chantriolide L (**7**) in C_5D_5N

**Data Filename**	20171010_ESIH_YY_YY_172239.d	**Sample Name**	D3-JGS-B2K1E5A
**Sample Type**	Sample	**Position**	P2-A2
**Instrument Name**	Agilent G6520 Q-TOF	**Acq Method**	20160322_MS_ESIH_POS_1min.m
**Acquired Time**	10/10/2017 10:32:29 AM	**IRM Calibration Status**	Success
**DA Method**	small molecular data analysis method.m	**Comment**	ESIH by ZZY

User Spectra

**Spectrum Source**	**Fragmentor Voltage**	**Collision Energy**	**Ionization Mode**
	110	0	ESI

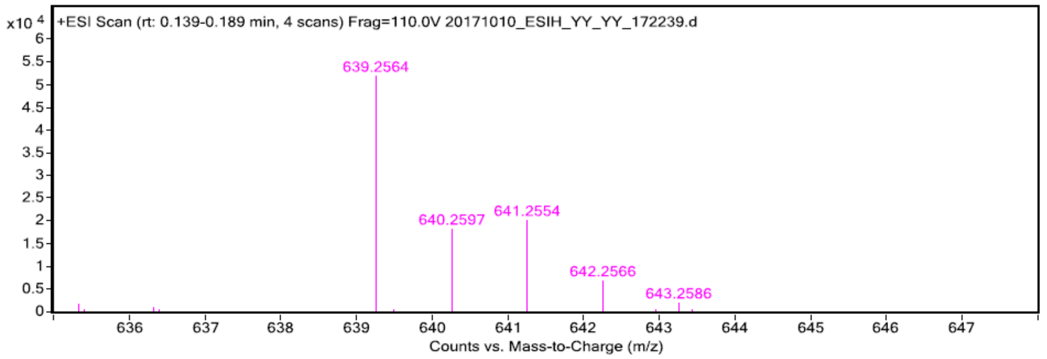

Formula Calculator Results

m/z	Calc m/z	Diff (mDa)	Diff (ppm)	Ion Formula	Ion
639.2564	639.2567	0.22	0.35	C32 H44 Cl O11	(M+H)+

Figure S59. HRESIMS spectrum of chantriolide M (**8**)

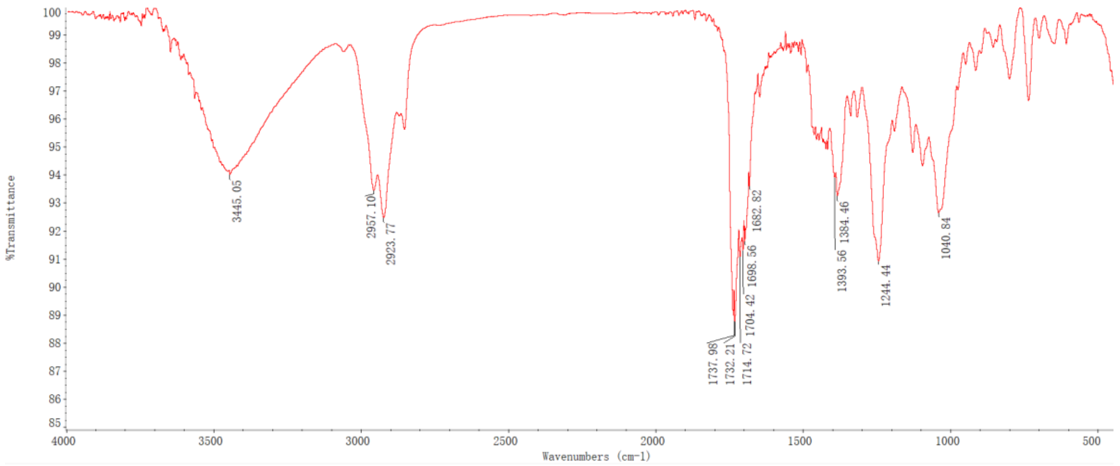

Figure S60. IR spectrum of chantriolide M (**8**)

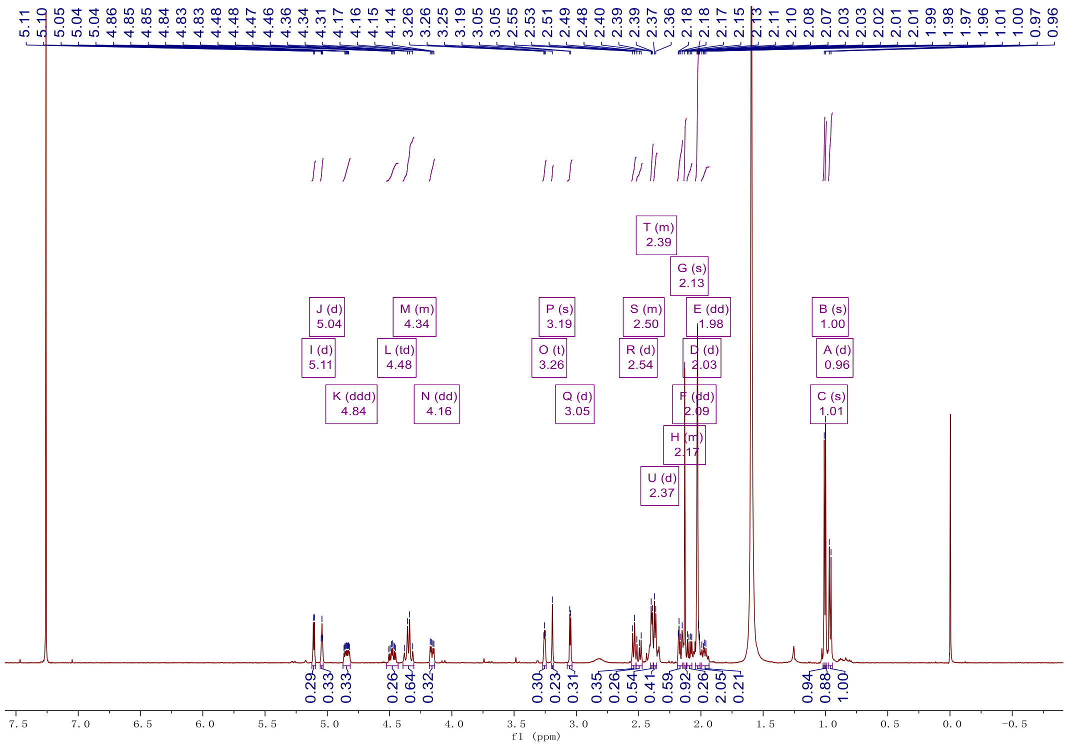

Figure S61. ^{1}H NMR (500 MHz) spectrum of chantriolide M (**8**) in $CDCl_3$

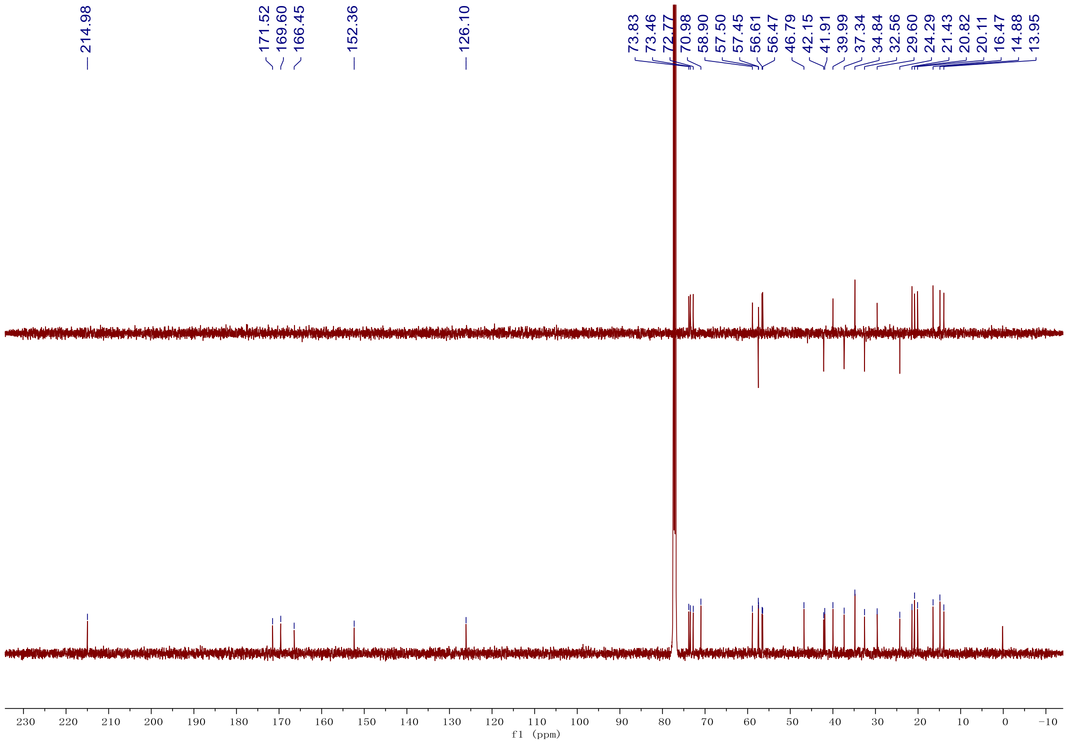

Figure S62. ^{13}C and DEPT-135 NMR (125 MHz) spectra of chantriolide M (**8**) in $CDCl_3$

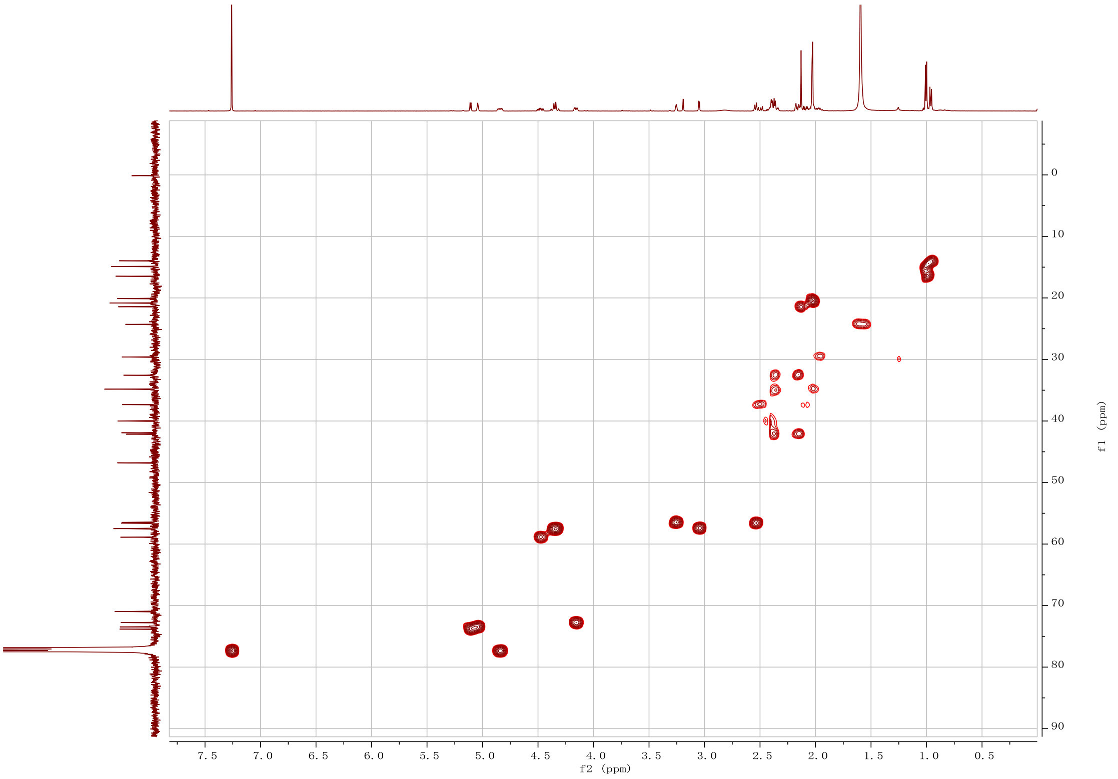

Figure S63. HSQC spectrum of chantriolide M (**8**) in $CDCl_3$

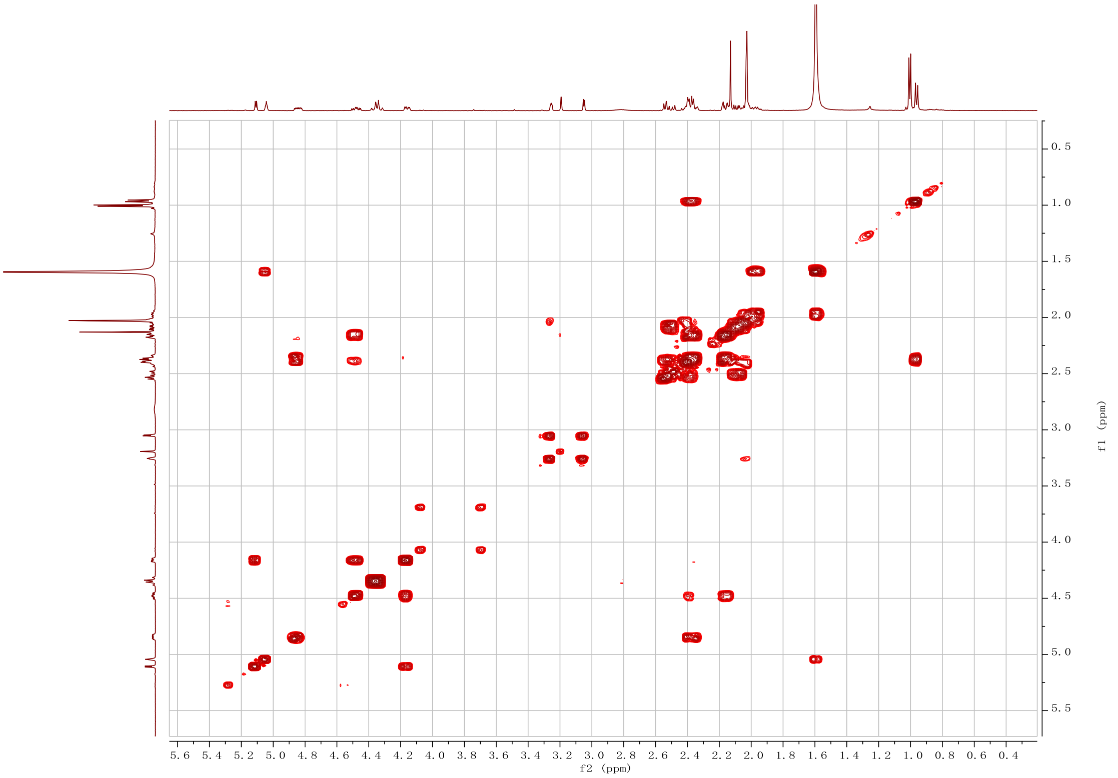

Figure S64. ^1H-^1H COSY spectrum of chantriolide M (**8**) in $CDCl_3$

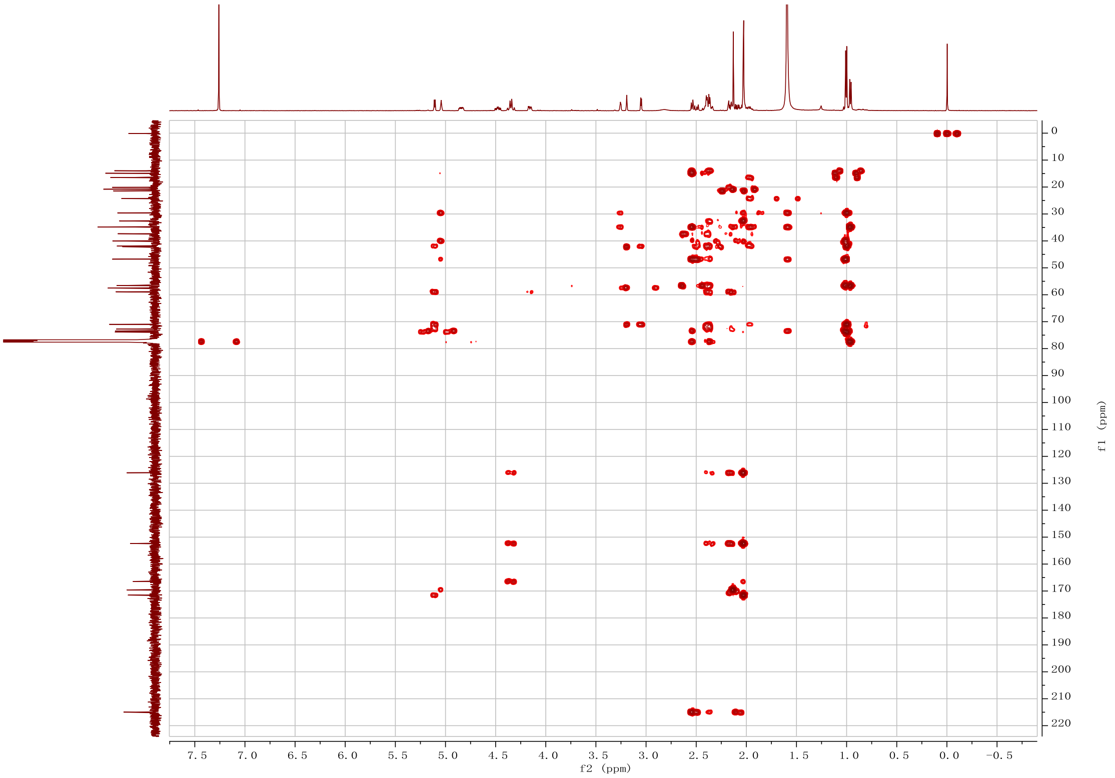

Figure S65. HMBC spectrum of chantriolide M (**8**) in $CDCl_3$

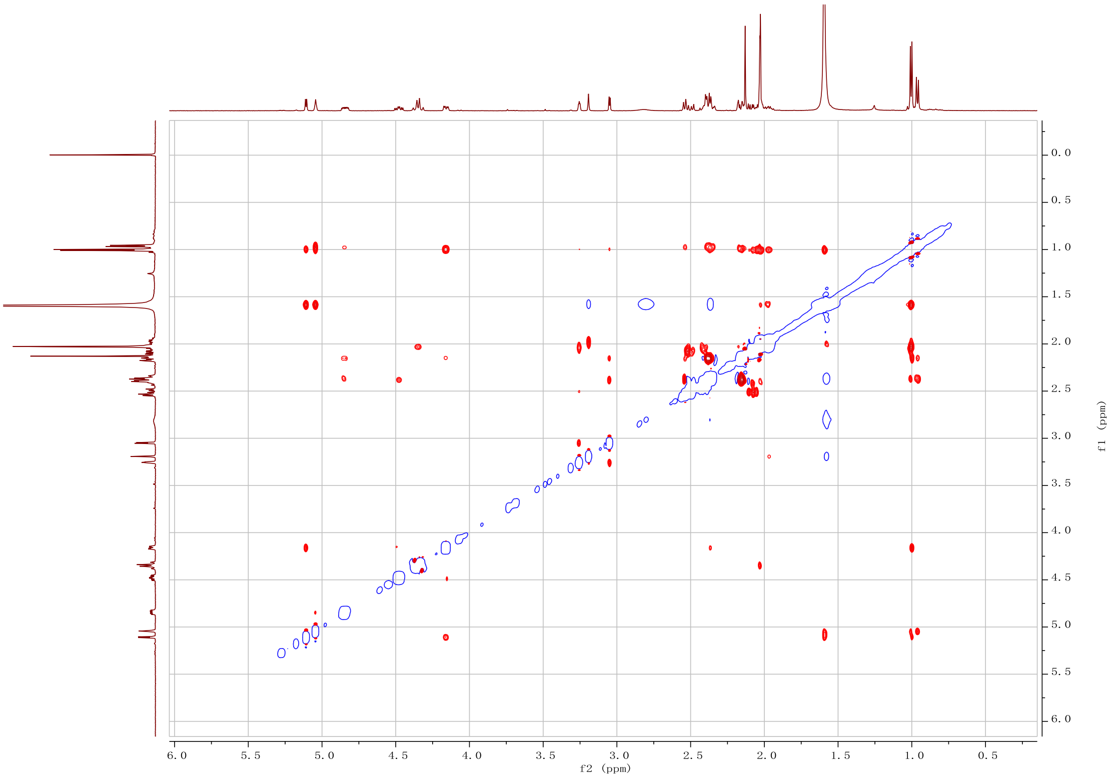

Figure S66. ROESY spectrum of chantriolide M (**8**) in $CDCl_3$

Elemental Composition Report

Tolerance = 5.0 PPM / DBE: min = -1.5, max = 50.0
Element prediction: Off
Number of isotope peaks used for i-FIT = 3

Monoisotopic Mass, Even Electron Ions
102 formula(e) evaluated with 1 results within limits (up to 50 best isotopic matches for each mass)
Elements Used:
C: 0-34 H: 0-200 O: 0-30 Na: 1-1

Minimum:	80.00				-1.5				
Maximum:	100.00		5.0	5.0	50.0				
Mass	RA	Calc. Mass	mDa	PPM	DBE	i-FIT	Norm	Conf(%)	Formula
499.3026	100.00	499.3036	-1.0	-2.0	6.5	379.1	n/a	n/a	C28 H44 O6 Na

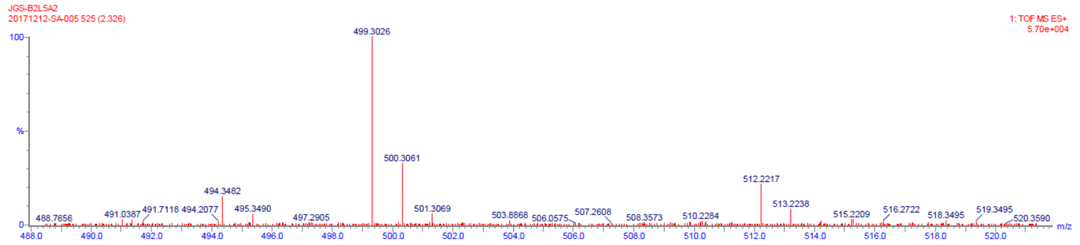

Figure S67. HRESIMS spectrum of chantriolide N (**9**)

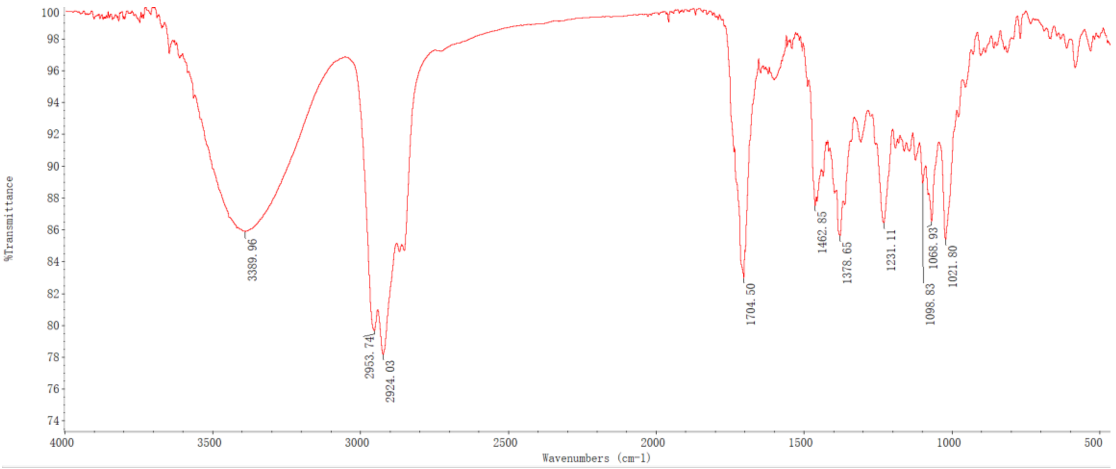

Figure S68. IR spectrum of chantriolide N (**9**)

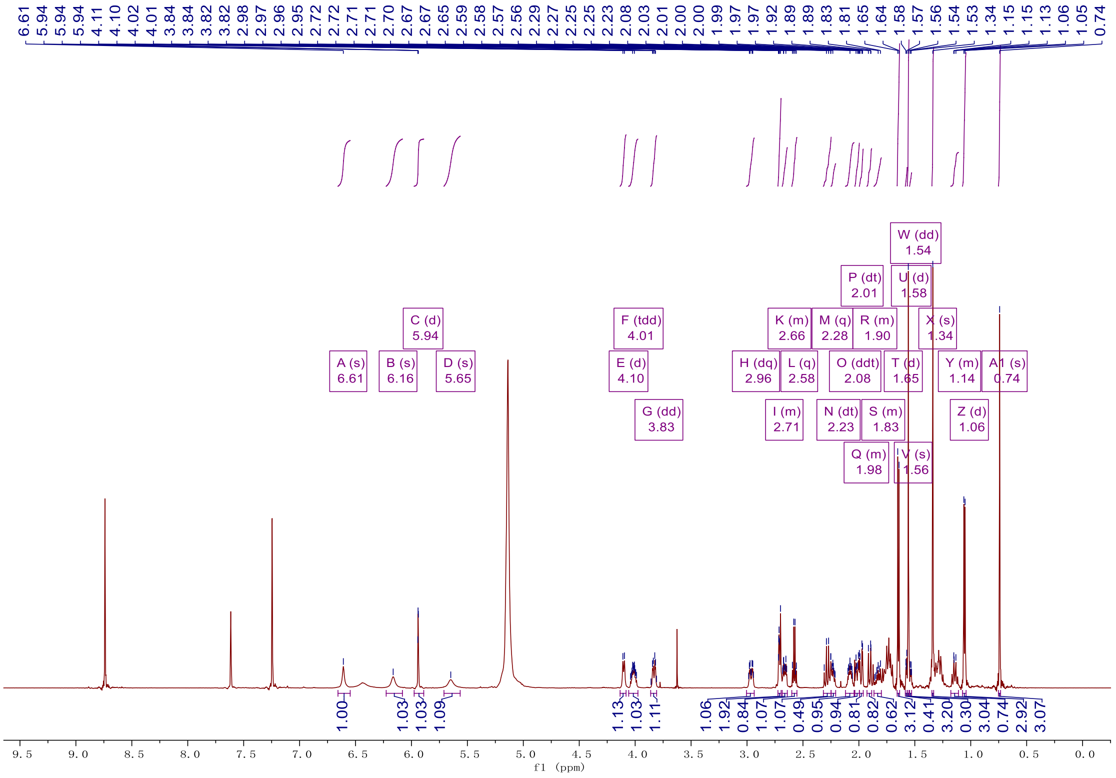

Figure S69. ^{1}H NMR (600 MHz) spectrum of chantriolide N (**9**) in C_5D_5N

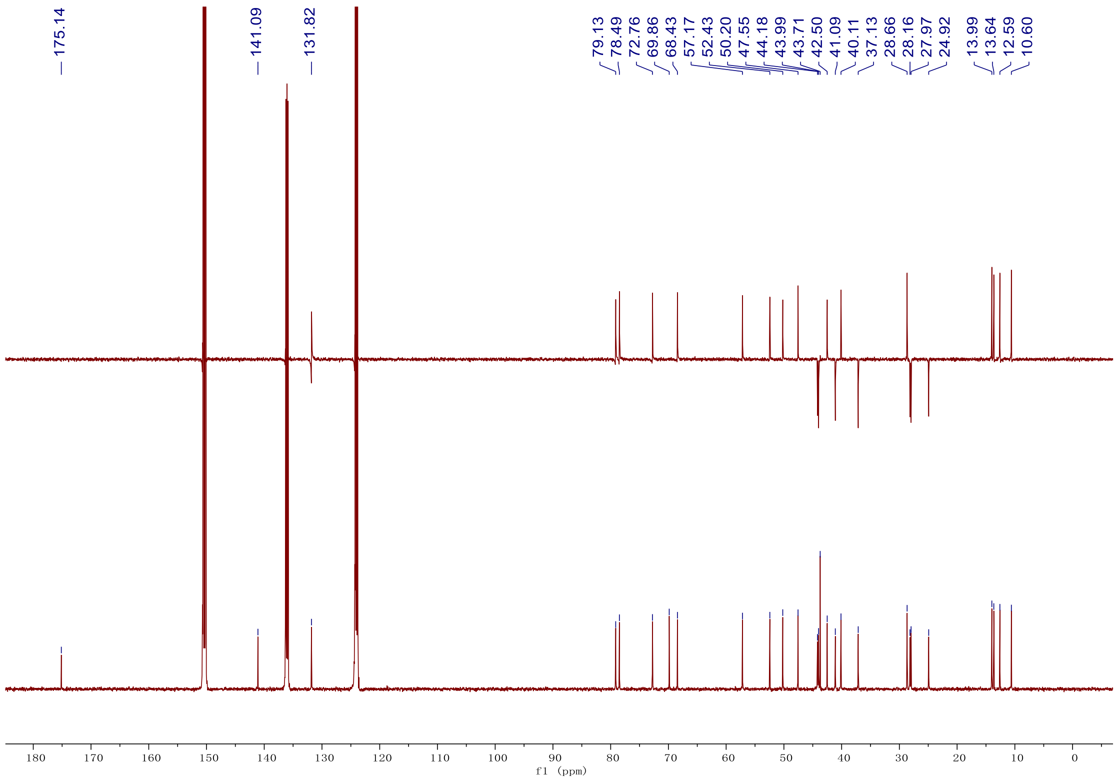

Figure S70. ^{13}C and DEPT-135 NMR (125 MHz) spectra of chantriolide N (**9**) in C_5D_5N

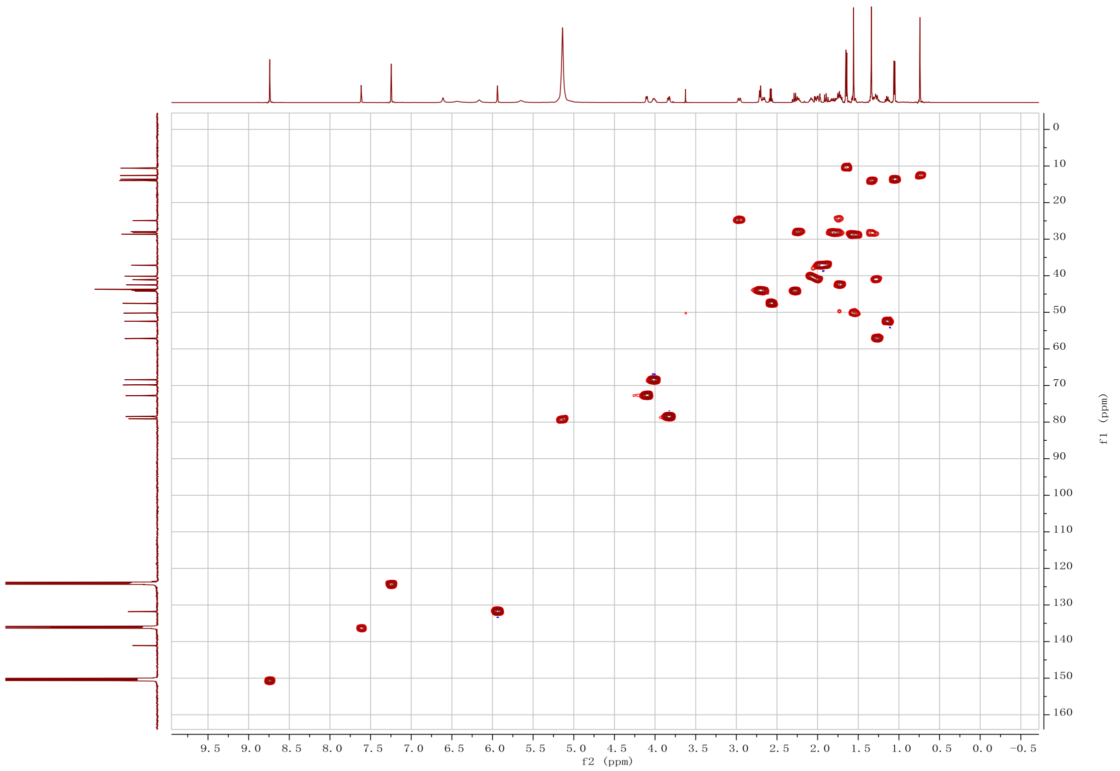

Figure S71. HSQC spectrum of chantriolide N (**9**) in C_5D_5N

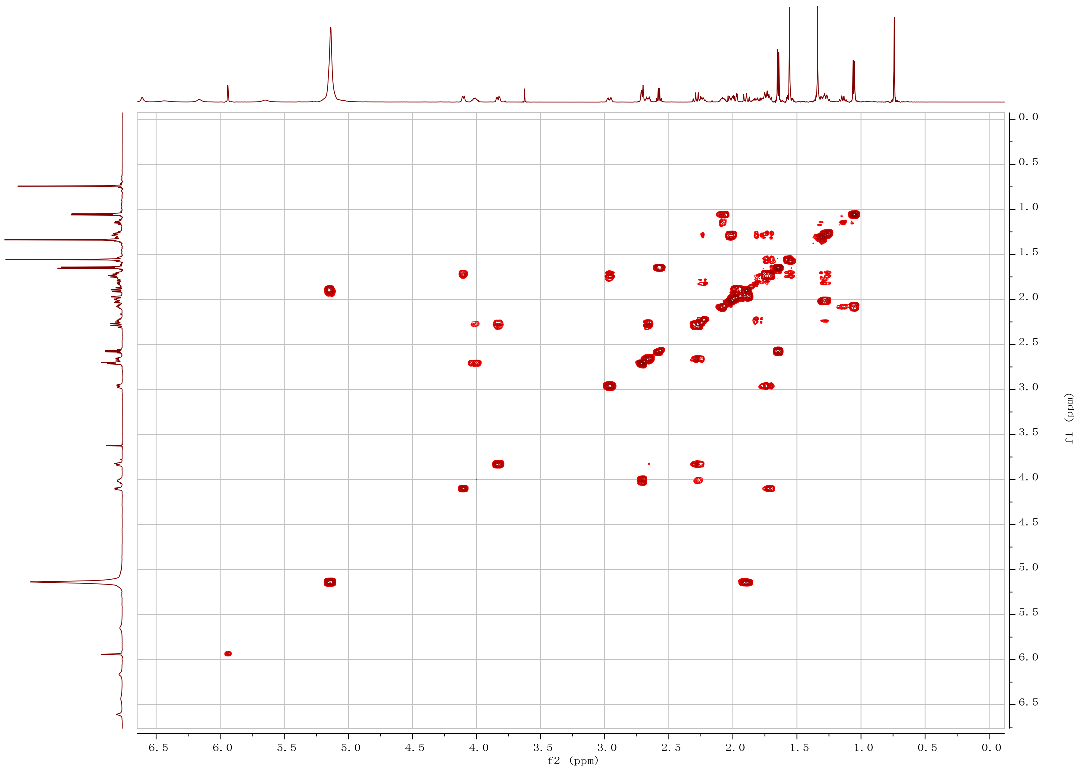

Figure S72. ^1H-^1H COSY spectrum of chantriolide N (**9**) in C_5D_5N

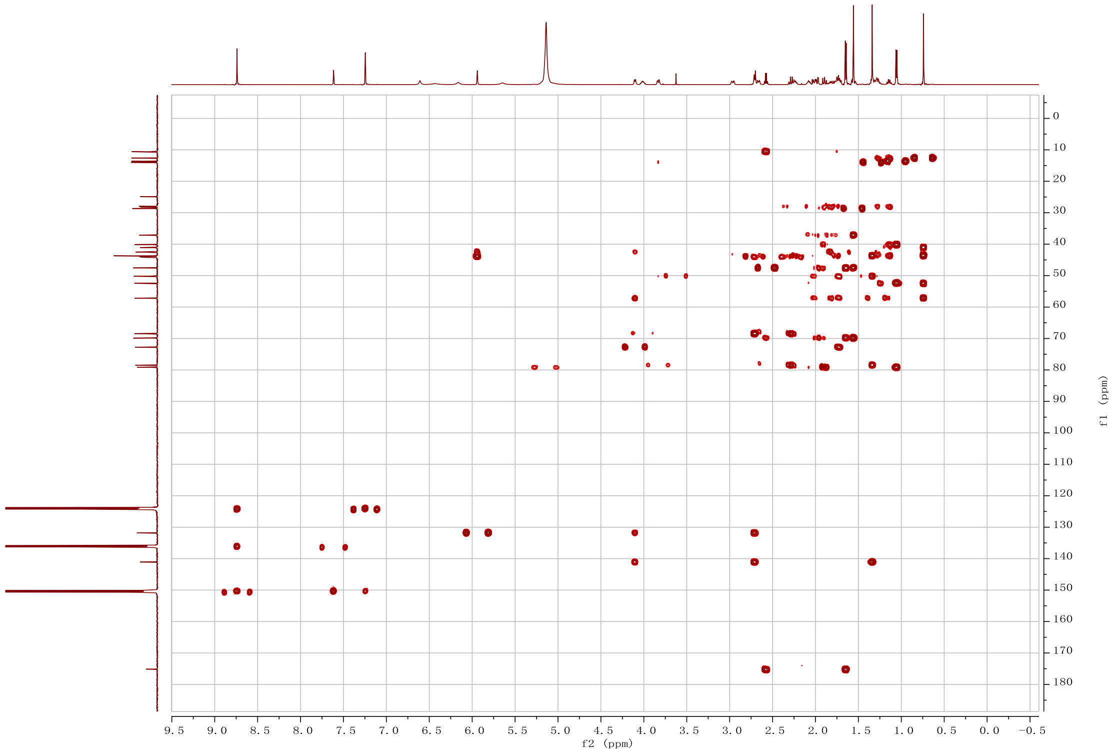

Figure S73. HMBC spectrum of chantriolide N (**9**) in C_5D_5N

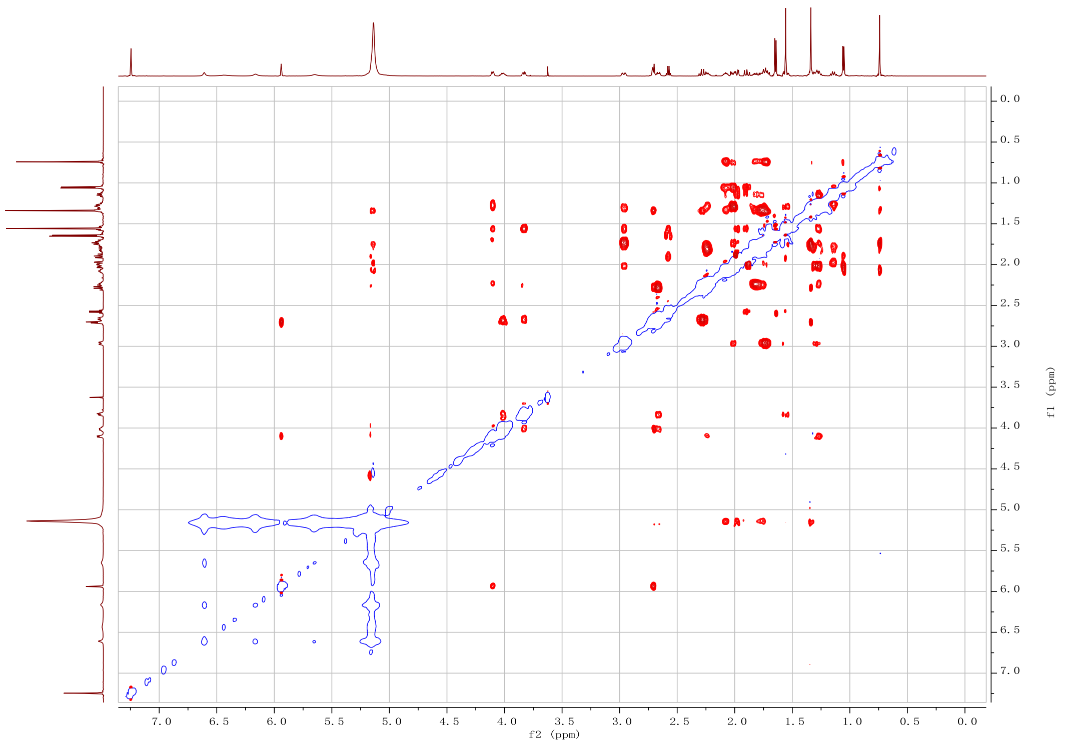

Figure S74. ROESY spectrum of chantriolide N (**9**) in C_5D_5N

Elemental Composition Report

Single Mass Analysis

Tolerance = 10.0 PPM / DBE: min = -1.5, max = 50.0

Element prediction: Off

Number of isotope peaks used for i-FIT = 3

Monoisotopic Mass, Even Electron Ions

22 formula(e) evaluated with 1 results within limits (up to 5 best isotopic matches for each mass)

Elements Used:

C: 20-30 H: 20-46 O: 0-15

Mass	Calc. Mass	mDa	PPM	DBE	i-FIT	Norm	Conf(%)	Formula
Minimum:				-1.5				
Maximum:		5.0	10.0	50.0				
493.3180	493.3165	1.5	3.0	6.5	354.6	n/a	n/a	C28 H45 O7

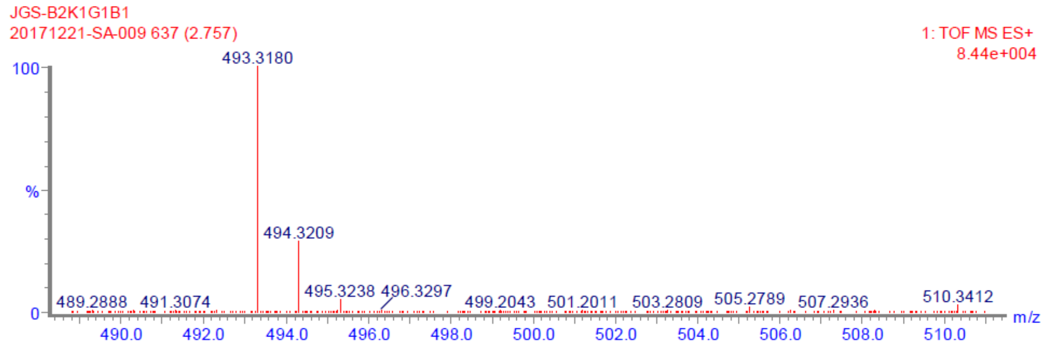

Figure S75. HRESIMS spectrum of chantriolide O (**10**)

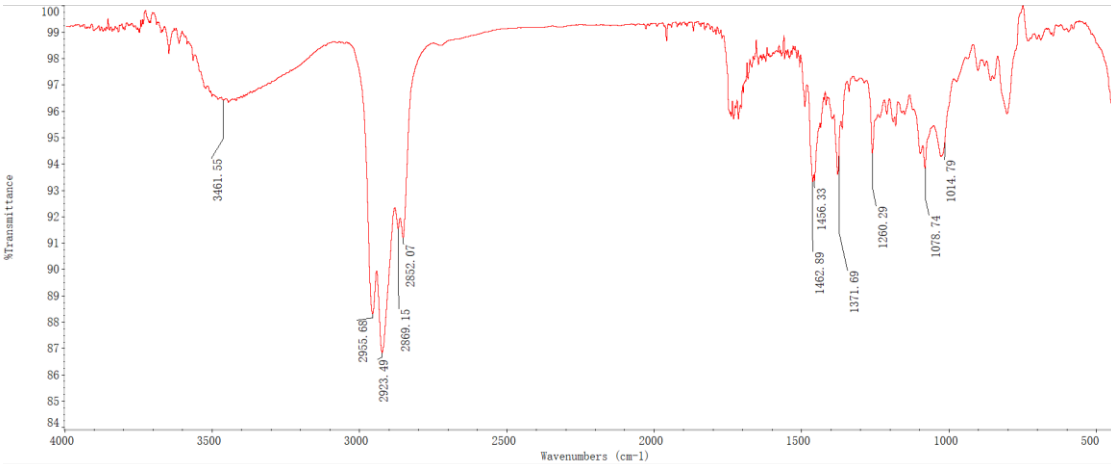

Figure S76. IR spectrum of chantriolide O (**10**)

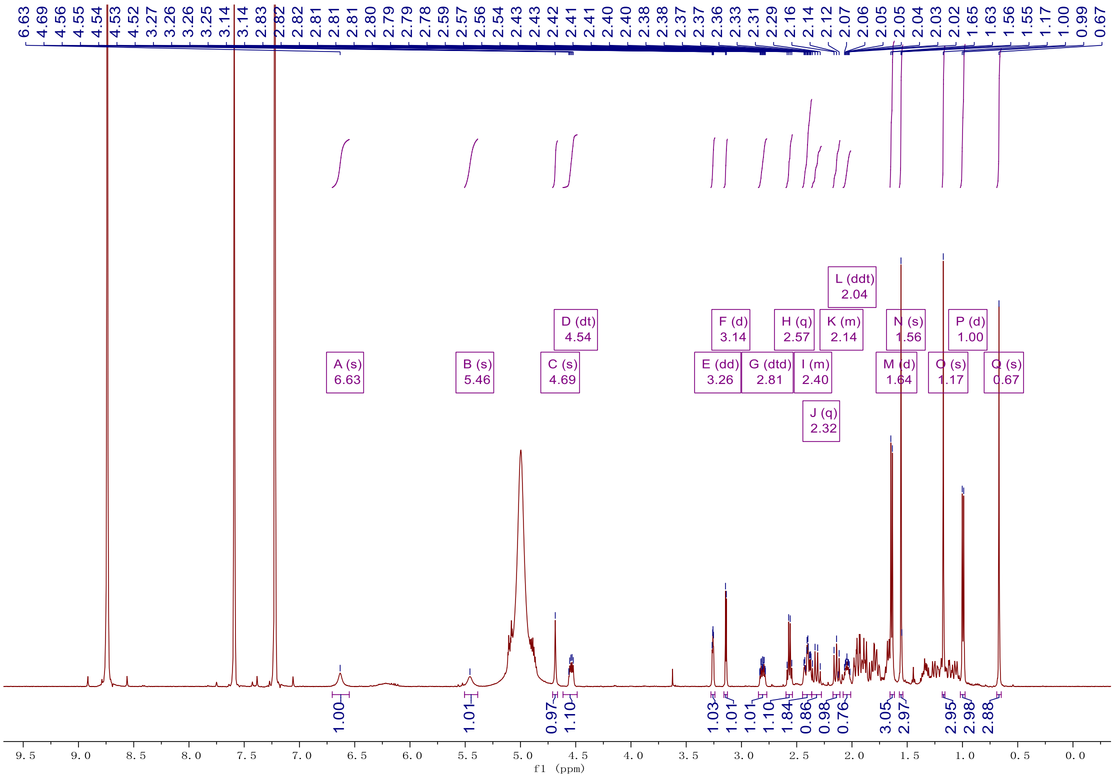

Figure S77. ^{1}H NMR (500 MHz) spectrum of chantriolide O (**10**) in C_5D_5N

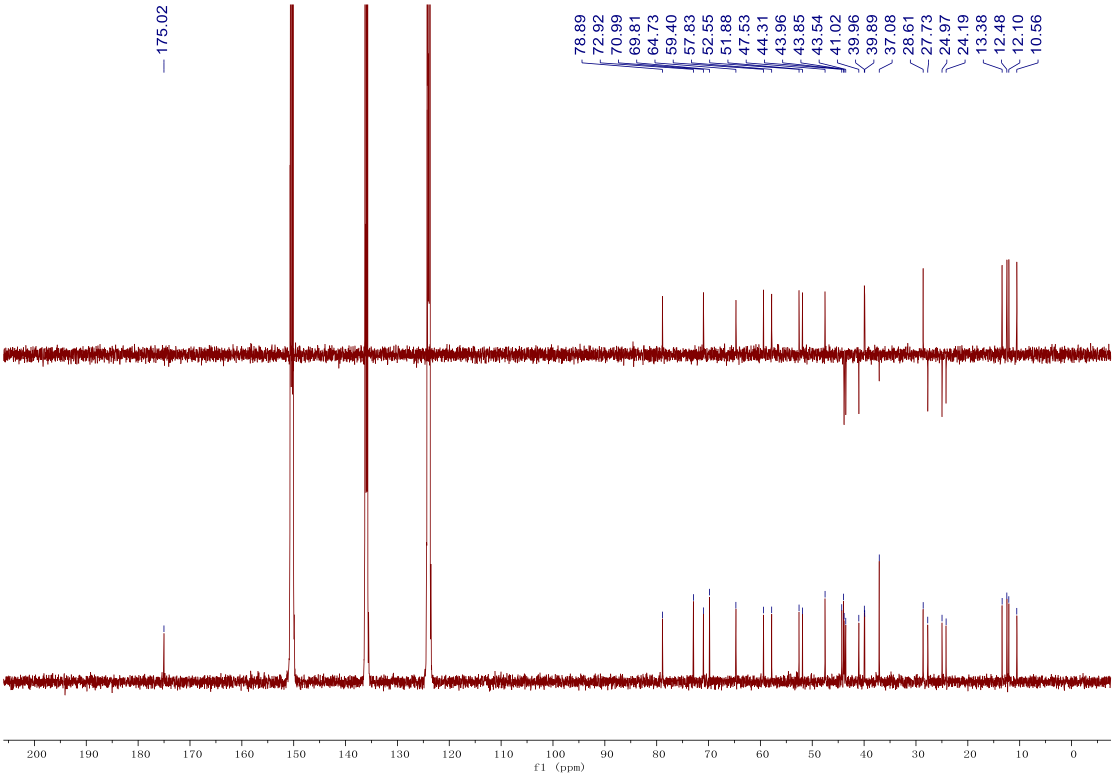

Figure S78. ^{13}C and DEPT-135 NMR (125 MHz) spectra of chantriolide O (**10**) in C_5D_5N

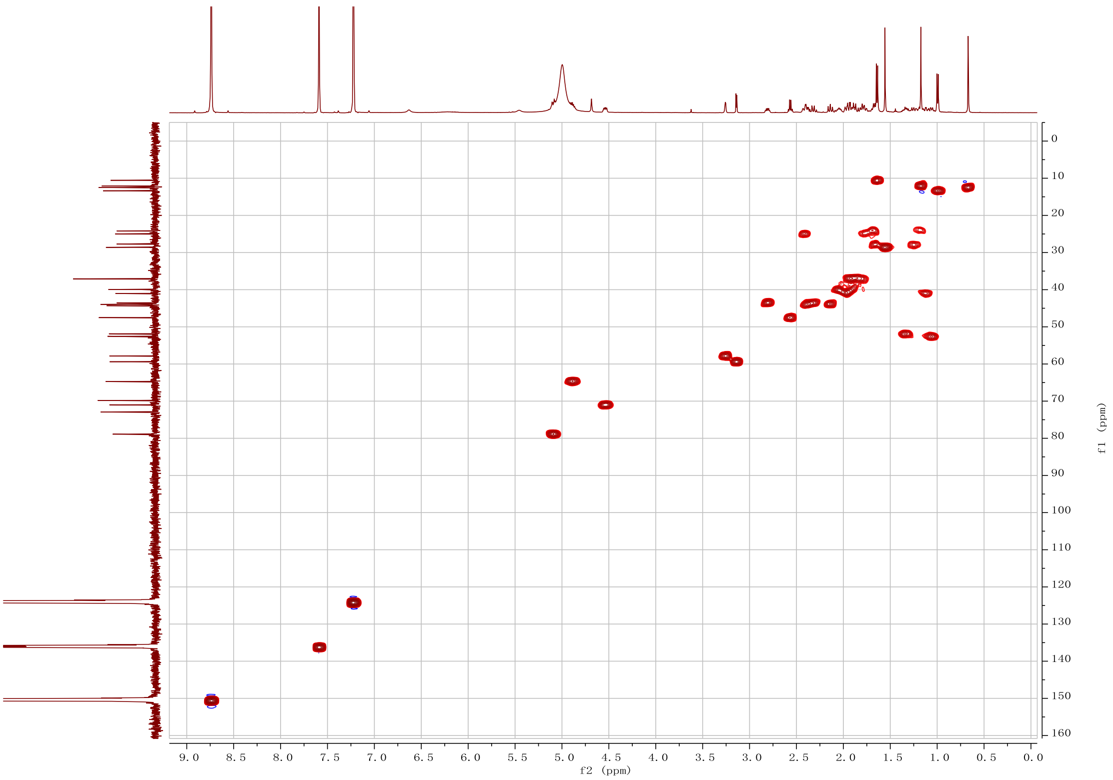

Figure S79. HSQC spectrum of chantriolide O (**10**) in C_5D_5N

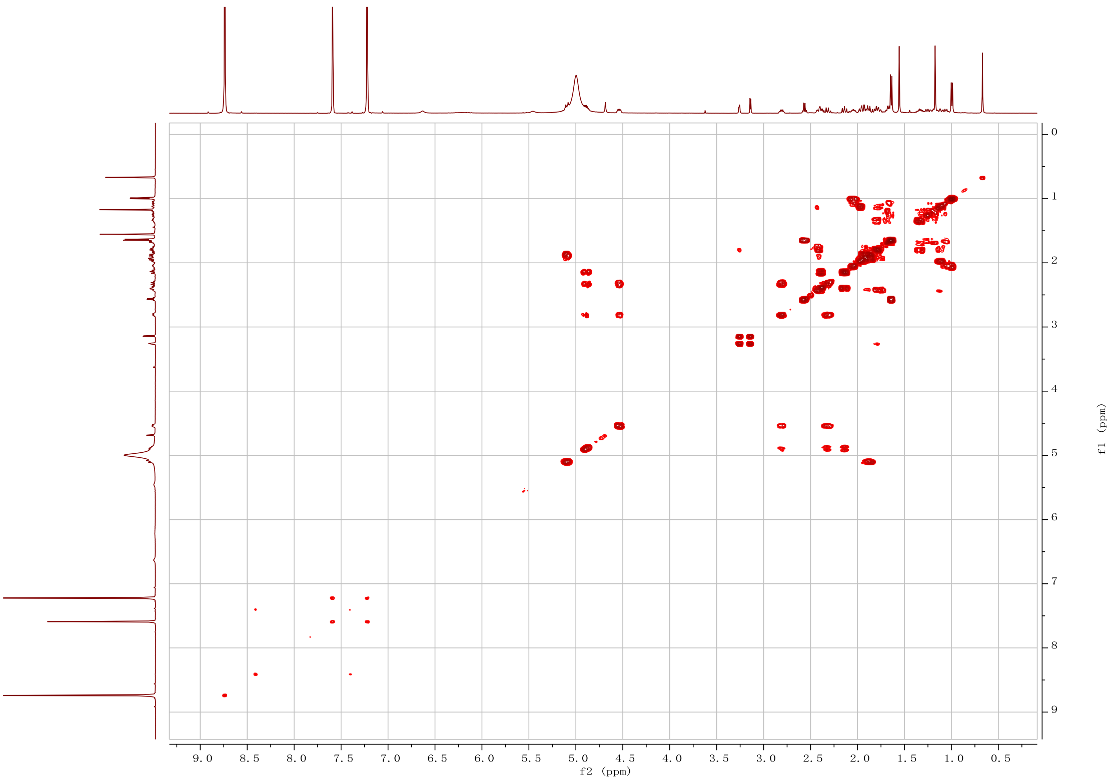

Figure S80. ^1H-^1H COSY spectrum of chantriolide O (**10**) in C_5D_5N

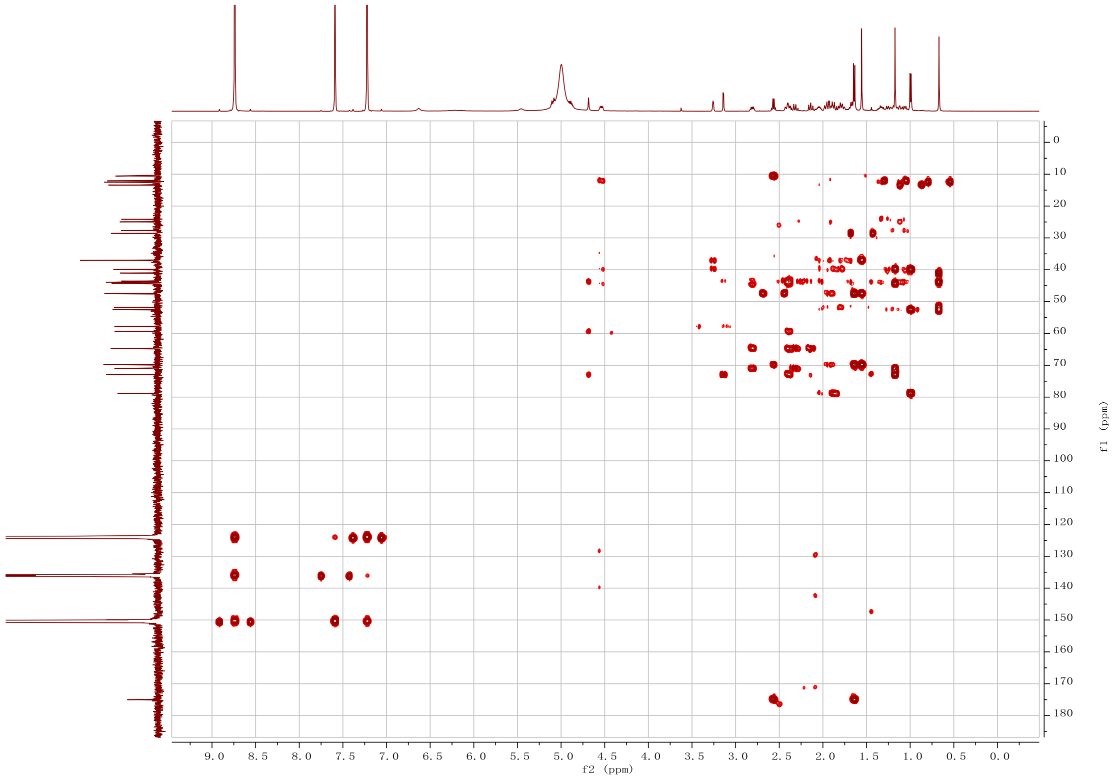

Figure S81. HMBC spectrum of chantriolide O (**10**) in C_5D_5N

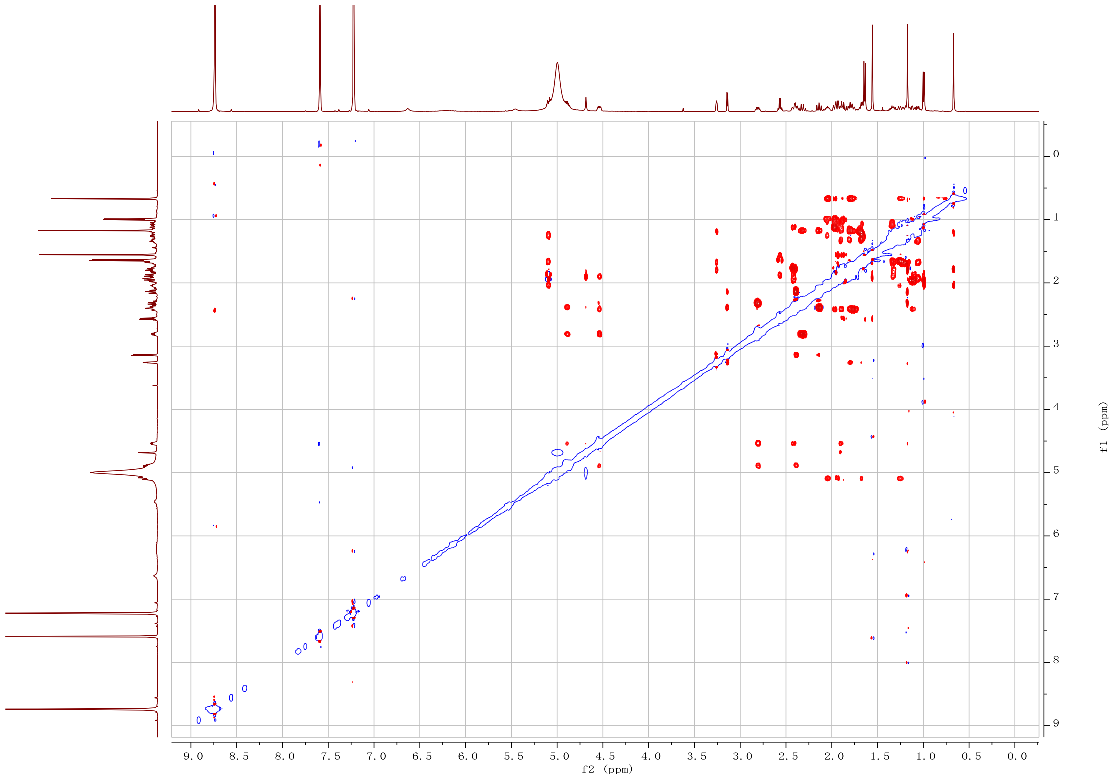

Figure S82. ROESY spectrum of chantriolide O (**10**) in C_5D_5N

Elemental Composition Report

Tolerance = 5.0 PPM / DBE: min = -1.5, max = 50.0
Element prediction: Off
Number of isotope peaks used for i-FIT = 3

Monoisotopic Mass, Even Electron Ions
208 formula(e) evaluated with 1 results within limits (up to 50 best isotopic matches for each mass)
Elements Used:
C: 0-34 H: 0-200 N: 0-1 O: 0-30

Minimum:	80.00				-1.5				
Maximum:	100.00		5.0	5.0	50.0				
Mass	RA	Calc. Mass	mDa	PPM	DBE	i-FIT	Norm	Conf(%)	Formula
508.3284	100.00	508.3274	1.0	2.0	6.5	583.2	n/a	n/a	C28 H46 N O7

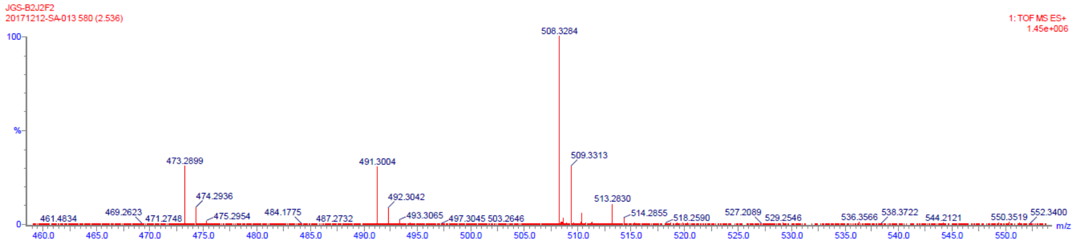

Figure S83. HRESIMS spectrum of chantriolide P (**11**)

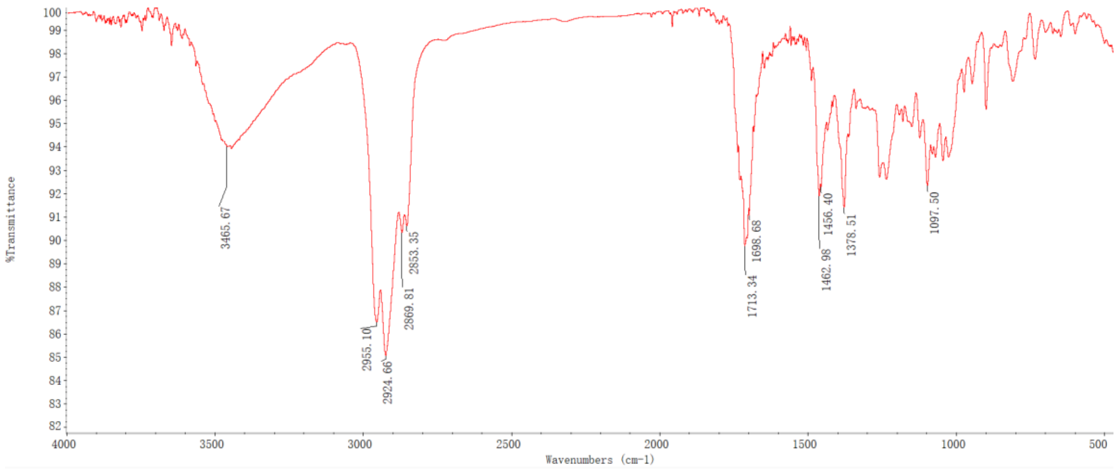

Figure S84. IR spectrum of chantriolide P (**11**)

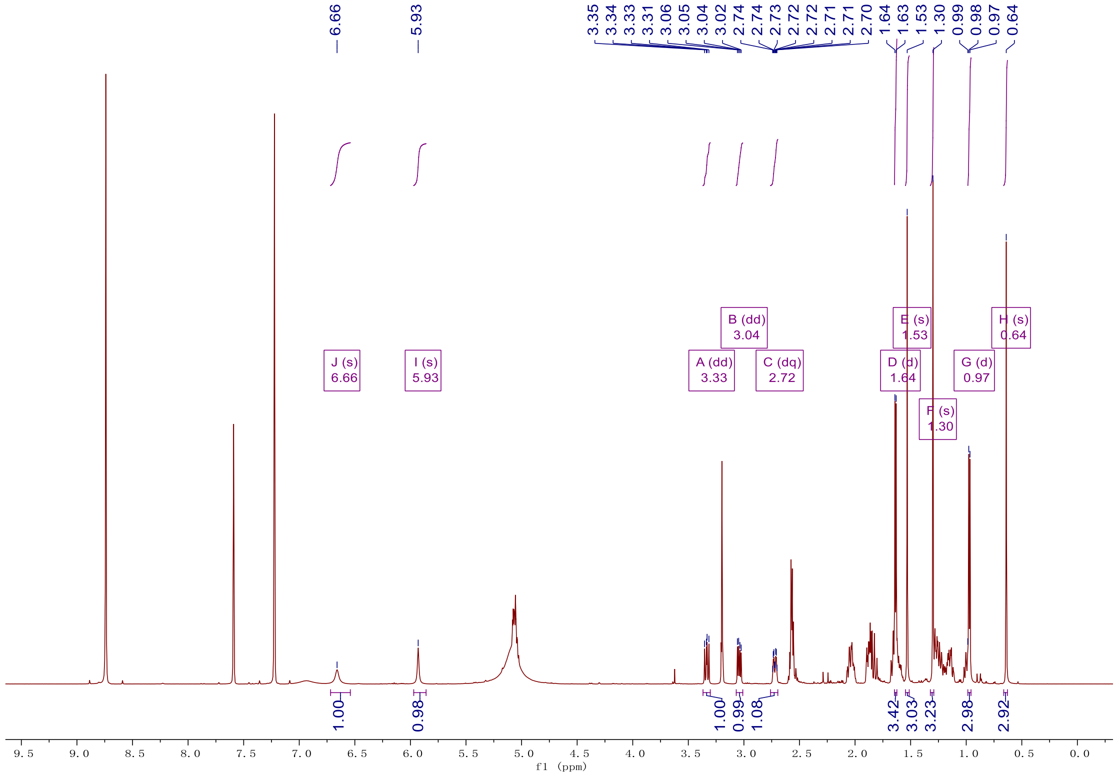

Figure S85. ^{1}H NMR (600 MHz) spectrum of chantriolide P (**11**) in C_5D_5N

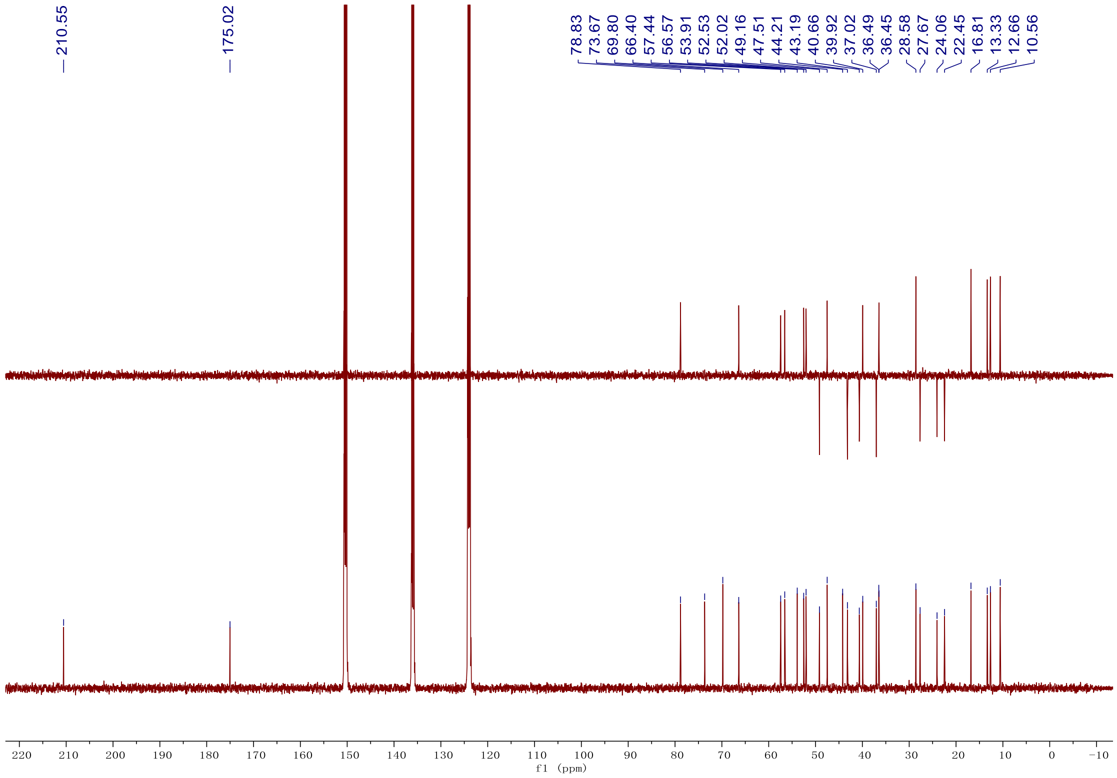

Figure S86. ^{13}C and DEPT-135 NMR (125 MHz) spectra of chantriolide P (**11**) in C_5D_5N

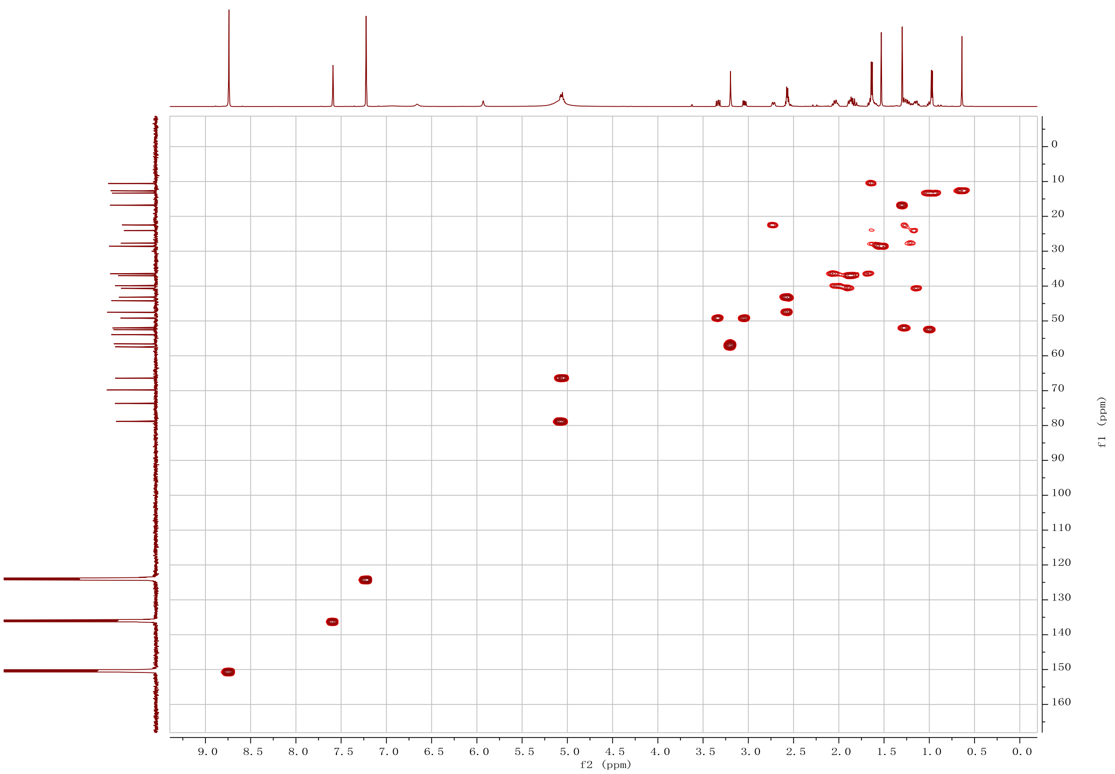

Figure S87. HSQC spectrum of chantriolide P (**11**) in C_5D_5N

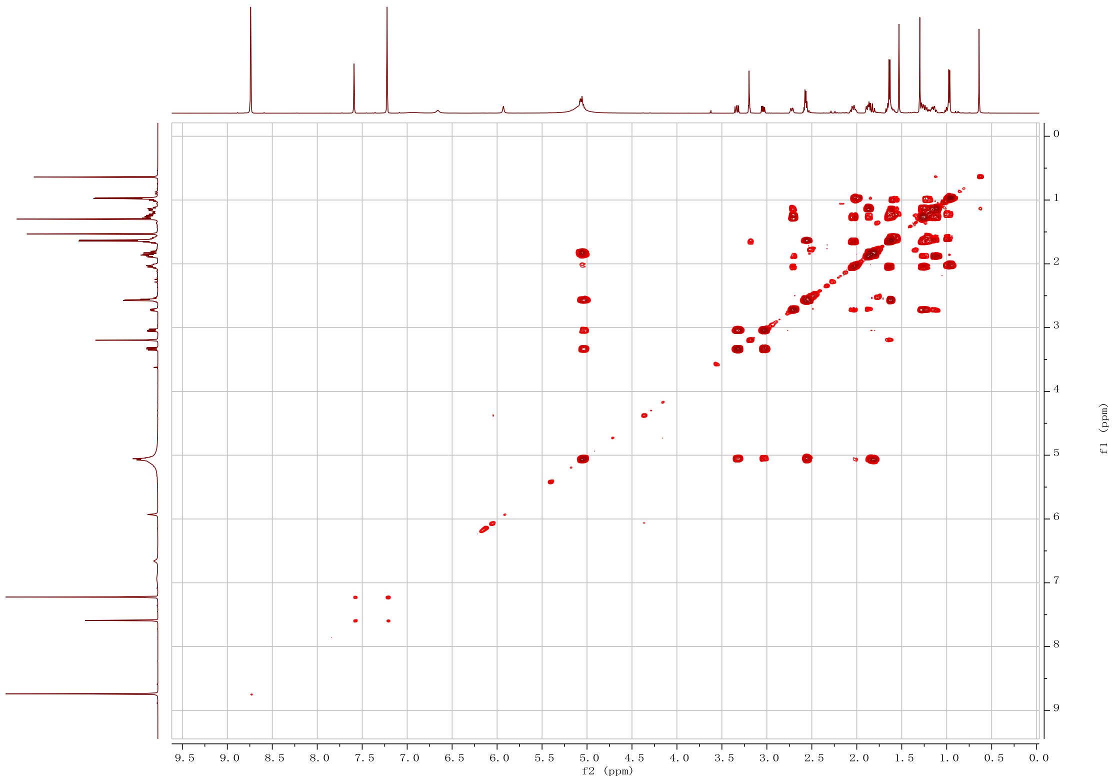

Figure S88. ^1H-^1H COSY spectrum of chantriolide P (**11**) in C_5D_5N

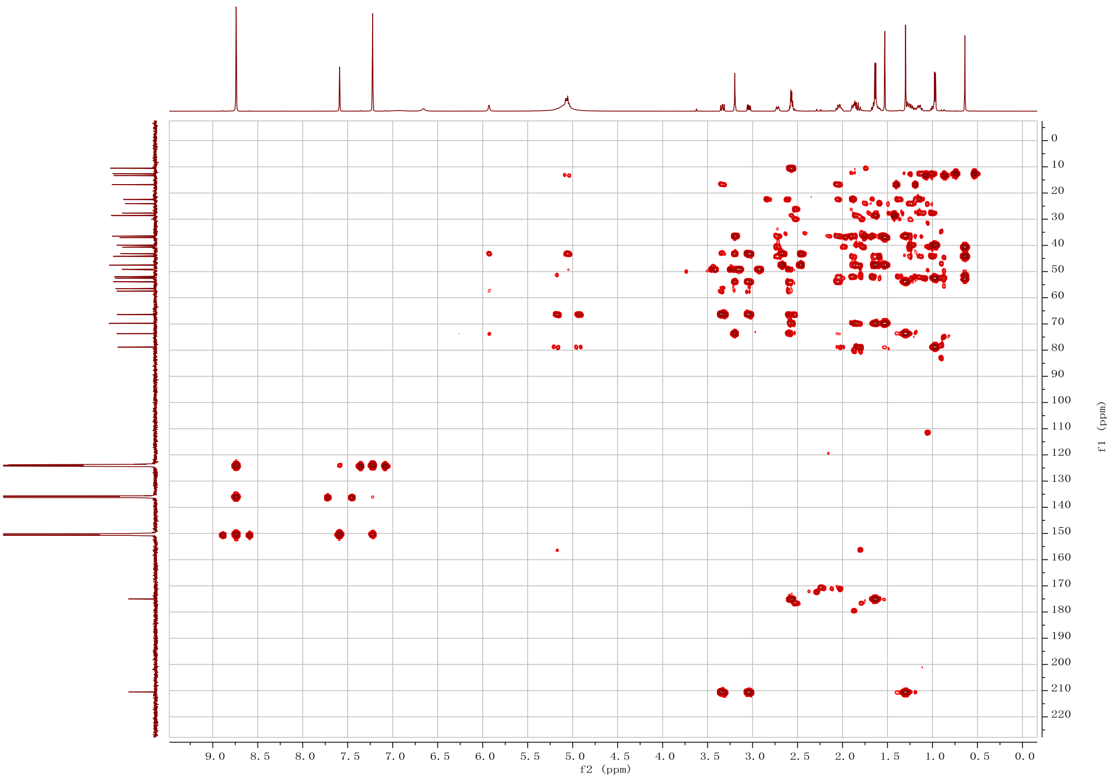

Figure S89. HMBC spectrum of chantriolide P (**11**) in C_5D_5N

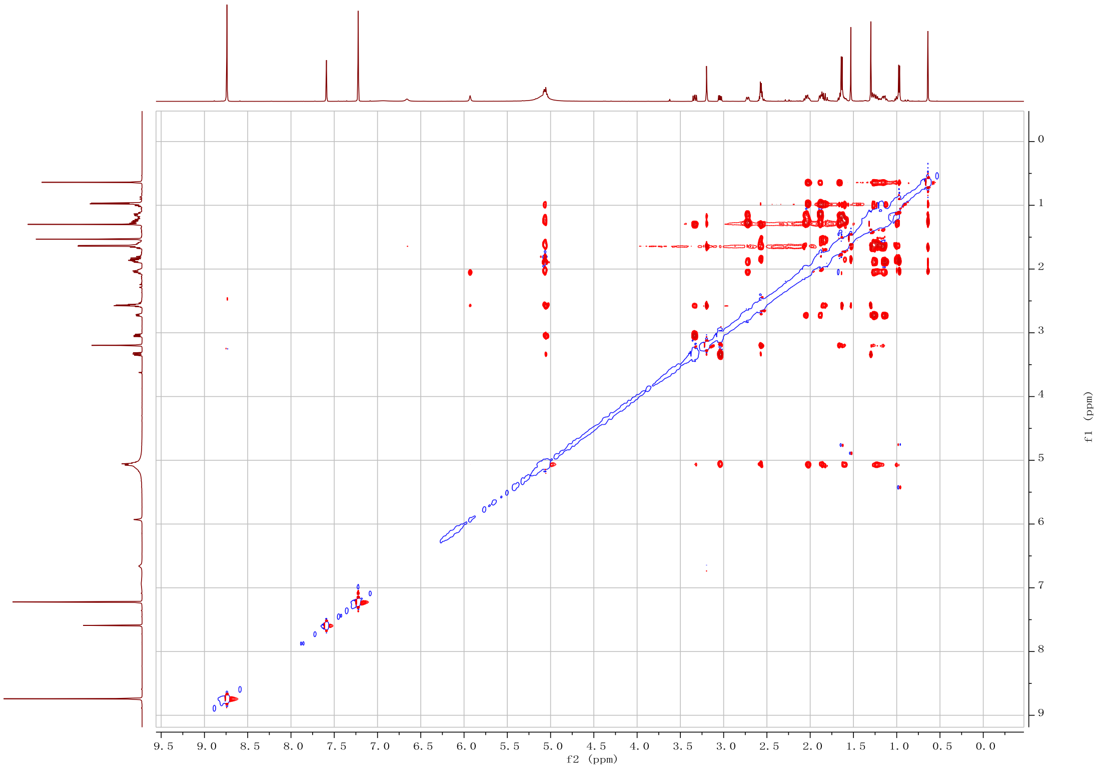

Figure S90. ROESY spectrum of chantriolide P (**11**) in C_5D_5N

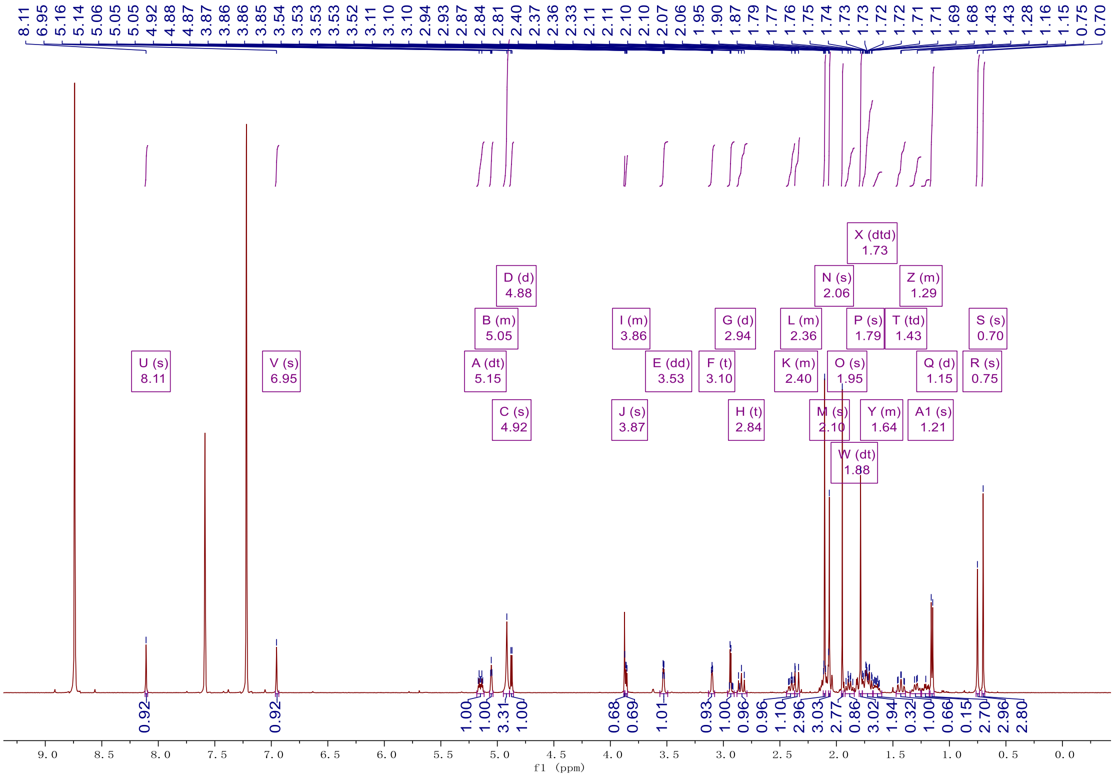

Figure S91. ^{1}H NMR (500 MHz) spectrum of plantagiolide E (**12**) in C_5D_5N

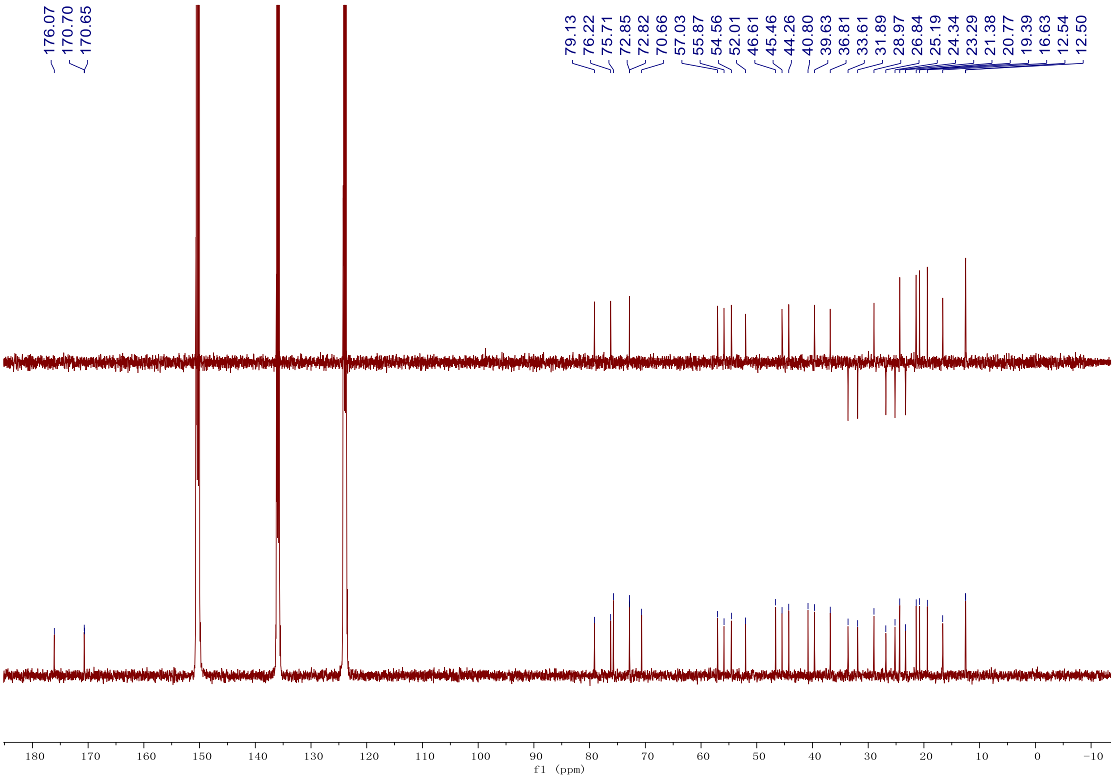

Figure S92. ^{13}C and DEPT-135 NMR (125 MHz) spectra of plantagiolide E (**12**) in C_5D_5N

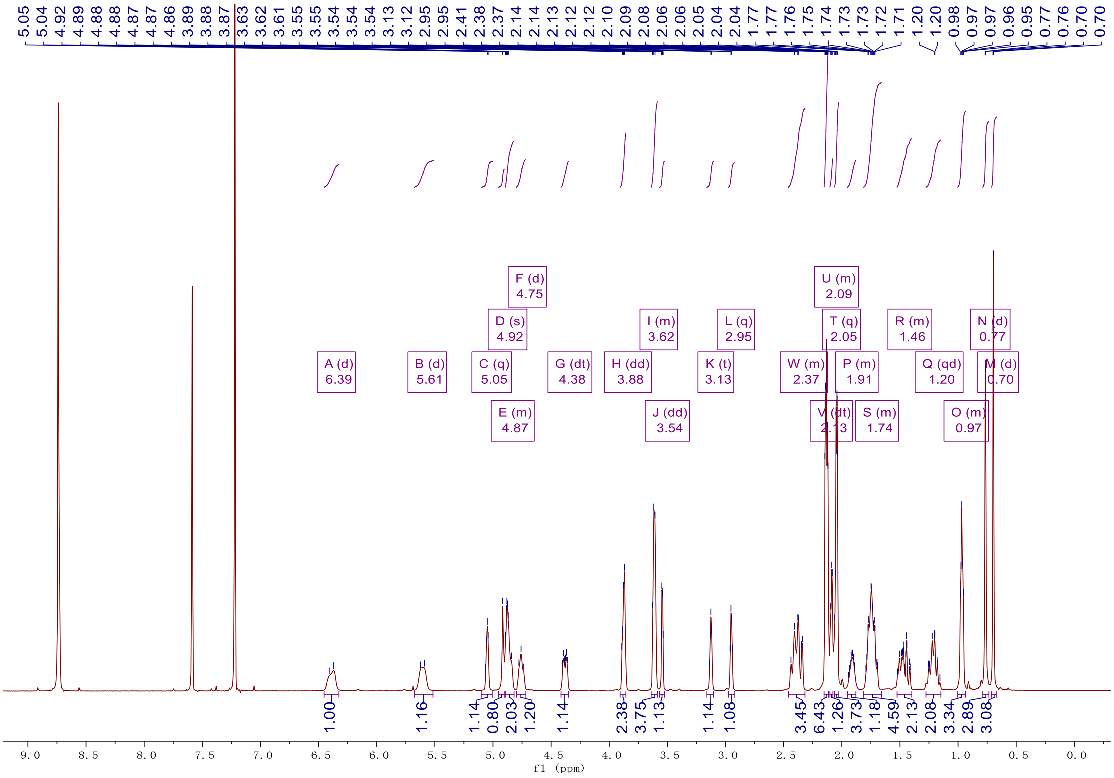

Figure S93. ^{1}H NMR (500 MHz) spectrum of plantagiolide C (**13**) in C_5D_5N

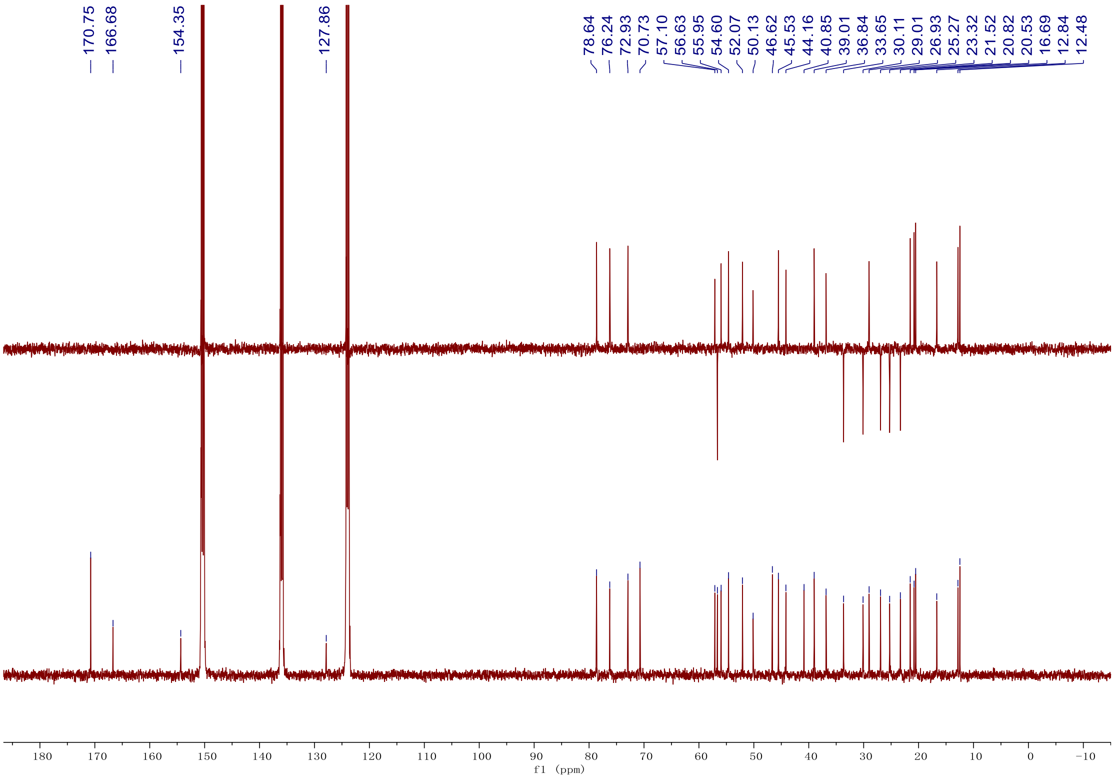

Figure S94. ^{13}C and DEPT-135 NMR (125 MHz) spectra of plantagiolide C (**13**) in C_5D_5N

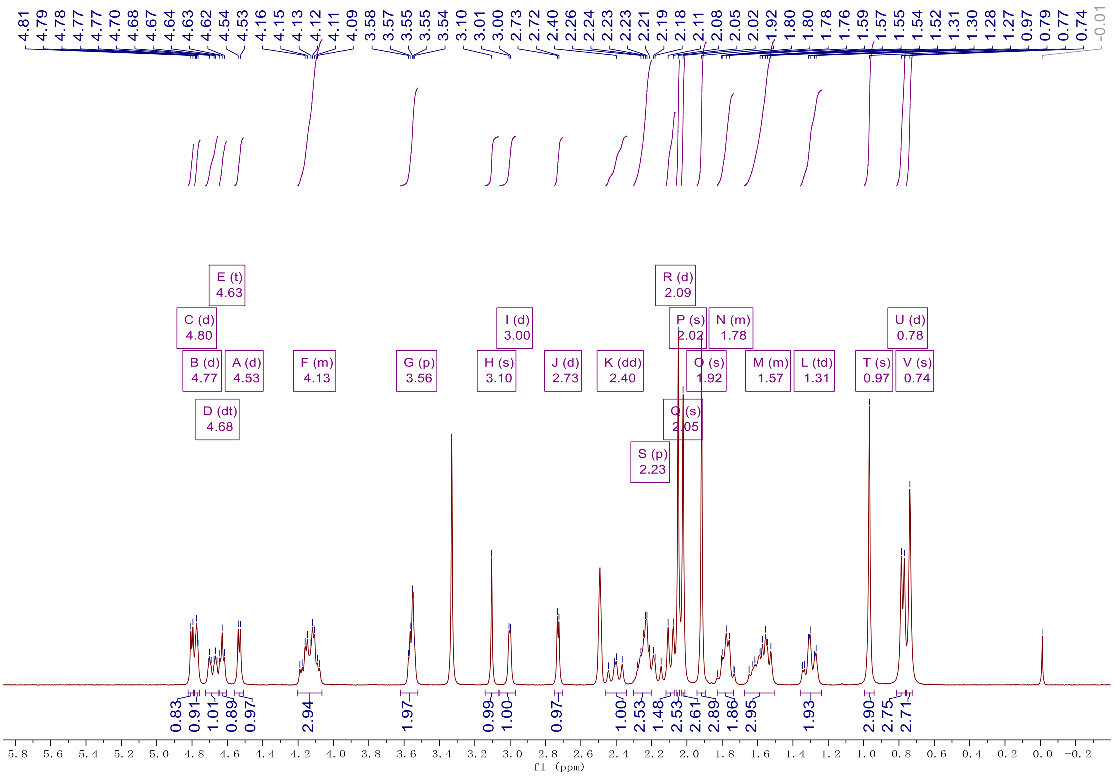

Figure S95. ^{1}H NMR (400 MHz) spectrum of plantagiolide B (**14**) in DMSO-d_6

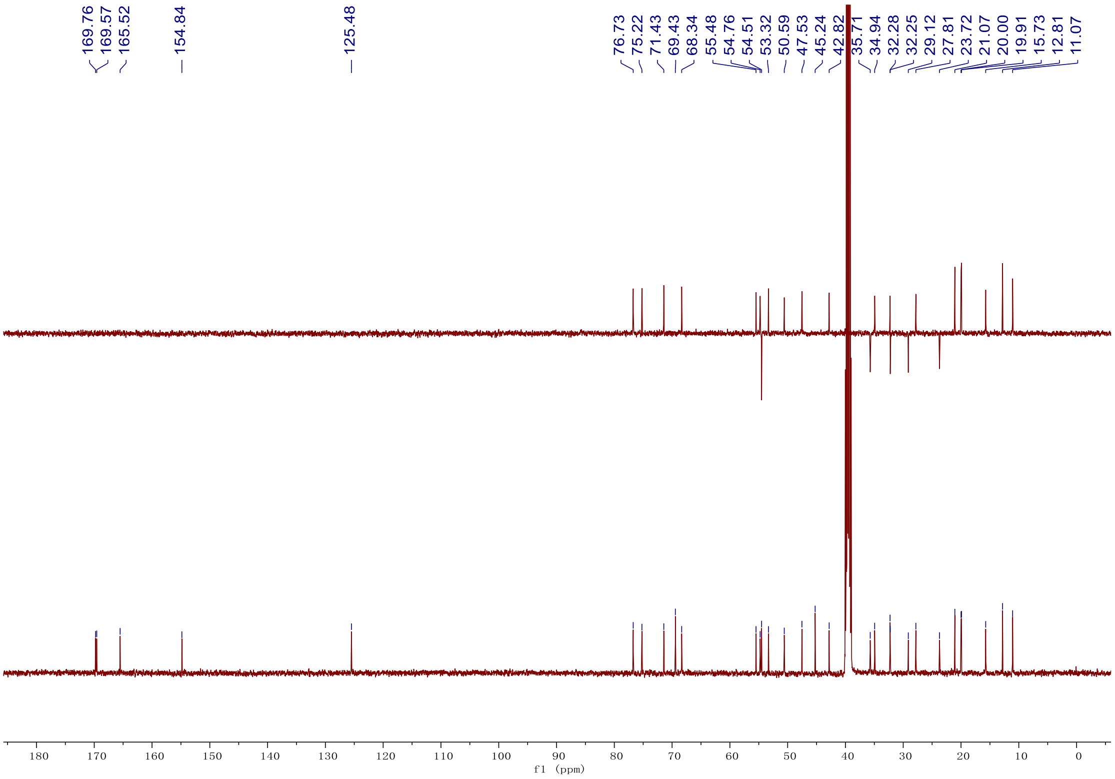

Figure S96. ^{13}C and DEPT-135 NMR (125 MHz) spectra of plantagiolide B (**14**) DMSO-d_6

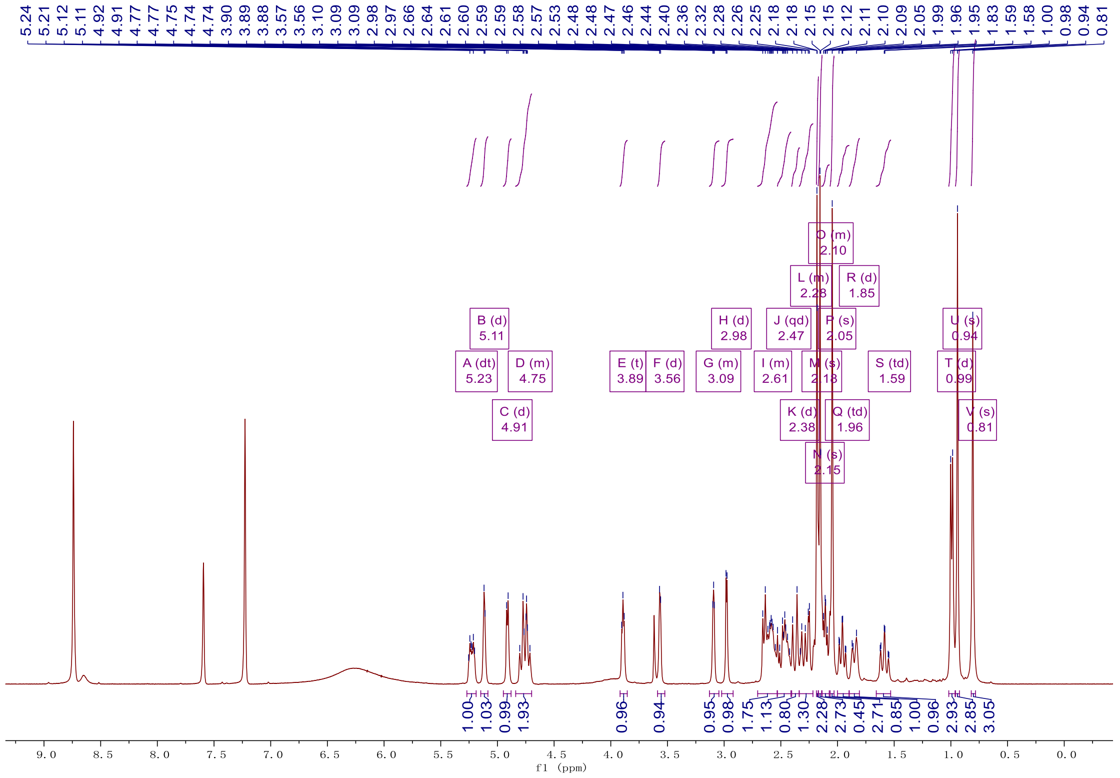

Figure S97. ^{1}H NMR (400 MHz) spectrum of plantagiolide A (**15**) in C_5D_5N

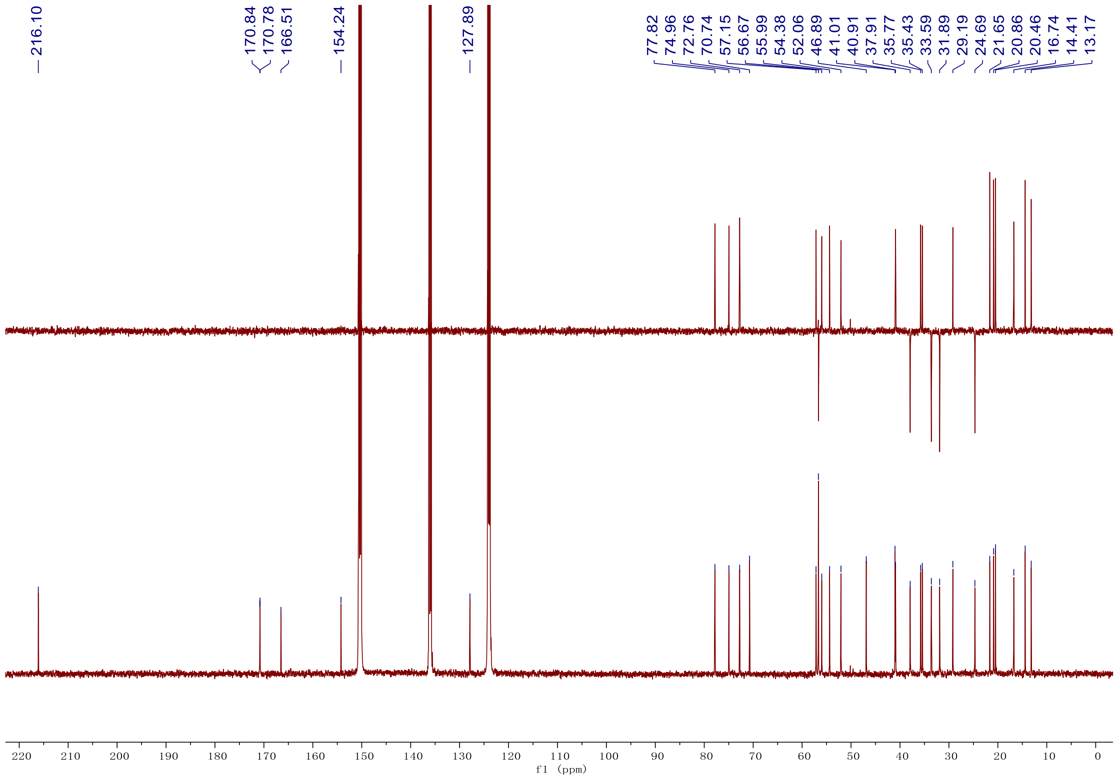

Figure S98. ^{13}C and DEPT-135 NMR (125 MHz) spectra of plantagiolide A (**15**) in C_5D_5N

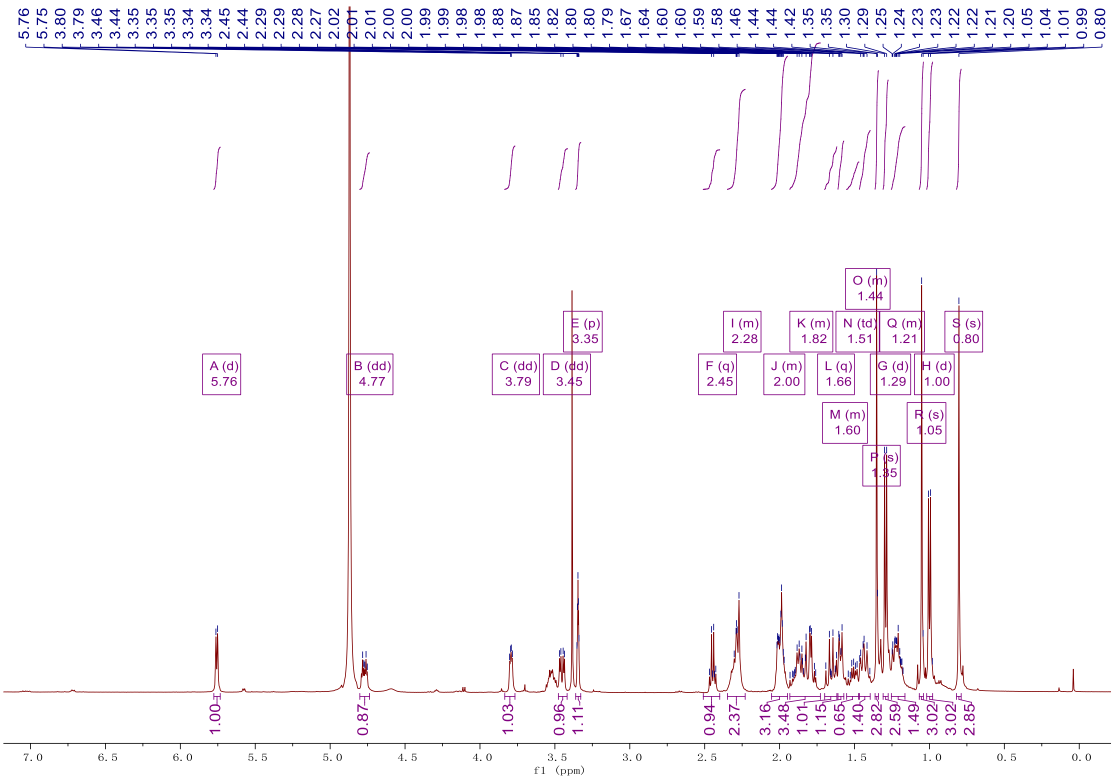

Figure S99. ^{1}H NMR (500 MHz) spectrum of plantagiolide M (**16**) in CD_3OD

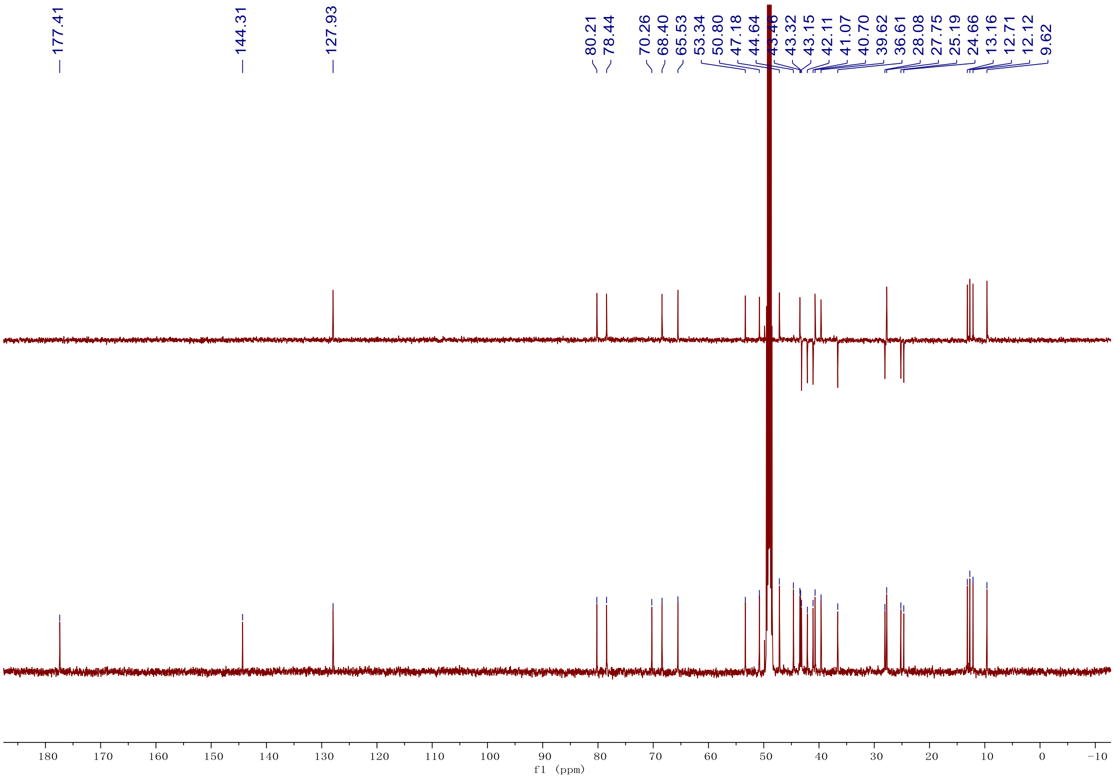

Figure S100. ^{13}C and DEPT-135 NMR (125 MHz) spectra of plantagiolide M (**16**) in CD_3OD

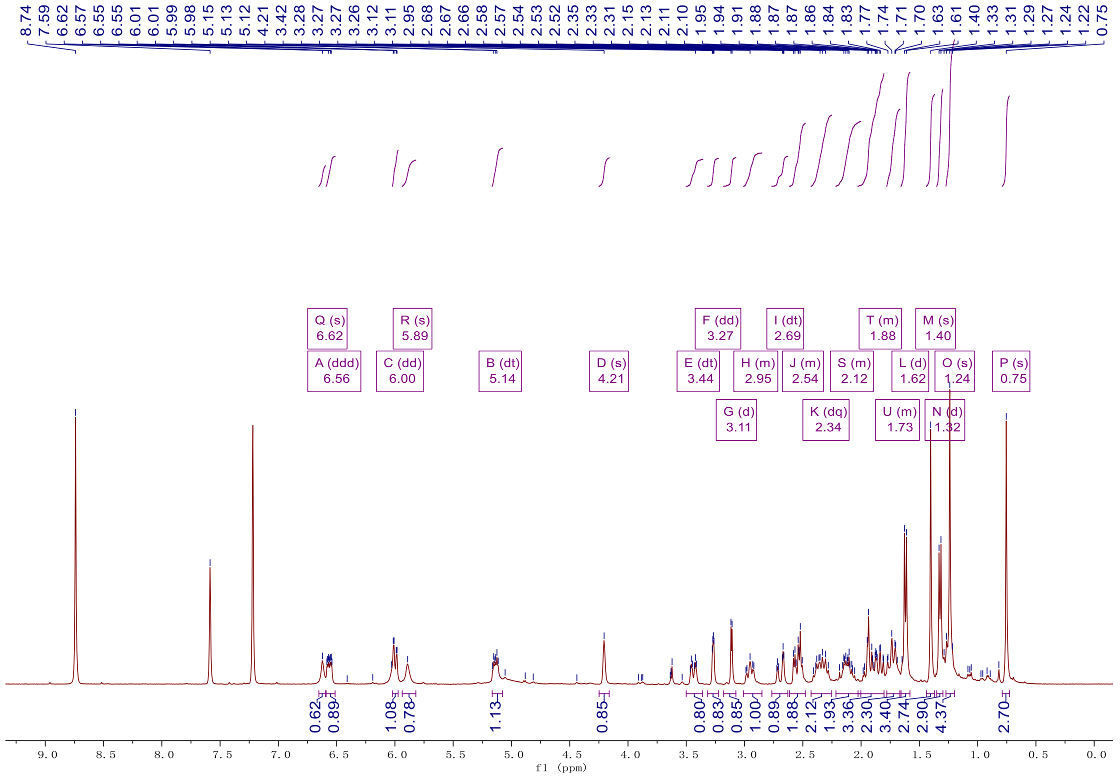

Figure S101. ^{1}H NMR (400 MHz) spectrum of chantriolide D (**17**) in C_5D_5N

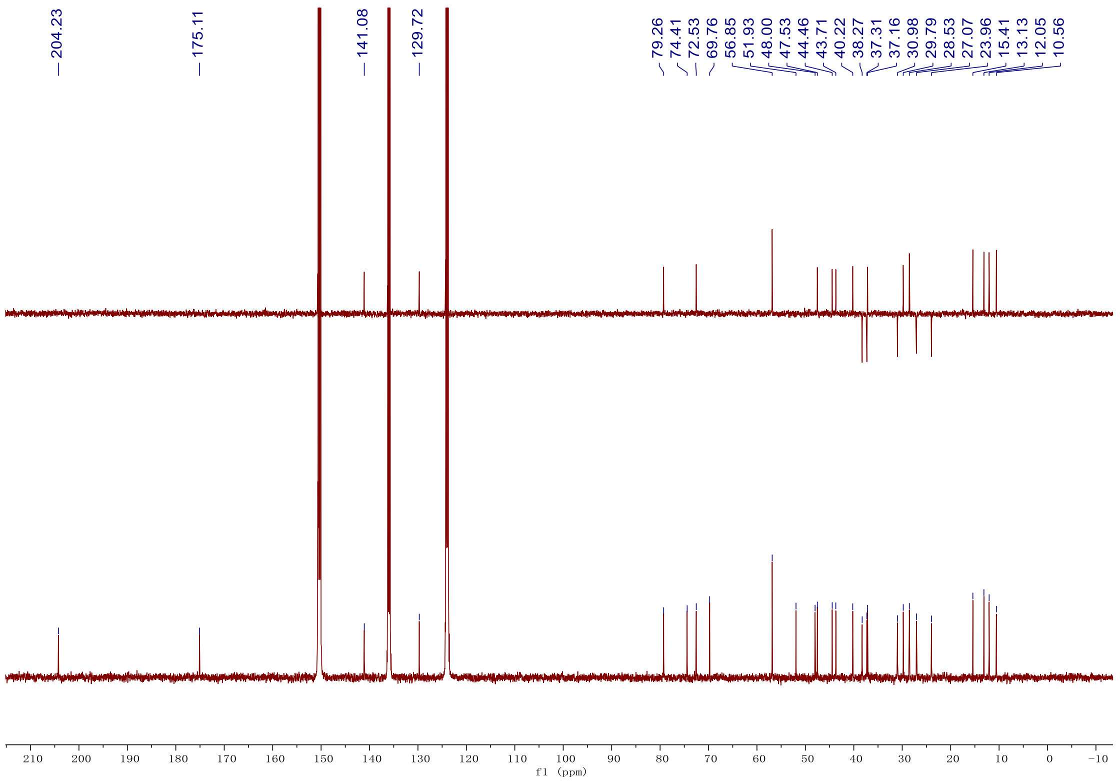

Figure S102. ^{13}C and DEPT-135 NMR (125 MHz) spectra of chantriolide D (**17**) in C_5D_5N

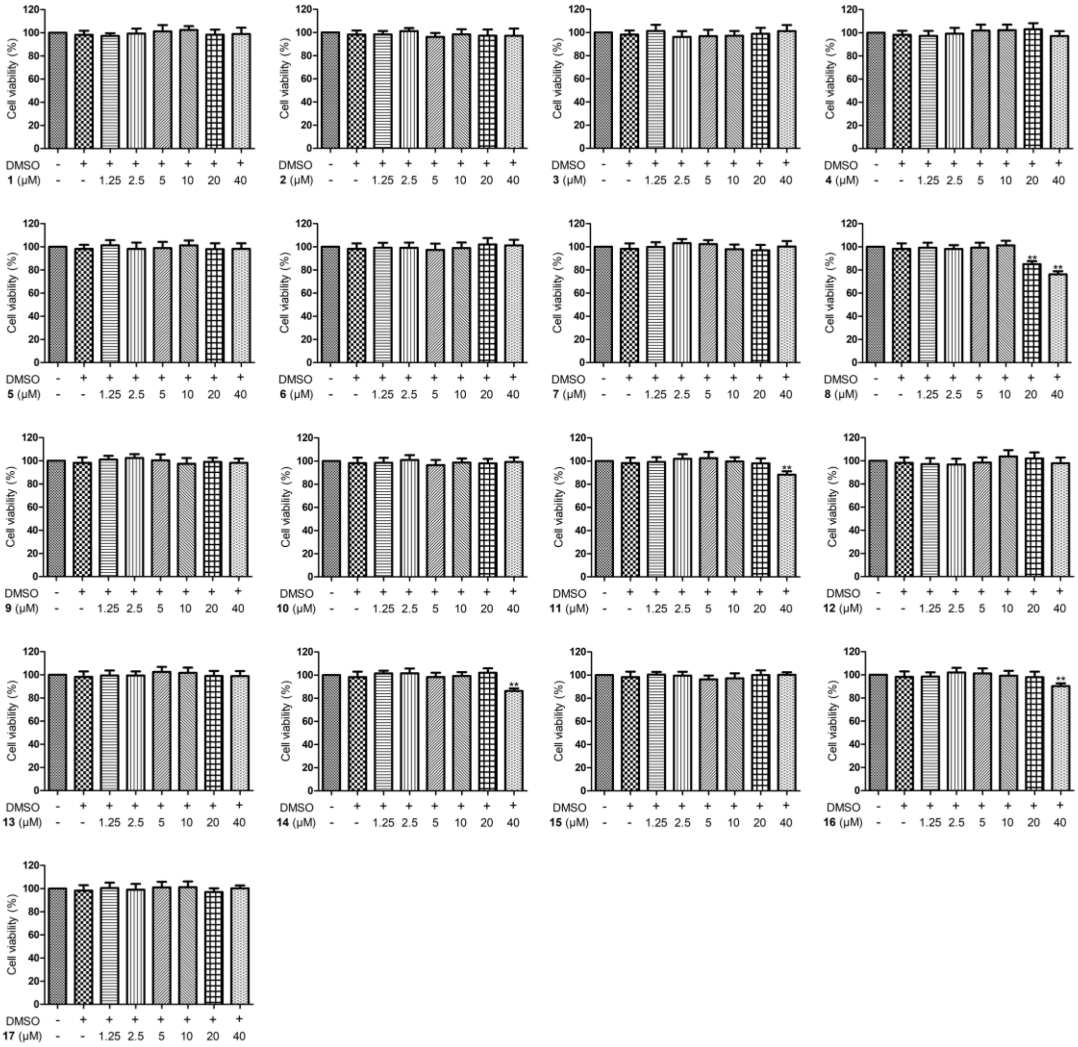

Figure S103. Cytotoxicity of compounds **1–17** on AML-12 hepatocytes. AML-12 cells were treated with different compounds at indicated concentrations for 24 hours. Data are shown as mean ± S.D., n = 3. **P* < 0.05 and ***P* < 0.01 vs. DMSO.
